# Exploring the molecular determinants for subtype-selectivity of 2-amino-1,4,5,6-tetrahydropyrimidine-5-carboxylic acid analogs as betaine/GABA transporter 1 (BGT1) substrate-inhibitors

**DOI:** 10.1038/s41598-020-69908-w

**Published:** 2020-08-03

**Authors:** Stefanie Kickinger, Anas Al-Khawaja, Anne Stæhr Haugaard, Maria E. K. Lie, Francesco Bavo, Rebekka Löffler, Maria Damgaard, Gerhard F. Ecker, Bente Frølund, Petrine Wellendorph

**Affiliations:** 1 Department of Pharmaceutical Chemistry, University of Vienna, Althanstrasse 14, 1090 Vienna, Austria; 2 Department of Drug Design and Pharmacology, Faculty of Health and Medical Sciences, University of Copenhagen, 2100 Copenhagen, Denmark

**Keywords:** Transporters in the nervous system, Pharmacodynamics

## Abstract

We have previously identified 2-amino-1,4,5,6-tetrahydropyrimidine-5-carboxylic acid (ATPCA) as the most potent substrate-inhibitor of the betaine/GABA transporter 1 (BGT1) (IC_50_ 2.5 µM) reported to date. Herein, we characterize the binding mode of 20 novel analogs and propose the molecular determinants driving BGT1-selectivity. A series of *N*_1_-, exocyclic-*N*-, and *C*_4_-substituted analogs was synthesized and pharmacologically characterized in radioligand-based uptake assays at the four human GABA transporters (hGATs) recombinantly expressed in mammalian cells. Overall, the analogs retained subtype-selectivity for hBGT1, though with lower inhibitory activities (mid to high micromolar IC_50_ values) compared to ATPCA. Further characterization of five of these BGT1-active analogs in a fluorescence-based FMP assay revealed that the compounds are substrates for hBGT1, suggesting they interact with the orthosteric site of the transporter. In silico-guided mutagenesis experiments showed that the non-conserved residues Q299 and E52 in hBGT1 as well as the conformational flexibility of the compounds potentially contribute to the subtype-selectivity of ATPCA and its analogs. Overall, this study provides new insights into the molecular interactions governing the subtype-selectivity of BGT1 substrate-inhibitors. The findings may guide the rational design of BGT1-selective pharmacological tool compounds for future drug discovery.

## Introduction

**Figure 1:**
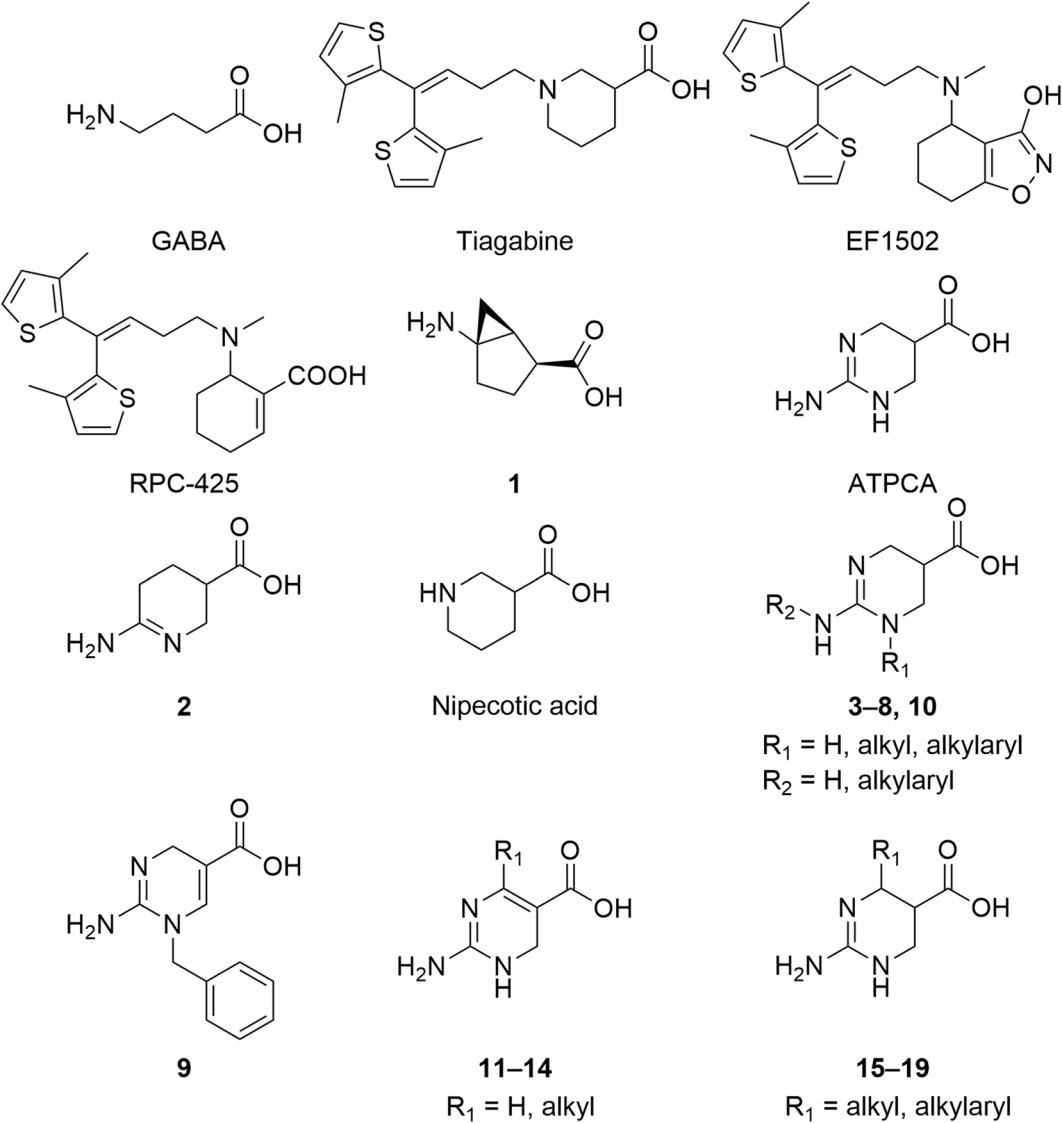
Structures of γ-aminobutyric acid (GABA); the highly selective GAT1 inhibitor, tiagabine; the mixed BGT1/GAT1 inhibitor, EF1502; the moderately selective BGT1 inhibitor, RPC-425; the moderately selective BGT1 substrate-inhibitors, ATPCA and **2**; nipecotic acid, and general structures of the synthesized compounds.

γ-Aminobutyric acid (GABA) (Fig. 1) is the principal inhibitory neurotransmitter in the central nervous system and plays a fundamental role in the overall balance between neuronal excitation and inhibition^1,2^. Dysfunctional GABAergic neurotransmission is implicated in a number of neurological disorders, including epilepsy^3^^,^ insomnia^4^^,^ and stroke^5–7^. As members of the GABAergic system, the four GABA transporters (GATs) play a critical role in the regulation and termination of the GABA-mediated signaling. They function as key proteins in neurotransmitter uptake^8^. GATs belong to the solute carrier 6 (SLC6) transporter family and comprise four subtypes, GAT1, BGT1 (betaine/GABA transporter 1), GAT2, and GAT3^9^. GAT1 is the most abundantly expressed subtype in the mammalian brain and is primarily located in neurons^10^^,^ whereas GAT3 is primarily located in astrocytes^10,11^. While only scarcely expressed in the brain, GAT2 and BGT1 are abundantly expressed in the liver and the kidneys where they mediate the uptake of osmoprotectants such as taurine and betaine, respectively^10,12,13^. In the brain, Schousboe et al.^14^ proposed BGT1 to play a specific role at extrasynaptic sites, and BGT1 inhibitors have been found to be anticonvulsant.

Pharmacological inhibition of GATs leads to extracellular GABA accumulation, which results in an increased inhibitory GABAergic neurotransmission mediated by the activation of GABA receptors^15–17^. This renders GATs attractive targets in the treatment of GABA-implicated disorders, and various inhibitors have been developed^18–22^. The GAT1-selective inhibitor, tiagabine (Gabitril) (Fig. 1), was approved by the FDA in the 1990s for the treatment of partial epileptic seizures^23^. Due to the high expression level of GAT1 and its divergent sequence identity compared to the other GAT subtypes, the majority of developed inhibitors has been designed to target this subtype^20,24^. However, substantial side effects linked to GAT1 inhibition, such as asthenia, dizziness, nervousness, and depression^25–27^, have limited the use of tiagabine, and interest in the non-GAT1 subtypes, especially BGT1, has therefore emerged^18,19,28^.

The GAT1/BGT1 selective inhibitor, EF1502 (*N*-[4,4-bis(3-methyl-2-thienyl)-3-butenyl]-4-(methylamino)-4,5,6,7-tetrahydro-benzo[*d*]isoxazol-3-ol)^29^ and the selective BGT1 inhibitor, RPC-425 (6-((4,4-bis(3-methylthiophen-2-yl)but-3-en-1-yl)(methyl)amino)cyclohex-1-ene-1-carboxylic acid)^30^^,^ (Fig. 1) exhibit anticonvulsive effects in animal models, which are proposed to be BGT1-mediated^6,30–33^. Recently, the conformationally restricted GABA analog, (1*S*,2*S*,5*R*-5-aminobicyclo[3.1.0]hexane-2-carboxylic acid (**1**), has been reported as the first highly selective competitive BGT1 inhibitor with submicromolar potency (IC_50_ 590 nM)^34^. However, its mode of inhibition and its anticonvulsive potential remain elusive. Given the reported anticonvulsive effect of several BGT1 inhibitors, the supposed extrasynaptic location of BGT1^6,14^, and abundant liver and kidney expression, further clarification of the potential role of BGT1 as a drug target is needed. This is even more important given the contradictory findings that BGT1-knockout mice do neither show altered seizure susceptibility nor intolerance to salt treatment^13,35^.

The development of highly selective non-GAT1 inhibitors has been hampered by the high sequence identity of BGT1, GAT2 and GAT3^36,37^. Nevertheless, we have previously succeeded in developing a series of cyclic 2-amino-tetrahydropyrimidine/pyridine compounds by utilizing the GAT1-selective substrate, nipecotic acid (Fig. 1), as a template. These compounds were identified as substrate-inhibitors with pronounced selectivity towards BGT1 over the other GATs^38^. Within the series, 2-amino-1,4,5,6-tetrahydropyrimidine-5-carboxylic acid (ATPCA) and the corresponding amidine analog (6-amino-2,3,4,5-tetrahydropyridine-3-carboxylic acid, **2** (Fig. 1) displayed the highest potencies towards BGT1 (IC_50_ 2.5 µM and 9.8 µM, respectively)^38^. However, the molecular determinants for the observed BGT1-selectivity remain elusive.

In this study, we evaluate the structure–activity relationship (SAR) of a new series of ATPCA analogs and examine their mechanism of uptake inhibition. Furthermore, to explore the molecular interactions governing BGT1-selectivity, we perform mutational studies guided by computational docking and molecular dynamics simulations.

## Results and discussion

### Chemistry

**Figure 2:**
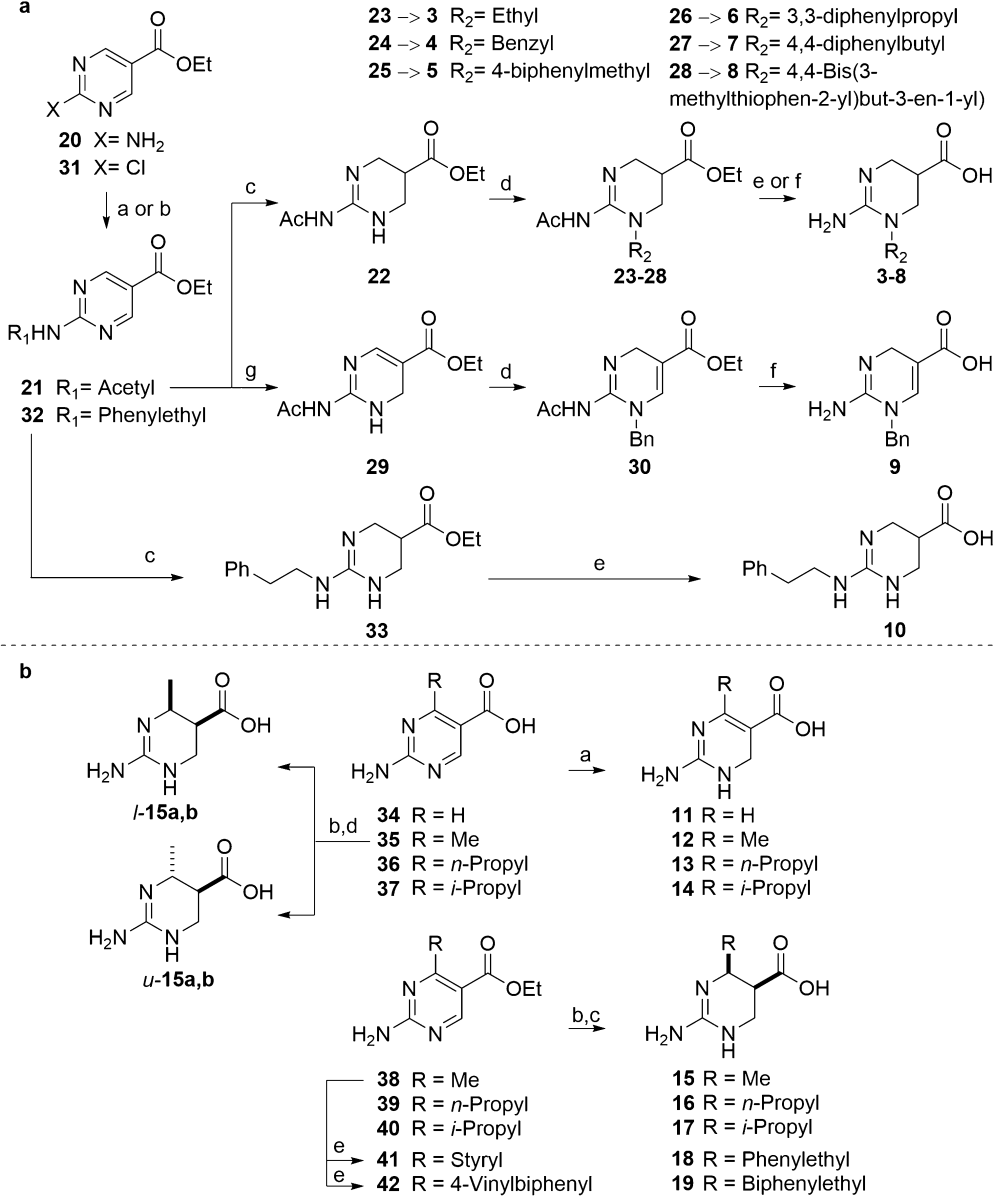
(**a**) Synthesis of the *N*_1_-alkylated target compounds **3–9** and of the exocyclic *N*-alkylated target compound **10**. Reagents and conditions: (a) Ac_2_O, reflux (**21**); (b) 2-phenylethan-1-amine, TEA, 2-propanol, 100 °C (**32**); (c) H_2_, Pd/C, EtOH, AcOH (**22**, **33**); (d) Cs_2_CO_3_, KI, R_2_-Br, DMF (**23–27**, **30**) or Li_2_CO_3_, AcOiPr, R_2_-OMs, reflux, 12 h (**28**); (e) HCl (4 M), 80 °C (**3–7**, **10**); (f) NaOH (0.5 M), 90 °C, 6 h (**8**, **9**); (g) TFA, Et_3_SiH (**29**). (**b**) Synthesis of the *C*_4_-substituted target compounds **11–19**. Reagents and conditions: (a) TFA, Et_3_SiH, DCM (**11–14**); (b) H_2_, Pd/C, MeOH, AcOH; (c) HCl (4 M), 80 °C (**15–19**); (d) preparative chiral HPLC (*l*-**15a,b** and *u*-**15a,b**). (e) Aromatic aldehyde, glacial acetic acid, 130 °C (**41**, **42**).

A series of ATPCA analogs (Fig. 1) was synthesized to elucidate the SAR of subtype-selective hBGT1 inhibition. The structural design relied upon systematically investigating the effect of substituents in the 2-amino-tetrahydropyrimidine scaffold and conformationally constraining the pharmacophoric elements. Inspired by previous reports demonstrating that the introduction of bulky lipophilic substituents at the amino group of small GABA uptake inhibitors increases potency^20^, we synthesized a series of *N*_1_ and exocylic-*N* substituted analogs of ATPCA, as outlined in Fig. 2.

The series of *N*_1_-alkylated analogs (**3–9**) was synthesized from commercially available ethyl 2-aminopyrimidine-5-carboxylate **20** (Fig. 2a). Acetyl protection of the guanidino primary amine afforded **21**, which was hydrogenated using catalytic Pd/C. The resulting tetrahydropyrimidine intermediate **22** was alkylated using either cesium carbonate and an appropriate alkyl bromide, yielding the *N*_*1*_-alkylated compounds **23–27**^39^, or with lithium carbonate and 4,4-bis(3-methylthiophen-2-yl)but-3-en-1-yl methanesulphonate, providing **28**, which carries the same *N*-substituent as EF1502^29^. Deprotection afforded the target compounds **3–8**. To probe the influence of conformational rigidity in *N*_1_/*N*_3_-alkylated compounds, intermediate **21** was also reduced to the almost flat dihydropyrimidine **29,** followed by *N*-benzylation and deprotection to provide the target compound **9**.

To avoid polyalkylation, the 2-amino alkylated analog **10** was achieved by a coupling between the commercially available ethyl 2-chloropyrimidine-5-carboxylate **31** and 2-phenylethan-1-amine to give **32** (Fig. 2a)^40^. Catalytic hydrogenation (**33**), followed by hydrolysis under acidic conditions, afforded the target compound **10**.

To probe the impact of conformational flexibility of the tetrahydropyrimidine scaffold on BGT1 activity, the dihydropyrimidine analogs of ATPCA **11–14** were pursued. Selective reduction of the 2-aminopyrimidines **34–37** was achieved in excellent yields when applying triethylsilane and trifluoroacetic acid (Fig. 2b), as reported by Baskaran et al.^41^. No trace of the fully reduced tetrahydropyrimidine was detected, probably due to conjugative stabilization of the 2-amino-dihydropyrimidine by the 5-carboxylic acid.

The corresponding tetrahydropyrimidines **15–17** were pursued by catalytic hydrogenation of the 2-aminopyrimidine-5-carboxylic acids (**35–37**) using Pd/C (Fig. 2b). However, all attempts to obtain the target 4-substituted 2-amino-tetrahydropyrimidine-5-carboxylic acid analogs resulted in rapid decarboxylation. Therefore, catalytic hydrogenation, using Pd/C starting from the commercially available pyrimidine esters **38–40**, was performed, followed by acidic hydrolysis. Only the *cis* isomers were detected as products in the hydrogenation step.

Based on initial pharmacological characterization of the 4-methyl substituted 2-amino-tetrahydro-pyrimidine analog **15** (Table 1), we decided to investigate the impact of stereochemistry on the observed BGT1 activity and selectivity. To avoid acidic hydrolysis following enantiomeric separation of **15**, we used the carboxylic acid **35** as the starting compound, carefully monitoring the hydrogenation reaction. Catalytic hydrogenation of **35**, using Pd/C in 28 h, afforded a mixture of *cis* (70%), *trans* (10%), and decarboxylated product (20%), based on ^1^H-NMR (data not shown). The four isomers were separated on a ChirobioticT preparative column (25 cm × 21.2 mm, 5 µm), using a flow of 5 ml/min and EtOH/H_2_O (NH_4_OAc 20 mM, pH 4) (30:70, v/v) as a mobile phase (Fig. 2b). The enantiomeric purity was > 95%. Unfortunately, the absolute configuration of these two pairs of enantiomers could not be determined. Our attempt to crystallize the single stereoisomers in order to determine the absolute configuration through X-Ray crystallography was not successful. We therefore refer to the enantiomeric pairs as *l-15* and *u-15* (i.e. (R*R*)-**15** and (R*S*)-**15**, respectively) indicating the relative stereochemistry and to the single stereoisomers as *l*-**15a**, *l*-**15b**, *u*-**15a** and *u*-**15b**.

**Table 1:** [^3^H]GABA uptake inhibitory activities of ATPCA and analogs at recombinant hGATs. Structures are depicted in Fig. 1.

**IC**_**50**_ (pIC_50_ ± S.E.M.) (µM)						
	R_1_	R_2_	hBGT1	hGAT1	hGAT2	hGAT3
ATPCA	–	–	2.5 (5.60 ± 0.11)^a^	> 1,000^c^	41 (4.39 ± 0.09)^a^	99 (4.00 ± 0.03)^a^
**2**	–	–	13 (4.89 ± 0.02)	216 (3.67 ± 0.01)	> 1,000^c^	> 1,000^c^
**3**	Ethyl	H	86 (4.07 ± 0.02)^b^	> 1,000^c^	> 1,000^c^	> 1,000^c^
**4**	Benzyl	H	163 (3.79 ± 0.03)^b^	> 1,000^c^	105 (3.98 ± 0.04)^b^	117 (3.93 ± 0.005)^b^
**5**	4-Biphenylmethyl	H	50 (4.30 ± 0.03)	1,044 (2.98 ± 0.03)^b^	382 (3.42 ± 0.05)^b^	396 (3.42 ± 0.10)^b^
6*	3,3-Diphenylpropyl	H	> 1,000^c^	> 1,000^c^	> 1,000^c^	> 1,000^c^
7*	4,4-Diphenylbutyl	H	> 1,000^c^	> 1,000^c^	> 1,000^c^	> 1,000^c^
**8**	4,4-bis(3-Methyl-2-thienyl)3-butenyl	H	621^d^ (3.21 ± 0.03)^d^	N.D	N.D	N.D
**9**	–		> 1,000	N.D	N.D	N.D
**10**	H	Phenylethyl	> 1,000^c^	439 (3.37 ± 0.07)	> 1,000^c^	> 1,000^c^
**11**	H	–	48 (4.33 ± 0.08)	> 1,000^c^	> 1,000^c^	> 1,000^c^
**12**	Methyl	–	> 1,000^c^	> 1,000^c^	> 1,000^c^	> 1,000^c^
**13**	*N*-propyl	–	> 1,000^c^	> 1,000^c^	530 (3.30 ± 0.10)^b^	> 1,000^c^
**14**	*i*-Propyl	–	> 1,000^c^	> 1,000^c^	1,010 (3.02 ± 0.10)^b^	> 1,000^c^
*l*-**15**	Methyl	–	42 (4.38 ± 0.03)	N.D	N.D	N.D
*l-15a*	Methyl	–	34 (4.47 ± 0.06)	> 1,000^c^	> 1,000^c^	> 1,000^c^
*l-15b*	Methyl	–	442 (3.38 ± 0.09)^b^	> 1,000^c^	> 1,000^c^	> 1,000^c^
*u-15a*	Methyl	–	292 (3.57 ± 0.12)^b^	> 1,000^c^	> 1,000^c^	> 1,000^c^
*u*-**15b**	Methyl	–	85 (4.11 ± 0.11)	> 1,000^c^	> 1,000^c^	> 1,000^c^
**16**	*N*-Propyl	–	> 1,000^c^	> 1,000^c^	601 (3.23 ± 0.05)	> 1,000^c^
**17**	*i*-Propyl	–	> 1,000^c^	> 1,000^c^	> 1,000^c^	> 1,000^c^
*l*-**18**	phenylethyl	–	> 1,000^c^	> 1,000^c^	> 1,000^c^	> 1,000^c^
*l*-**19**	4-biphenylethyl	–	> 1,000^c^	> 1,000^c^	> 1,000^c^	> 1,000^c^

The compounds were tested for their ability to inhibit the uptake of 30 nM [^3^H]GABA for 3 min at recombinant hGATs stably expressed in CHO Flp-In cells. The experiments were performed in triplicates in at least three independent experiments (compounds marked with * were only tested in two independent experiments). N.D., not determined. All compounds are racemic mixtures unless stated otherwise. Concentration response curves for selected compounds can be found in the Supplementary Figs. S21 and S22.

^a^Adapted from Al-Khawaja et al.^38^.

^b^The concentration-inhibition curves of these compounds were fitted to the value of 100% uptake inhibition in the presence of GABA (3 mM).

^c^These compounds displayed less than 50% [^3^H]GABA uptake inhibition at the highest tested concentration of 1,000 µM.

^d^These compounds were tested in tsA201 cells transiently expressing hBGT1.

An alternative approach had to be taken for the synthesis of the aromatic substituted analogs **18** and **19**, which were obtained via a condensation reaction between the commercially available ethyl 2-amino-4-methylpyrimidine-5-carboxylate (**38**) and the benzaldehyde or biphenyl-4-carbaldehyde, respectively, yielding the intermediates **41** and **42**. Catalytic hydrogenation using Pd/C afforded the 4-substituted tetrahydropyrimidine analogs, where the *cis* isomers were formed as the major products. Thus, only the *cis*-stereoisomers were isolated and subjected to acidic hydrolysis, yielding the target compounds *l*-**18** and *l*-**19** (Fig. 2b).

### Pharmacology

The synthesized ATPCA analogs (Fig. 1) were evaluated in [^3^H]GABA uptake assays at the four human GATs (hGATs) stably expressed in Chinese hamster ovary (CHO) Flp-In cells (Table 1). All compounds, except for **4**, showing inhibitory activity at hBGT1 (tested in concentrations from 0.1 µM to 1 mM), displayed selectivity for this subtype. Introduction of an ethyl, a benzyl, or even a 4-biphenylmethyl group in the *N*_1_ position (**3**, **4**, and **5**) was tolerated, but decreased the inhibitory potencies 20–65 times compared to ATPCA. Interestingly, **4** was equipotent at all subtypes except hGAT1, whereas **5** showed at least 7 times increased selectivity towards hBGT1compared to **4**. Since we were not able to generate full inhibition curves for **3**, **11**, *l*-**15a,b**, and *u-15a,b* at hGAT1-3, we can only estimate their hBGT1 selectivity. Based on the less than 50% inhibition at the highest concentration tested (1,000 µM), the analogs **3**, **11** *l-15a*, and *u*-**15b** showed at least 12, 21, 29, and 12 times increased subtype-selectivity for hBGT1, respectively. For *l-15b* and *u-15a*, however, the selectivity was only around 2–3 times more pronounced for hBGT1. In contrast to previously reported structurally related GABA uptake inhibitors^42^^,^ increasing the carbon chain length and changing the position of the distal phenyl group led to a loss of inhibitory activity at any of the hGATs as shown for **6** and **7**, respectively. Likewise, the introduction of a phenylethyl substituent in the *exocyclic-N* position (**10**) was detrimental to the inhibitory activity. The dihydropyrimidine analog **11**, representing a conformationally less flexible core scaffold of ATPCA, showed 19-fold reduced inhibitory activity to that of the parent compound ATPCA. However, introduction of alkyl substituents, such as methyl (**12**), *n*-propyl (**13**), and *iso-*propyl (**14**), led to a complete loss of activity. The same tendency was observed for the corresponding 4-*n*-propyl and 4-*iso-*propyl tetrahydropyrimidine analogs **16** and **17,** respectively. Larger aromatic substituents, such as phenylethyl (*l*-**18**) and 4-biphenylethyl (*l*-**19**), led to a similar loss of inhibitory activity. In contrast, the 4-methyl analog **15** showed activity at hBGT1. Investigation of the four stereoisomers, *l-15a,b* and *u-15a,b* revealed in each of the two *l* and *u* enantiomeric pairs a trend for stereoselective inhibitory activity. However, this was more pronounced for *l-15a,b*, showing a 13-fold difference in inhibitory activity, compared to the respective *trans* isomers *u*-**15a,b**.

**Figure 3:**
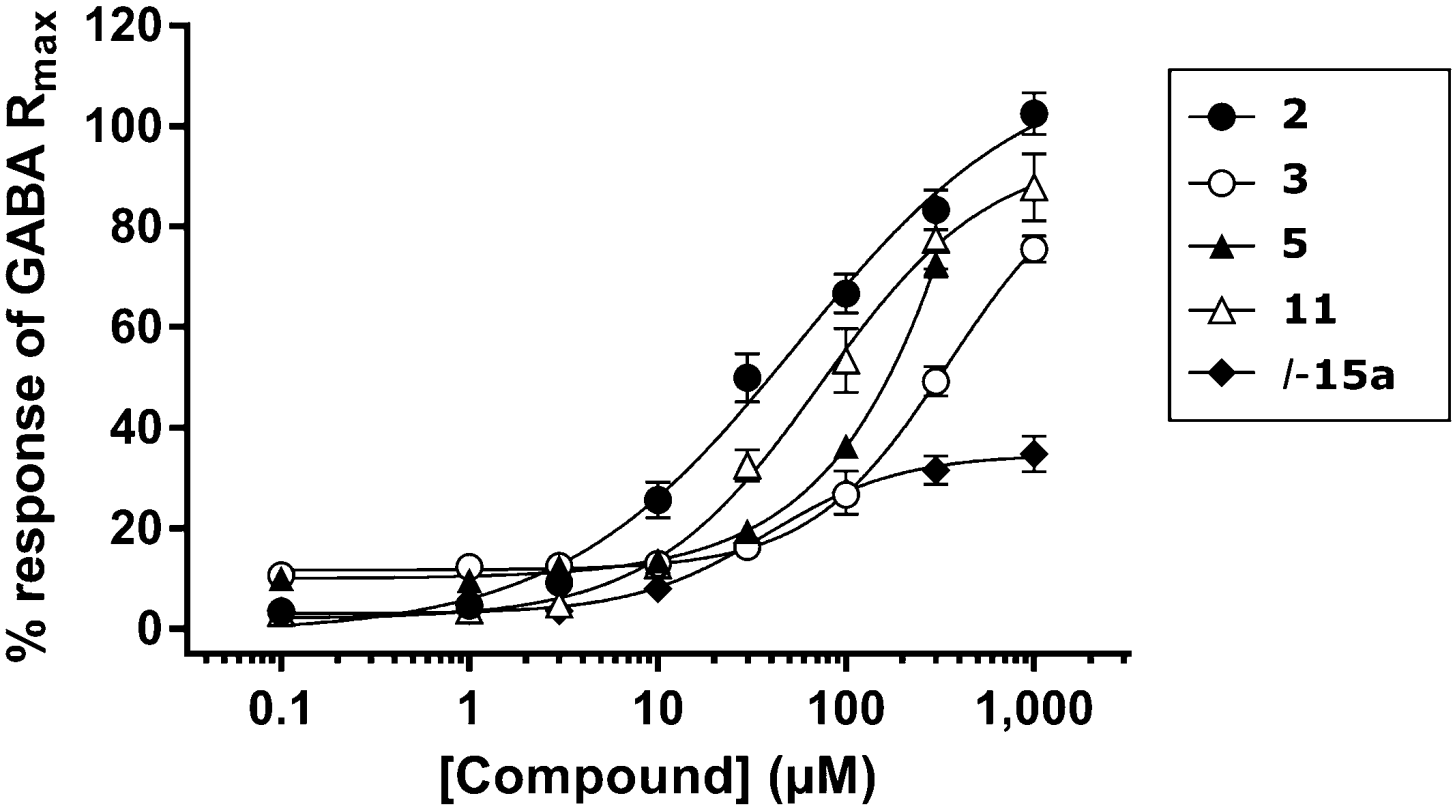
Concentration–response curves for selected ATPCA analogues at hBGT1 stably expressed in CHO Flp-In cells in the FMP assay. Data are normalized to the GABA maximum response (R_max_) and are means ± S.E.M. of three independent experiments performed in triplicates. Mean EC_50_ values in µM (pEC_50_ ± S.E.M.): **2**, 56 (4.3 ± 0.08); 2, 399 (3.4 ± 0.09); **5**, 194 (3.7 ± 0.01) **11**, 75 (4.1 ± 0.05); *l*-**15a**, 40 (4.4 ± 0.04). Mean R_max_ values given as % of GABA R_max_ ± S.E.M.: **2**, 103 ± 3.9; 2, 76 ± 2.7; **5**, 73 ± 2.8; **11**, 95 ± 4.0; *l*-**15a**, 36 ± 2.9. Owing to solubility limitations, a top plateau could not be obtained for **5**, and the curve was therefore constrained to GABA R_max_. The fluorescence signals were hBGT1-specific, as no comparable pronounced responses were seen at hGAT1 stably expressed in CHO Flp-In cells, when the highest concentrations were tested (data not shown). This suggested that the fluorescence responses seen were not due to nonspecific effects, such as compound autofluorescence, and that the compounds were truly hBGT1 substrates.

We have previously demonstrated that ATPCA is a substrate for hBGT1, using the fluorescence-based FLIPR Membrane Potential (FMP) assay^38,43^. To examine whether derivatization of ATPCA converted some of the analogs into non-transportable inhibitors of hBGT1, we tested **2**, **3**, **5**, **11**, and *l-15a* in the FMP assay. These compounds showed IC_50_ values below 100 µM in the [^3^H]GABA uptake assay at hBGT1 (Table 1). In the FMP assay, the compounds showed a concentration-dependent increase in the fluorescence signal at hBGT1 stably expressed in CHO Flp-In cells, suggesting that they are all substrates for hBGT1 and that derivatization did not convert the analogs into non-transportable inhibitors. Since all tested compounds are GABA analogs it is very likely that they interact with the orthosteric pocket of the transporter (Fig. 3).

### Identification of the molecular determinants for BGT1-selectivity

To delineate the molecular determinants for the observed BGT1-selectivity, computational docking studies and molecular dynamics (MD) simulations, followed by mutagenesis experiments, were performed. Selected compounds (ATPCA, **2**–**5**, **11**–**14**, *l-15a,b,* and *u-15a,b*) were docked into the orthosteric binding site of an already published outward-occluded homology model of hBGT1^44^ applying an induced fit docking protocol^45^. This substrate specific transporter conformation was chosen because ATPCA and the analogs **2**, **3**, **5**, **11**, and *l-15a* displayed substrate characteristics in the FMP assay. Docking into homology models of hGAT2 or hGAT3 was not performed since all compounds, except for ATPCA and **4** and **5**, showed no activity at these transporters. The docking was performed at pH 7.4 which led to the zwitterionic form of all compounds (see methods section). The poses were analyzed according to an in-house protocol for common-scaffold clustering^46–48^. Briefly, the docking poses were assembled into 33 clusters (Supplementary Fig. S1), where the most populated cluster contained poses of all active compounds except for **4** and **5** (Supplementary Fig. S2). Since all compounds share the scaffold of either ATPCA or **11** (Fig. 1), a single top-scored pose of this cluster, based on the Glide Emodel score^49^, for either of the two compounds was selected for subsequent refinement using MD simulations. The poses were simulated three times for 20 ns to investigate the stability of the protein–ligand interactions.

**Figure 4:**
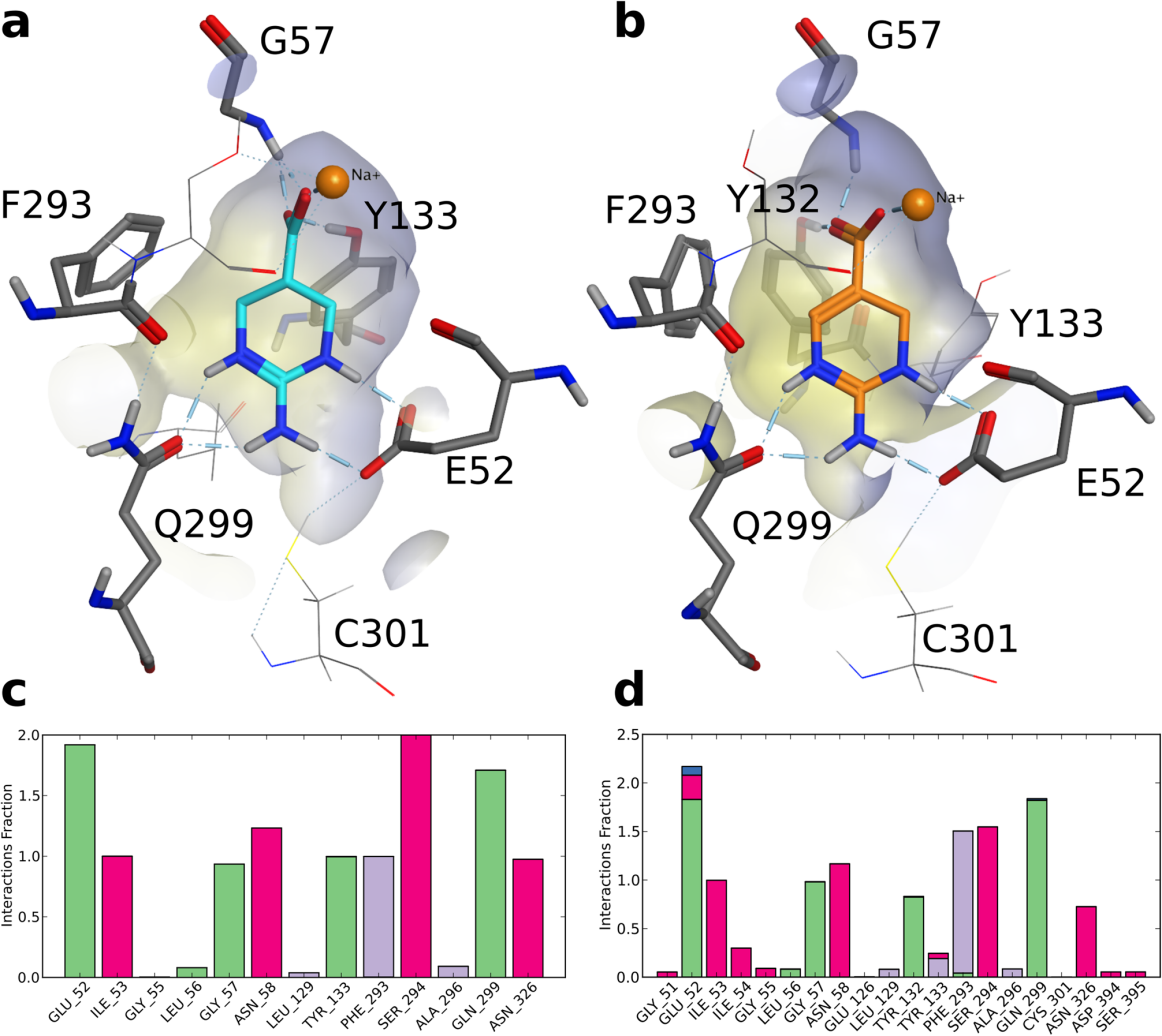
(**a**) Protein–ligand interactions of ATPCA. The best-scored docking pose was refined by 20 ns of molecular dynamics (MD) simulations. The trajectory was clustered according to the ligand, and a representative snapshot of the most highly populated cluster was selected as a final binding pose. The molecular surface is depicted with hydrophilic areas in blue and lipophilic areas in yellow. (**b**) Protein ligand interactions of **11**. The pose was selected according to the same procedure as in (**a**). (**c**) Protein–ligand interaction diagram of one MD simulation of ATPCA (pink, ionic interactions; green, hydrogen bonding; purple, hydrophobic contacts). Two additional replicas gave similar results. An interaction fraction value of 1.0 suggests that during the simulation this interaction is maintained 100% of the time. Values greater than 1.0 are possible due to multiple contacts of the ligand with the same residue. (**d**) Protein–ligand interaction diagram of **11**. Two additional replicas gave similar results.

The MD results of the selected ATPCA pose showed stable hydrogen bonding between ATPCA’s guanidine moiety and the side chains of Q299 and E52 (Fig. 4a,c and Supplementary Fig. S3). Q299 constitutes a unique residue in hBGT1, corresponding to L300/L294/L314 in hGAT1/hGAT2/hGAT3, whereas E52 only differs in hGAT1, corresponding to Y60^37,50,51^. Interestingly, the corresponding residues in both positions were already identified by others to play a role in substrate specificity in homologous transporters^52,53^. The *N*_3_ nitrogen of ATPCA’s guanidine moiety forms a π-cationic interaction with the highly conserved F293, which is part of the extracellular lid of hBGT1^37^. ATPCA’s carboxyl group coordinates the sodium ion (Na1) and undergoes hydrogen bonding with G57, which is conserved among the GATs, as well as with the sidechain of Y133, which is also a highly conserved residue since it is part of the extracellular lid^37,50,54^. The docking and MD results of **11** showed a similar interaction pattern to that of ATPCA (Fig. 4b,d, Supplementary Fig. S3) with the only difference that F293 shows alternatingly π-cation interactions with *N*_3_ and π-face-edge interactions with the ring, and the carboxyl group forms hydrogen bonds with Y132 instead of Y133, which is also conserved among the GATs^37,50,51^. This binding hypothesis, however, cannot explain the activity difference of ATPCA compared to **11** (IC_50_ 2.5 μM and IC_50_ 48 μM, respectively). Nevertheless, we hypothesize that **11**, due to its more rigid scaffold, may adopt energetically less favorable conformations during the transport cycle resulting in a 19-fold lower activity.

### The molecular basis for the experimental SAR

The MD-refined docking poses outlined in Fig. 4 served as a basis for identifying the molecular determinants for subtype-selectivity and for discussing the SAR of the remaining compounds of the dataset. Accordingly, **2** contains one nitrogen less in the ring and is therefore missing one hydrogen bond donor, thus showing fivefold decreased potency (Table 1). The ethyl group of **3** can be accommodated in the pocket, but sterically interferes with the hydrogen bonding of the guanidine moiety and the side chains of E52 and Q299, resulting in a 34-fold decreased activity. The exocyclic-*N*-alkylated **10** shows no activity due to steric hindrance by C301 in the lower part of the orthosteric pocket. The 4-alkyl analogs **12, 13,** and **14** interfere with F293 and are sterically too demanding to fit into the small pocket. The same steric hindrance is observed for **16**–**19,** which share the same flexible scaffold as ATPCA, but also contain bulky substituents in position 4. For *l-15a,b* and *u-15a,b*, the same binding mode as for ATPCA is predicted. Unfortunately, the absolute configuration at the stereocenters bearing the carboxyl and the methyl group is unknown, which precludes a detailed interpretation of the activity differences observed. We assume that compared to **12**, the missing double bond may allow more flexibility in positioning the methyl group enabling a better fit of *l-15a,b* and *u-15a,b* into the pocket. Moreover, the *l* and *u* enantiomeric pairs show stereoselective activity, where the *l-15a,b* and the *u-15a,b* enantiomers differ by 13 and 3 times, respectively. These activity differences underline the limited space that is available in this part of the pocket.

**Figure 5:**
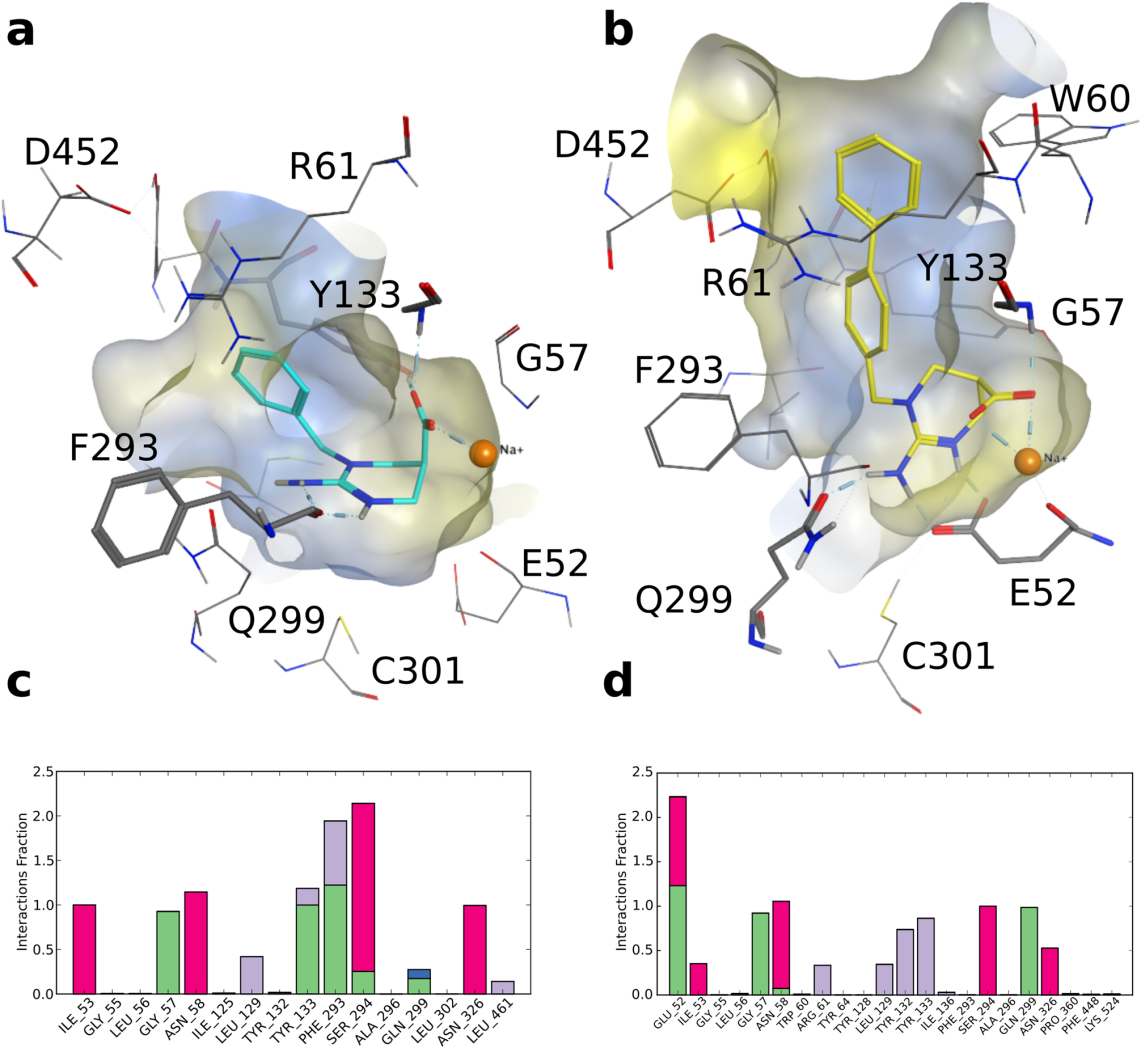
(**a**) Protein–ligand interactions of **4**. The best-scored docking pose was refined by 20 ns of molecular dynamics (MD) simulations. The trajectory was clustered according to the ligand, and a representative snapshot of the most highly populated cluster was selected as a final binding pose. The molecular surface is depicted with hydrophilic areas in blue and lipophilic areas in yellow. (**b**) Protein–ligand interactions of **5**. The pose was selected according to the same procedure as in (**a**). (**c**) Protein–ligand interaction diagram of one MD simulation of **4** (pink, ionic interactions; green, hydrogen bonding; purple, hydrophobic contacts; blue, water contacts). Two additional replicas gave similar results. An interaction fraction value of 1.0 suggests that during the simulation this interaction is maintained 100% of the time. Values greater than 1.0 are possible due to multiple contacts of the ligand with the same residue. (**d**) Protein–ligand interaction diagram of **5**. Two additional replicas gave similar results.

For the BGT1-active **4** and **5**, we postulate a slightly different binding hypothesis, since the *N*_1_-benzyl and biphenyl substituents are too bulky to fit into the orthosteric pocket of the occluded transporter model. This is supported by the observation that within the most populated cluster, no poses of **4** and **5** are present. Accordingly, **4** and **5** can only be accommodated in the extracellular vestibule of the transporter which is facilitated by the opening of the extracellular gate (residues T133, F293, R61 and D452)^37^. This is quite remarkable because we demonstrated that **5** is still transported which would require the closure of the external gate according to the classical alternating-access transport model^55,56^. According to the docking results, in order to accommodate the bulky substituents in the extracellular vestibule while the carboxyl moiety of **4** and **5** coordinates the sodium ion in the orthosteric pocket, which is considered to be the most plausible placement^37,57^, the carboxyl group needs to shift from an equatorial to an axial conformation allowing the compounds to bend. To further probe this notion and to refine the docking poses of **4** and **5** we simulated the top Emodel scored poses of each compound for 20 ns at least three times (Fig. 5 and Supplementary Fig. S4). All simulations showed that the accommodation of the bulky substituents towards the extracellular vestibule resulted in greater distances particularly in the lipophilic extracellular gate (Y133 and F293) compared to the ATPCA wt simulations (Supplementary Fig. S5). The carboxyl group of **4** adopted an axial conformation in three out of four simulations, whereas the carboxyl group of **5** adopted an axial conformation in all three simulations, confirming that conformational flexibility is important for accommodating increasing bulk while maintaining the coordination of the sodium ion (Supplementary Fig. S6). Similarly to ATPCA, the carboxyl group of **4** showed additional interactions with G57 and Y133, whereas the carboxyl group of **5** only interacted with G57. In contrast to ATPCA, the guanidine group of **4** solely forms hydrogen bonds with the backbone of F293 and only shows long term-interaction with E52 in the single simulation where the carboxyl group adopts an equatorial conformation (Supplementary Fig. S4). The guanidine group of **5**, however, did show hydrogen bonding with the BGT1 specific residues Q299 and E52 as well as with Y133 in all simulations which could explain why **5** is more potent and more selective than **4** (Supplementary Fig. S4). We hypothesize that the increased bulk of **5** stabilizes the plane of the ATPCA scaffold in a more favorable angle to undergo interactions with Q299 and E52 compared to **4**. According to this binding hypothesis, **6** and **7** are sterically too demanding to fit into the extracellular vestibule and therefore do not show activity.

### Validation of the proposed molecular interaction pattern by mutational experiments

Overall, the docking studies and MD simulations showed that the residues Q299 and E52 in hBGT1 can undergo key interactions with the guanidine moiety of ATPCA and its analogs. As Q299 and E52 are not conserved among the GATs, we further propose these residues as molecular determinants for the hBGT1-selectivity of these compounds. To evaluate the importance of these residues for activity and selectivity, mutagenesis experiments were performed, and **2**, **3**, **4**, **11**, and *l-15a* were evaluated in [^3^H]GABA uptake assays. Therefore, Q299 and E52 in hBGT1 were mutated to either alanine or the corresponding residues in the other hGAT subtypes (hBGT1 Q299L, E52A, E52Y, E52A + Q299L, and E52Y + Q299L). According to our binding hypothesis, **2**, **3**, **11**, and *l-15a* were predicted to show decreased activity at any of the mutants, whereas no change in activity was expected for **4** due to the adoption of a slightly different binding mode where Q299 and E52 are not relevant. In contrast, glutamine was introduced in the corresponding position L314Q in GAT3 and predicted to introduce activity for **2**, **3**, **11**, and *l-15a*, but not for **4**.

Table 2 summarizes the effects of the mutations in hBGT1 and hGAT3 on the potencies of GABA, ATPCA, **2**, **3**, **4, 11**, and *l-15a*. The hBGT1 E52Y + Q299L double mutant was proven not functional, as evident from the lack of [^3^H]GABA uptake (data not shown), while the other hBGT/hGAT3 mutants were functional, albeit with a lower total uptake compared to their wildtype (wt) counterparts (cf. CPM values in the caption of Table 2, for more details on the cell surface expression of the mutants see Supplementary Fig. S7). Furthermore, we consistently noticed a small decrease in the inhibitory potency of the tested compounds using transient GAT expression in tsA201 cells (Table 2) versus stable expression in CHO cells (Table 1)^36^. Generally, the inhibitory activity of GABA was increased at all hBGT1 mutants except for E52A which comprises the only “unnatural” mutation not related to any of the neurotransmitter transporters. This supports the notion that by making hBGT1 more similar to hGAT1, GABA activity will be increased due to naturally higher GABA activity at hGAT^58^. Interestingly, ATPCA and **3** did not show the predicted decrease of inhibitory activity at any of the single-point hBGT1 mutants, i.e. Q299L, E52Y, and E52A, compared to wt hBGT1, whereas **2** only showed decreased activity at the Q299L mutant. Yet, the activity of ATPCA and **3** were both decreased at the double mutant E52A + Q299L (see Table 2). On the other hand, the activities of **11** and *l-15a* could be decreased at the single-mutants hBGT1 Q299L and E52Y, and at the double mutant E52A + Q299L. Noteworthy, a trend for increased inhibitory activity was observed for the E52A mutant over all compounds except for compound **11**. Introducing the hBGT1-specific Q299 in the corresponding hGAT3 position, L314Q^44,50^, allowed the introduction of activity for ATPCA and to some extent for **2**, which strongly supports the notion that Q299 is relevant for activity and selectivity. Intriguingly, the same mutant failed to restore the activity of **3**, **11**, and *l-15a*. The analysis of the [^3^H]GABA uptake assay results of **4** showed that the activity was increased at all single hBGT1 mutants, but unaltered at the hBGT1 double mutant. This is in agreement with our simulations of **4** where its carboxyl group adopts an axial conformation and no long-term interactions with Q299 and E52 are formed, which renders these residues not relevant for binding. Accordingly, **4** also displayed decreased activity at the GAT3 L314Q mutant compared to wt hGAT3 underlining once more a different binding mode not involving Q299L.

**Table 2:** Effect of mutations in hBGT1 and hGAT3 on the inhibitory activities of GABA, ATPCA, and selected analogs.

**IC**_**50**_ (pIC_50_ ± S.E.M.) (µM)							
	BGT1					GAT3	
	wt	Q299L	E52Y	E52A	E52A + Q299L	wt	L314Q
**GABA**	**18** (4.74 ± 0.03)	**1.4** (5.87 ± 0.02)****	**1.2** (5.92 ± 0.05)****	**25** (4.60 ± 0.03)ns	**1.0** (6.01 ± 0.08)****	**8.2** (5.10 ± 0.02)	**5.2** (5.29 ± 0.04)*
**ATPCA**	**11** (4.98 ± 0.06)	**13** (5.89 ± 0.10)ns	**5.8** (5.24 ± 0.06)ns	**4.4** (5.36 ± 0.04)**	**36** (4.52 ± 0.15)*	**851** (3.23 ± 0.14)	**66** (4.23 ± 0.10)***
**2**	**52** (4.29 ± 0.03)	**108** (3.98 ± 0.07)*	**44** (4.43 ± 0.13)ns	**31** (4.52 ± 0.02)**	**133** (3.96 ± 0.14)ns	** > 1,000**	**695** (3.16 ± 0.11)
**3**	**406** (3.40 ± 0.05)	**203** (3.72 ± 0.10)ns	**152** (3.82 ± 0.05)**	**74** (4.14 ± 0.05)***	** > 1,000**	** > 1,000**	** > 1,000**
**4**	**354** (3.49 ± 0.09)	**157** (3.85 ± 0.09)*	**54** (4.27 ± 0.07)***	**90** (4.06 ± 0.07**)	**317** (3.60 ± 0.10)ns	**122** (3.94 ± 0.10)	**500** (3.31 ± 0.05)**
**11**	**220** (3.66 ± 0.05)	** > 1,000**	** > 1,000**	**247** (3.62 ± 0.06)ns	** > 1,000**	** > 1,000**	** > 1,000**
*l-15a*	**21** (4.68 ± 0.02)	** > 1,000**	** > 1,000**	**37** (4.43 ± 0.02)***	** > 1,000**	** > 1,000**	** > 1,000**

The compounds were examined for their ability to inhibit the uptake of 30 nM [^3^H]GABA at wildtype (wt) hBGT1 or hGAT3, or at mutated transporters transiently expressed in tsA201 cells. At least three independent experiments in triplicates were performed. Total and non-specific [^3^H]GABA uptake gave the following approximate CPM values, respectively: 4,000 and 60 (hBGT1 wt); 700 and 55 (Q299L); 600 and 60 (E52Y); 7,500 and 120 (E52A); 450 and 50 (E52A + Q299L); 8,500 and 70 (hGAT3 wt), 3,500 and 75 (L314Q). The pIC_50_ values at the mutants were compared to the wt transporter (unpaired Student’s t-test, not significant (ns), *P* > 0.05, *P < 0.05, **P < 0.01, ***P < 0.001, ****P < 0.0001). Lowered [^3^H]GABA uptake by the mutants hBGT1 Q299L, E52A + Q299L, and E52Y + Q299L was correlated with lower cell surface expression levels determined by ELISA (see Figure S7). E52A and E52Y showed similar surface expression levels as hBGT1 which suggests that the observed decreased [^3^H]GABA uptake is a result of impaired transport properties. Concentration response curves for all compounds can be found in the Supplementary Fig. S23.

Most interestingly, our model showed notable inconsistencies regarding the predicted activities of ATPCA, **2** and **3** versus **11** and *l-15a* at the mutants. While **11** and *l-15a* display the predicted loss in inhibitory activity at the hBGT1 mutants (except for E52A), we failed to restore activity in the hGAT3 Q314 mutant. In contrast, ATPCA and **3** only showed decreased activity at the hBGT1 double mutant, whereas **2** showed only decreased activity at the hBGT1 Q299L mutant. Yet, for ATPCA and **2** we successfully restore activity at the hGAT3 mutant, L314Q. In view of the structural resemblance of ATPCA and **11** and their similar molecular interactions predicted by the MD-refined docking poses (Fig. 4), we consider these different inhibition profiles particularly striking. We therefore conclude that GAT activity and selectivity is more complex than the potential interaction with E52 and Q299 and that flexibility, constituting the only structural difference between ATPCA and **11**, could account for the observed different inhibition profiles. Compared to ATPCA, **11** contains an additional double bond between *C*4 and *C*5 (Fig. 1), which renders the structure more rigid. Hence, the seemingly flexible molecules of ATPCA, **2**, and **3** could have adopted new conformations, such as the equatorial to axial shift of the carboxyl group already observed for **4** and **5,** which possibly allows to compensate to some extent the loss of interactions at the hBGT1 mutants. Analogously, the highly flexible GABA molecule has been suggested to adopt an extended or a “cyclic” conformation in the pocket of GATs^59^. Accordingly, the same flexibility could also account for reintroducing activity of ATPCA and **2** at the hGAT3 mutant L314Q, whereas **3**, **11** and *l-15a* remained inactive. *l-15a* displays a similar inhibitory profile compared to **11** at the mutants even though *l-15a* is lacking the additional double bond in the common scaffold. We hypothesize that *l-15a* is less flexible in the pocket due to the 4-methyl moiety that shows a stereospecific activity profile probably due to steric hindrance (Table 2). Nevertheless, we also need to address the possibility that our homology model is inaccurate, which is a general concern for any model built on a template with low sequence identity. However, previous mutational studies at hBGT1 have demonstrated that hBGT1 is remarkably resistant to mutations^60^. Consequently, we consider the presented mutational data as rather successfully in light of the challenging target.

**Figure 6:**
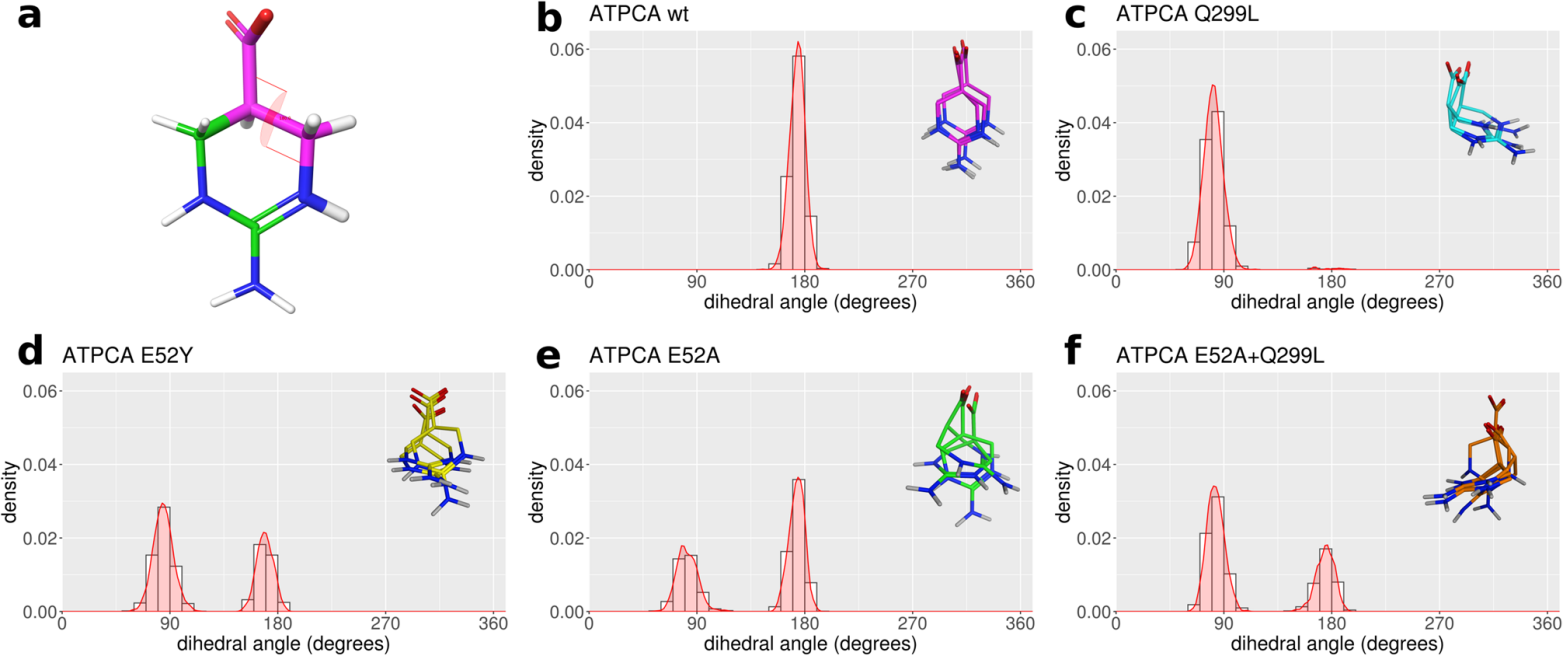
(**a**) Illustration of the measured dihedral angle between the carboxyl group and the tetrahydropyrimidine ring of ATPCA. (**d**–**f**) Density distributions of dihedral angles between the carboxyl group and the tetrahydropyrimidine ring of ATPCA in simulations of ATPCA in the wt hbGT1 and all mutants (bin width corresponds to ten degrees). Every tenth snapshot of every trajectory including all replicas were extracted (in total 1,251 snapshots per mutant) and the dihedral angles were calculated with vmd^70^. In contrast to ATPCA in wt hBGT1, the carboxyl group can adopt equatorial conformations (dihedral angle of ~ 90°) in all mutants.

To examine the postulated link between the conformational flexibility of the compounds and the activity observed at the mutants, we performed MD simulations of ATPCA, **4** and **11** at the hBGT1 mutants (Supplementary Figs. S8–S19). The best-scored poses of each of the three compounds were mutated in silico and simulated for 20 ns^61,62^. The trajectories were hierarchically clustered according to the ligand, and the interaction patterns were analyzed. Although no additional compensatory interactions were observed for ATPCA, we noted that ATPCA’s carboxyl group shifted from an exclusively equatorial conformation in wt hBGT1 to adopt also axial conformations in all hBGT1 mutants (Fig. 6a–f). These conformations are similar to the conformations that allow **4** and **5** to accommodate their bulky substituents in the extracellular vestibule. Furthermore, we observed that the carboxyl group of **4** also adopted this axial conformation throughout all simulations of the mutants (Supplementary Fig. S6). Conversely, MD simulations of **11** showed a flat structure in all hBGT1 mutants similar to the wt transporter (Supplementary Fig. S20). The MD simulations therefore support the notion that ATPCA can potentially adopt additional conformations in the hBGT1 mutants, similarly to **4,** which might be implicated in the observed compensatory mechanism in the hBGT1 mutants.

To further support this notion, we synthesized two analogs of **4**, where **9** contains an additional double bond in the scaffold between *C*5 and *C*6 and **8** contains a 4,4-bis(3-methyl-2-thienyl)3-butenyl substituent at *N*_*1*_ which corresponds to the substituents of the dual GAT1/BGT1 inhibitor EF1502^29^ (Figs. 1, 2a). According to our hypothesis, we predicted **9** to be inactive at wt hBGT1 due to the rigid scaffold which renders the compound incapable to adopt the axial conformation of the carboxyl group which our simulations and the mutational data suggested to be the most plausible binding conformation. In contrast, we predicted compound **8** to show at least some activity due to the flexible scaffold that allows the accommodation of the bulky *N*_3_-substituent in the extracellular vestibule. The two compounds were evaluated in [^3^H]GABA uptake assays at hBGT1 transiently expressed in tsA201 cells. Three independent experiments performed in triplicates confirmed that **9** showed no activity at hBGT1 wt (IC_50_ > 1,000 µM), whereas **8** showed some activity (IC_50_ 621uM, pIC_50_ 3.2 ± 0.03) (Table 1). These findings support our hypothesis that flexibility plays a crucial role for adopting the alternative binding hypothesis of **4** and **5** that allows the accommodation of bulky substituents as well as it potentially lays the ground for compensating the missing interactions for ATPCA, **2** and **3** at the mutants.

## Conclusion

Overall, our proposed binding mode can explain the SAR of all presented compounds in this study. Furthermore, our findings suggest that hBGT1 subtype selectivity of ATPCA and its analogs is complex as it is potentially governed not only by hydrogen bonding between the guanidine moiety and the residues E52 and Q299, but also by the flexibility of the compounds itself. Finally, our computational and experimental results suggest that the bulky compounds **4** and **5** adopt a slightly different binding mode than ATPCA and its small analogs, which possibly explains their decreased subtype selectivity. This binding mode can only be adopted due to a shift from the equatorial to an axial conformation of the carboxyl group of **4** and **5,** which allows the bulky *N*_*1*_ substituents to extend into the extracellular vestibule of hBGT1. Since this binding mode requires the opening of the external gate, it remains elusive how these compounds can be still transported. Similar conformational flexibility was also observed for ATPCA in MD simulations of the hBGT1 mutants Q299L, E52A, E52Y and E52A + Q299L. We therefore believe that this flexibility can to some extent compensate for the missing interactions of ATPCA, **2** and **3** in the hBGT1 mutants. Further confirmation was obtained by compound **9**, a rigid analog of **4,** which showed no activity at hBGT1. Overall, our experimentally validated binding hypothesis of a series of BGT1-selective substrate-inhibitors contributes to the understanding of GAT subtype selectivity and herein forms a versatile basis to guide the rational design of more potent BGT1 inhibitors.

## Experimental section

### Materials and compounds

Hank's Balanced Salt Solution (HBSS, no calcium, no magnesium no phenol red), Dulbecco's Phosphate Buffered Saline (DPBS), Ham’s F12 Nutrient Mix, Dulbecco's Modified Eagle Medium (DMEM) containing GlutaMAX-I, fetal bovine serum (FBS), penicillin–streptomycin (P/S), hygromycin B, and trypsin were purchased from Life Technologies (Paisley, UK). Plasmocin was purchased from InvivoGen (San Diego, CA, USA). PolyFect Transfection Reagent was purchased from Qiagen (West Sussex, UK). GABA, poly-d-lysine (PDL), and HEPES (4-(2-hydroxyethyl)piperazine-1-ethanesulfonic acid) were purchased from Sigma-Aldrich (St. Louis, MO, USA).[2,3-^3^H(*N*)]GABA (35.0 Ci/mmol).

### Plasmids

Epitope tagging of hBGT1 and hGAT3, subcloned into the pcDNA5/FRT vector^58^^,^ with an extracellularly located human influenza hemagglutinin (HA) peptide tag (YPYDVPDYA) has been previously described^60^. The mutated constructs, hBGT1 E52Y, E52A, C301S, Q299L + E52A, and Q299L + E52Y were synthetized by Genscript (Piscataway, NJ, USA), while the generation of the hBGT1 Q299L and hGAT3 L314Q mutants has been previously described^60^.

### Pharmacology

#### Cell culture and transfection

CHO Flp-In and tsA201 cells were cultured at 37 °C in a humidified atmosphere of 95% O_2_ and 5% CO_2_. As previously described^38,63^, CHO Flp-In cells stably expressing the hGATs were cultured in Ham’s F12 Nutrient Mix supplemented with 10% FBS, 1% P/S and plasmocin (5 µg/mL) under the selection pressure of hygromycin B (200 µg/mL). DMEM with GlutaMAX-I supplemented with 10% FBS and 1% P/S was used as growth medium for the tsA201 cells, which were transfected with DNA constructs using PolyFect according to the manufacturer’s specifications (Qiagen, West Sussex, UK), but with half of the recommended reagent volumes.

#### Radioligand-based uptake assays

Uptake assays were performed in PDL-coated white 96-well polystyrene cell culture microplates (PerkinElmer, Boston, MA, USA) as previously described^38,63^. Approximately 16–20 h after cell plating (~ 50.000 cells/well), cells were incubated with 30 nM of [^3^H]GABA with and without varying concentrations of ligands for 3 min at 37 °C. Assay buffer was HBSS supplemented with 20 mM HEPES, 1 mM CaCl_2_ and 1 mM MgCl_2_, pH 7.4. Non-specific [^3^H]GABA uptake was determined in the presence of 3 mM GABA. After termination, scintillation liquid (MicroScint-20, PerkinElmer) was added and the radioactivity measured in a TopCount NXT Microplate Scintillation Counter (PerkinElmer).

#### The FLIPR Membrane Potential (FMP) assay

The fluorescence-based FMP assay was performed as previously described^64^, for the functional characterization of the electrogenic transport of substrates via hBGT1. Briefly, CHO Flp-In cells stably expressing hBGT1 or hGAT1 were plated (~ 50,000 cells/well) into black PDL-coated 96-well plates with clear bottom (VWR, Radnor, PA, USA). The next day, the growth medium was removed, and cells were washed once with HBSS assay buffer and FMP Blue Dye (Molecular Devices, Crawley, UK), added, and incubated. Ligands were added via the NOVOStar plate reader (BMG Labtech GmbH, Offenburg, Germany), preheated to 37 °C, approximately 10 min before reading of the cell plate. The fluorescence signals were measured for 33.8 s upon ligand addition using an emission and excitation wavelength of 560 nm and 530 nm, respectively. Electrogenic transport of substrates via hBGT1 will lead to an influx of the negatively charged FMP Blue dye that will bind to intracellular proteins and lipids resulting in an increase in the fluorescence signal.

#### Enzyme-linked immunosorbent assay (ELISA)

ELISA was performed exactly as previously described using tsA201 cells transiently expressing HA-tagged or untagged hBGT1 constructs^65^. To this end, washed, fixated, blocked cells were incubated for 45 min with anti-HA (anti-HA.11, BioLegend, San Diego, CA, USA) diluted 1:1,000 in blocking solution, washed carefully and incubated for 45 min with a horseradish peroxidase-conjugated antibody (anti-mouse, P0447, Dako, Glostrup, Denmark) diluted 1:1,500 in blocking solution. Finally, quantification of the signal from well-washed cells were obtained using SuperSignal ELISA Femto substrate (ThermoFisher Scientific, Waltham, MA USA) by chemiluminescence in an EnSpire2300 Multilabel Reader (PerkinElmer). HA-tagged hBGT1 was used as a positive control, while untagged wt hBGT1 was used as a negative control. All experiments were performed in sextuplicate measurements in three independent experiments.

#### Data analysis

Data analysis and statistical evaluation were performed in GraphPad Prism 7.02 (GraphPad Software, San Diego, CA, USA). Concentration-uptake inhibition curves, generated from the radioligand-based uptake assays, were fitted by non-linear regression, using the equation for sigmoidal concentration–response with variable slope: Y = Bottom + (Top–Bottom)/(1 + 10^((logIC_50_ − X) × HillSlope)), where Y is the response, X is the logarithm of the concentration, Top and Bottom are the plateaus in same units as Y, logIC_50_ is the concentration giving a response halfway between Bottom and Top, and the HillSlope is the steepness of the curve. For the substrate concentration–response curves generated from the FMP assay, the same equation was used but with logEC_50_ instead for logIC_50_. Unpaired Student’s *t*-test was performed when appropriate, as indicated in the figure legends. Unless otherwise stated, data are presented as means ± S.E.M. of at least three independent pooled experiments performed in triplicates.

### Modeling

#### Model building

Homology models of hBGT1 in the outward-occluded state were (template: PDB code 2A65)^51^ used from the publication of Damgaard et al.^44^.

#### Docking

Induced fit docking was performed with the Schrödinger Suite 2015-2 using the OPLS2005 force field and extended sampling^45^. Ligands were drawn in ChemDraw 15.0.0.106, and ligand preparation was carried out with LigPrep^66^ using pH 7.4 ± 1. Protein preparation was done with PrepWiz^67^. Compounds with unknown absolute configuration of the carboxyl group (**2**–**5**, **11**–**14**, *l-15a,b*, and *u-15a,b*) were docked in *R* and *S* configurations. ATPCA shows a delocalized double bond within the guanidine moiety, thus, there is no stereo center at the carboxyl group. Due to the protonation of *N*_3_, the Schrödinger Suite automatically assigns different atom types to *N*_1_ and N_3_ which forces the user to define the stereocenter at *C*_1_. Since the OPLS2005 force field does not provide a suitable standard atom type that unites the characteristics of *N*_1_ and N_3_, we decided to dock ATPCA in an *R* and *S* configuration. 752 docking poses were generated and visualized with MOE 2016.08^68^. A hierarchical clustering of the poses was performed according to an in-house protocol for common scaffold clustering with a maximal distance of 3 Å within one cluster^48^. The poses were assembled into 33 clusters and the most populated cluster was considered as most promising. Within the cluster, poses with the highest Emodel score^49^ were selected for further MD refinement.

#### Molecular dynamics

MD simulations were performed with Desmond 4.2^61,62^ for 20 ns at least three times for each pose, using OPLS2005 force field, SPC solvent model, POPC (300 K) as a membrane model and 0.15 M NaCl salt concentration. The system was placed in a box with periodic boundary conditions and relaxed according to the standard protocol (NVT ensemble with short time steps with Brownian dynamics at 10 K and restrained solute heavy atoms, NVT ensemble using Berendsen thermostat, NPT ensemble using Berendsen thermostat and barostat). The production run was performed according to the standard protocol (NPT ensemble at 300 K, Berendsen thermostat and barostat, recording intervals of 1.2 ps for the energy and 4.8 ps for the trajectory). The trajectory was hierarchically clustered into 10 clusters according to the ligand with the python script *trajectory_clustering.py* available in the Schrödinger suite 2015-2^69^. For ATPCA, we simulated both the top-scored *R* and *S* configured poses (including one replica) to confirm that we observe the same interactions (see Fig. 4 and Fig. S3, data not shown for the *S* configuration).

### Chemistry

All synthetic procedures can be found in the Supplementary information page 25.

## Supplementary information

Supplementary file1

## Data Availability

The datasets generated and analysed during this study (the homology model, docking poses, simulation trajectories and code for analyzing the trajectories as well as code for generating figures) are available from the corresponding author upon request.

## References

[1] Mody I, Pearce RA (2004). Diversity of inhibitory neurotransmission through GABA(A) receptors. Trends Neurosci..

[2] Kowalczyk P, Kulig K (2014). GABA system as a target for new drugs. Curr. Med. Chem..

[3] Treiman DM (2001). GABAergic mechanisms in epilepsy. Epilepsia.

[4] Wafford K, Ebert B (2006). Gaboxadol: a new awakening in sleep. Curr. Opin. Pharmacol..

[5] Clarkson AN, Huang BS, MacIsaac SE, Mody I, Thomas Carmichael S (2010). Reducing excessive GABA-mediated tonic inhibition promotes functional recovery after stroke. Nature.

[6] Lie MEK, Ortega A, Schousboe A (2017). Glial GABA transporters as modulators of inhibitory signalling in epilepsy and stroke. Glial Amino Acid Transporters.

[7] Lie, M. E. *et al.* GAT3 selective substrate l-isoserine upregulates GAT3 expression and increases functional recovery after a focal ischemic stroke in mice. *J. Cereb. Blood Flow Metab.* 271678X17744123 (2017). 10.1177/0271678X17744123 PMC6311676 29160736

[8] Scimemi A (2014). Structure, function, and plasticity of GABA transporters. Front. Cell. Neurosci..

[9] Alexander SP (2015). The concise guide to pharmacology 2015/16: transporters. Br. J. Pharmacol..

[10] Conti F, Minelli A, Melone M (2004). GABA transporters in the mammalian cerebral cortex: localization, development and pathological implications. Brain Res. Brain Res. Rev..

[11] Melone M, Barbaresi P, Fattorini G, Conti F (2005). Neuronal localization of the GABA transporter GAT-3 in human cerebral cortex: a procedural artifact?. J. Chem. Neuroanat..

[12] Conti F (1999). Neuronal, glial, and epithelial localization of gamma-aminobutyric acid transporter 2, a high-affinity gamma-aminobutyric acid plasma membrane transporter, in the cerebral cortex and neighboring structures. J. Comp. Neurol..

[13] Zhou Y (2012). The betaine-GABA transporter (BGT1, slc6a12) is predominantly expressed in the liver and at lower levels in the kidneys and at the brain surface. Am. J. Physiol. Renal Physiol..

[14] Schousboe A, Wellendorph P, Frølund B, Clausen RP, Krogsgaard-Larsen P (2017). Astrocytic GABA transporters: pharmacological properties and targets for antiepileptic drugs. Adv. Neurobiol..

[15] Dalby NO (2000). GABA-level increasing and anticonvulsant effects of three different GABA uptake inhibitors. Neuropharmacology.

[16] Olsen RW, Sieghart W (2008). International Union of Pharmacology. LXX. Subtypes of gamma-aminobutyric acid(A) receptors: classification on the basis of subunit composition, pharmacology, and function. Update. Pharmacol. Rev..

[17] Bettler B, Tiao JY-H (2006). Molecular diversity, trafficking and subcellular localization of GABAB receptors. Pharmacol. Ther..

[18] Schousboe A, Sarup A, Larsson OM, White HS (2004). GABA transporters as drug targets for modulation of GABAergic activity. Biochem. Pharmacol..

[19] Madsen KK (2009). Synaptic and extrasynaptic GABA transporters as targets for anti-epileptic drugs. J. Neurochem..

[20] Damgaard M, Ortega A, Schousboe A (2017). Development of non-GAT1-selective inhibitors: challenges and achievements. Glial Amino Acid Transporters.

[21] Łątka, K., Jończyk, J. & Bajda, M. γ-Aminobutyric acid transporters as relevant biological target: their function, structure, inhibitors and role in the therapy of different diseases. *Int. J. Biol. Macromol.* **158**, 750–772 (2020). 10.1016/j.ijbiomac.2020.04.126 32360967

[22] Zaręba, P., Gryzło, B., Mazur, G. & Malawska, B. Development, recent achievements and current directions of research into GABA uptake inhibitors. *Curr. Med. Chem.*10.2174/0929867325666191010120236 (2019). 10.2174/0929867325666191010120236 31612818

[23] Leach JP, Brodie MJ (1998). Tiagabine. Lancet.

[24] Borden LA (1994). Tiagabine, SK&F 89976-A, CI-966, and NNC-711 are selective for the cloned GABA transporter GAT-1. Eur. J. Pharmacol..

[25] Chiu C-S (2005). GABA transporter deficiency causes tremor, ataxia, nervousness, and increased GABA-induced tonic conductance in cerebellum. J. Neurosci..

[26] Schousboe A, Madsen KK, White HS (2011). GABA transport inhibitors and seizure protection: the past and future. Future Med. Chem..

[27] Kälviäinen R (2001). Long-term safety of tiagabine. Epilepsia.

[28] Madsen KK, White HS, Schousboe A (2010). Neuronal and non-neuronal GABA transporters as targets for antiepileptic drugs. Pharmacol. Ther..

[29] Clausen RP (2005). Selective inhibitors of GABA uptake: synthesis and molecular pharmacology of 4-N-methylamino-4,5,6,7-tetrahydrobenzo[d]isoxazol-3-ol analogues. Bioorg. Med. Chem..

[30] Vogensen SB (2013). Selective mGAT2 (BGT-1) GABA uptake inhibitors: design, synthesis, and pharmacological characterization. J. Med. Chem..

[31] White HS (2005). First demonstration of a functional role for central nervous system betaine/γ-aminobutyric acid transporter (mGAT2) based on synergistic anticonvulsant action among inhibitors of mGAT1 and mGAT2. J. Pharmacol. Exp. Ther..

[32] Madsen KK (2011). Selective GABA transporter inhibitors tiagabine and EF1502 exhibit mechanistic differences in their ability to modulate the ataxia and anticonvulsant action of the extrasynaptic GABA(A) receptor agonist gaboxadol. J. Pharmacol. Exp. Ther..

[33] Smith MD (2008). Inhibition of the betaine-GABA transporter (mGAT2/BGT-1) modulates spontaneous electrographic bursting in the medial entorhinal cortex (mEC). Epilepsy Res..

[34] Kobayashi T (2014). Conformationally restricted GABA with Bicyclo[3.1.0]hexane backbone as the first highly selective BGT-1 inhibitor. ACS Med. Chem. Lett..

[35] Lehre AC (2011). Deletion of the betaine–GABA transporter (BGT1; slc6a12) gene does not affect seizure thresholds of adult mice. Epilepsy Res..

[36] Borden LA (1996). GABA transporter heterogeneity: pharmacology and cellular localization. Neurochem. Int..

[37] Kickinger S (2019). Structural and molecular aspects of betaine-GABA transporter 1 (BGT1) and its relation to brain function. Neuropharmacology.

[38] Al-Khawaja A (2014). Pharmacological identification of a guanidine-containing β-alanine analogue with low micromolar potency and selectivity for the betaine/GABA transporter 1 (BGT1). Neurochem. Res..

[39] Atwal KS (1992). Dihydropyrimidine angiotensin II receptor antagonists. J. Med. Chem..

[40] Csuk R (2012). Isolation, structure, synthesis and cytotoxicity of an unprecedented flupirtine dimer. Zeitschrift für Naturforschung B.

[41] Baskaran, S., Hanan, E., Byun, D. & Shen, W. A facile reduction of 2-aminopyrimidines with triethylsilane and trifluoroacetic acid. *ChemInform.* **35**, 2107–2111 (2004).

[42] Braestrup C (1990). (R)-N-[4,4-bis(3-methyl-2-thienyl)but-3-en-1-yl]nipecotic acid binds with high affinity to the brain gamma-aminobutyric acid uptake carrier. J. Neurochem..

[43] Jensen AA, Bräuner-Osborne H (2004). Pharmacological characterization of human excitatory amino acid transporters EAAT1, EAAT2 and EAAT3 in a fluorescence-based membrane potential assay. Biochem. Pharmacol..

[44] Damgaard M (2015). Identification of the first highly subtype-selective inhibitor of human GABA transporter GAT3. ACS Chem. Neurosci..

[45] Schrödinger Release 2015: Schrödinger Suite 2015-2 Induced Fit Docking protocol; Glide 6.7, Schrödinger, LLC, New York, NY, 2015; Prime 4.0, Schrödinger, LLC, New York, NY, 2015.

[46] Sarker S (2010). The high-affinity binding site for tricyclic antidepressants resides in the outer vestibule of the serotonin transporter. Mol. Pharmacol..

[47] Klepsch F, Ecker GF (2010). Impact of the recent mouse P-glycoprotein structure for structure-based ligand design. Mol. Inform..

[48] Richter L (2012). Diazepam-bound GABAA receptor models identify new benzodiazepine binding-site ligands. Nat. Chem. Biol..

[49] Friesner RA (2004). Glide: a new approach for rapid, accurate docking and scoring. 1. Method and assessment of docking accuracy. J. Med. Chem..

[50] Beuming T, Shi L, Javitch JA, Weinstein H (2006). A comprehensive structure-based alignment of prokaryotic and eukaryotic neurotransmitter/Na symporters (NSS) aids in the use of the LeuT structure to probe NSS structure and function. Mol. Pharmacol..

[51] Yamashita A, Singh SK, Kawate T, Jin Y, Gouaux E (2005). Crystal structure of a bacterial homologue of Na^+^/Cl^−^–dependent neurotransmitter transporters. Nature.

[52] LeVine MV (2019). The allosteric mechanism of substrate-specific transport in SLC6 is mediated by a volumetric sensor. Proc. Natl. Acad. Sci. U.S.A..

[53] Melamed N, Kanner BI (2004). Transmembrane domains I and II of the gamma-aminobutyric acid transporter GAT-4 contain molecular determinants of substrate specificity. Mol. Pharmacol..

[54] Yamashita A, Singh SK, Kawate T, Jin Y, Gouaux E (2005). Crystal structure of a bacterial homologue of Na^+^/Cl^−^–dependent neurotransmitter transporters. Nature.

[55] Forrest LR, Rudnick G (2009). The rocking bundle: a mechanism for ion-coupled solute flux by symmetrical transporters. Physiology.

[56] Loland CJ (2015). The use of LeuT as a model in elucidating binding sites for substrates and inhibitors in neurotransmitter transporters. Biochim. Biophys. Acta (BBA) Gen Subj..

[57] Kristensen AS (2011). SLC6 neurotransmitter transporters: structure, function, and regulation. Pharmacol. Rev..

[58] Kvist T, Christiansen B, Jensen A, Brauner-Osborne H (2009). The four human γ-aminobutyric acid (GABA) transporters: pharmacological characterization and validation of a highly efficient screening assay. Comb. Chem. High Throughput Screen..

[59] Kanner BI, Zomot E (2008). Sodium-coupled neurotransmitter transporters. Chem. Rev..

[60] Jørgensen L (2017). Structure–activity relationship, pharmacological characterization, and molecular modeling of noncompetitive inhibitors of the betaine/γ-aminobutyric acid transporter 1 (BGT1). J. Med. Chem..

[61] Desmond molecular dynamics system, version 4.2, D. E. Shaw Research, New York, NY, 2015. Maestro-desmond interoperability tools, version 4.2, Schrödinger, New York, NY, 2015.

[62] Bowers, K. J., Chow, D. E., Dror, R. O., Eastwood, M. P., Gregersen, B. A., Klepeis, J. L., Kolossvary, I., Moraes, M. A., Sacerdoti, F. D., Salmon, J. K., Shan, Y. & Shaw, D. E. Scalable algorithms for molecular dynamics simulations on commodity clusters. In *Proceedings of the 2006 ACM/IEEE Conference on Supercomputing* (2006).

[63] Kragholm B (2013). Discovery of a subtype selective inhibitor of the human betaine/GABA transporter 1 (BGT-1) with a non-competitive pharmacological profile. Biochem. Pharmacol..

[64] Christiansen B, Meinild A-K, Jensen AA, Braüner-Osborne H (2007). Cloning and characterization of a functional human gamma-aminobutyric acid (GABA) transporter, human GAT-2. J. Biol. Chem..

[65] Lie MEK (2019). GAT3 selective substrate l-isoserine upregulates GAT3 expression and increases functional recovery after a focal ischemic stroke in mice. J. Cereb. Blood Flow Metab..

[66] Schrödinger Release 2015-2: LigPrep, Schrödinger, LLC, New York, NY, 2015.

[67] Schrödinger Release 2017-2: Schrödinger Suite 2015-2 Protein Preparation Wizard; Epik, Schrödinger, LLC, New York, NY, 2016; Impact, Schrödinger, LLC, New York, NY, 2015; Prime, Schrödinger, LLC, New York, NY, 2015.

[68] Molecular Operating Environment (MOE), 2016.08, Chemical Computing Group Inc., 1010 Sherbooke St. West, Suite #910, Montreal, QC, Canada, H3A 2R7, 2016.

[69] Schrödinger Release 2015-2: Maestro, Schrödinger, LLC, New York, NY, 2015.

[70] Humphrey W, Dalke A, Schulten KVMD (1996). Visual molecular dynamics. J. Mol. Graph..

