# Supplementary material for: Exploring the molecular determinants for subtype-selectivity of 2-amino-1,4,5,6-tetrahydropyrimidine-5-carboxylic acid analogs as betaine/GABA transporter 1 (BGT1) substrate-inhibitors
Source: Sci Rep. 2020 Aug 3;10:12992. doi: 10.1038/s41598-020-69908-w (PMC7400577; doi:10.1038/s41598-020-69908-w)
Supplement: Supplementary file 1 — Supplementary file1 [file 41598_2020_69908_MOESM1_ESM.pdf]

Supporting Information

Exploring the molecular determinants for subtype-selectivity of 2-amino-1,4,5,6-tetrahydropyrimidine-5-carboxylic acid analogs as betaine/GABA transporter 1 (BGT1) substrate-inhibitors

Stefanie Kickinger[1], Anas Al-Khawaja[2], Anne Stæhr Haugaar[2], Maria E.K. Lie[2], Francesco Bavo[2], Rebekka Löffler[2], Maria Damgaard[2], Gerhard F. Ecker[1], Bente Frølund[2], and Petrine Wellendorph[2]

[1]University of Vienna, Department of Pharmaceutical Chemistry, Althanstrasse 14, 1090 Vienna, Austria

[2]University of Copenhagen, Department of Drug Design and Pharmacology, Faculty of Health and Medical Sciences, 2100 Copenhagen, Denmark

**Chemistry*. B.F.: phone +45 3533 6495, e-mail, bfr@sund.ku.dk

**Pharmacology*. P.W.: phone +45 3533 6397, e-mail pw@sund.ku.dk

**Modeling*. G.F.E.: phone +431 4277 55110, e-mail gerhard.f.ecker@univie.ac.at

TABLE OF CONTENT

Distribution of docking poses and compounds per cluster **S1**

Distribution of poses per compound in the most populated cluster... **S2**

Root-mean-square-deviation (RMSD) and protein-ligand-interaction diagrams of selected MD simulations of ATPCA and **11** in wildtype hBGT1.. **S3**

RMSD and protein-ligand-interaction diagrams of selected MD simulations of **4** and **5** in wildtype hBGT1... **S4**

Distances between the external gate residues of hBGT1 in the simulations of ATCPA, **4** and **5** in wt hBGT1.. **S5**

Illustration of the measured dihedral angle between the carboxyl group and the dihydropyrimidine ring of **4** and **5**.. **S6**

Cell surface expression levels of HA-tagged mutated hBGT1 constructs................................... **S7**

RMSD and protein-ligand-interaction diagrams of selected MD simulations of ATPCA , **4** and **11** in hBGT1 Q299L, E52Y, E52A, and Q299L+E52A.. **S8–19**

Distribution of dihedral angles in simulations of **11** in wt hBGT1 and all mutants................... **S20**

Concentration-response curves of selected compounds from Table 1....................................... **S21**

Concentration-response curves of **8** and **9** from Table 1 .... **S23**

Concentration-response curves of compounds from Table 2 .... **S22**

Synthetic procedures of ATPCA analogs .... **page 25**

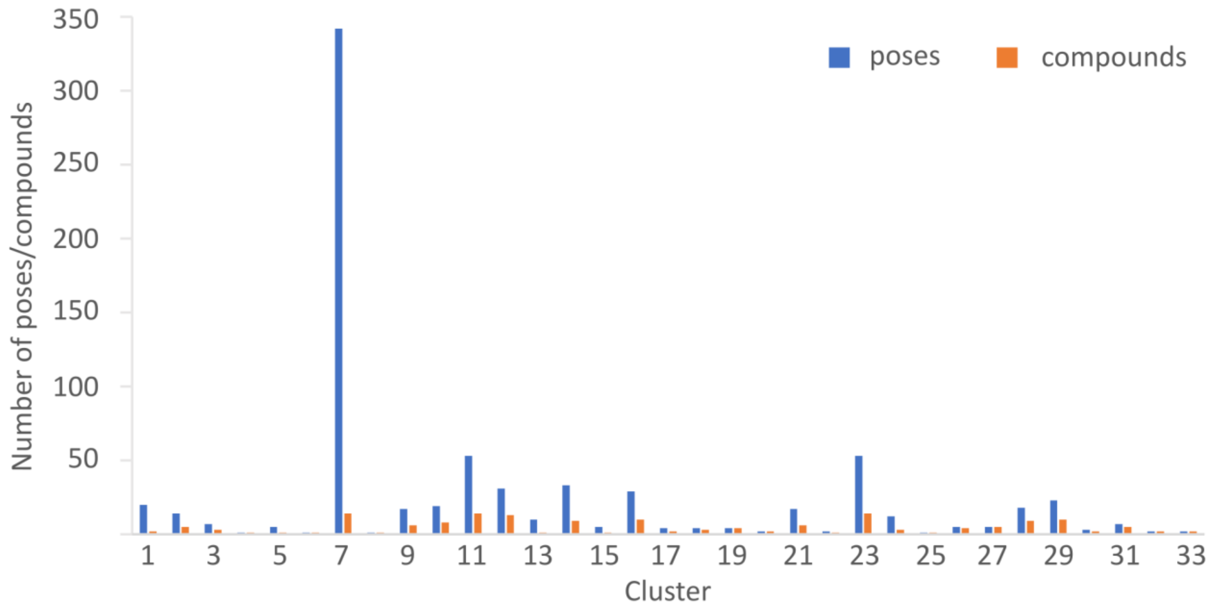

Figure S1. Number of total docking poses and number of different compounds per cluster (most clusters contain several poses per compound) according to hierarchical clustering of the common scaffold. The most populated cluster (7) contains 342 poses of all docked compounds, except for poses of **4** and **5**.

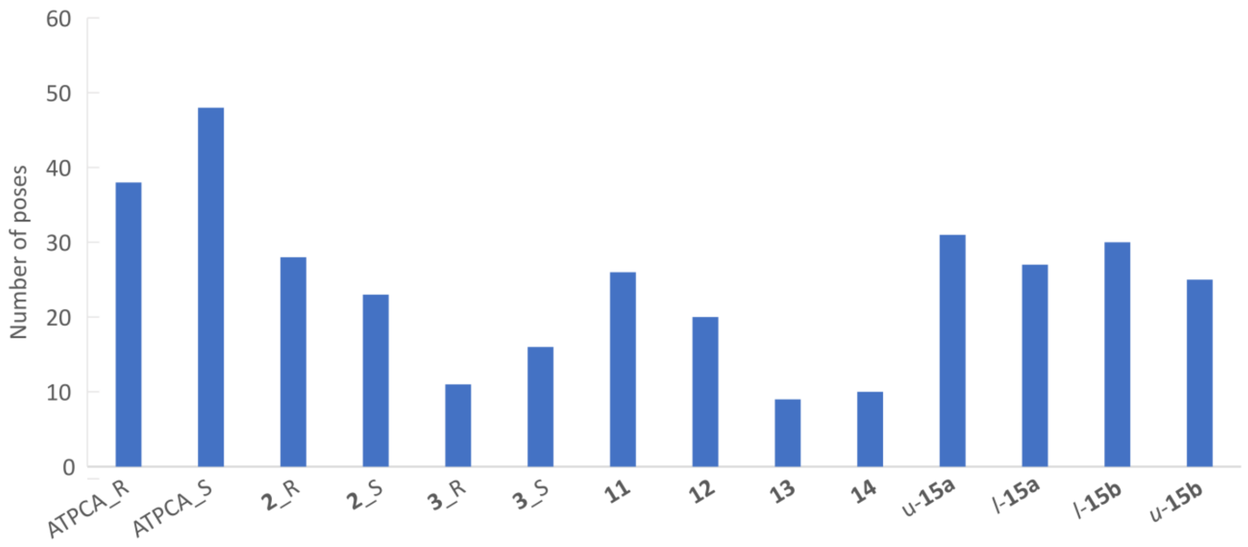

Figure S2. Distribution of poses per compound in the most populated cluster.

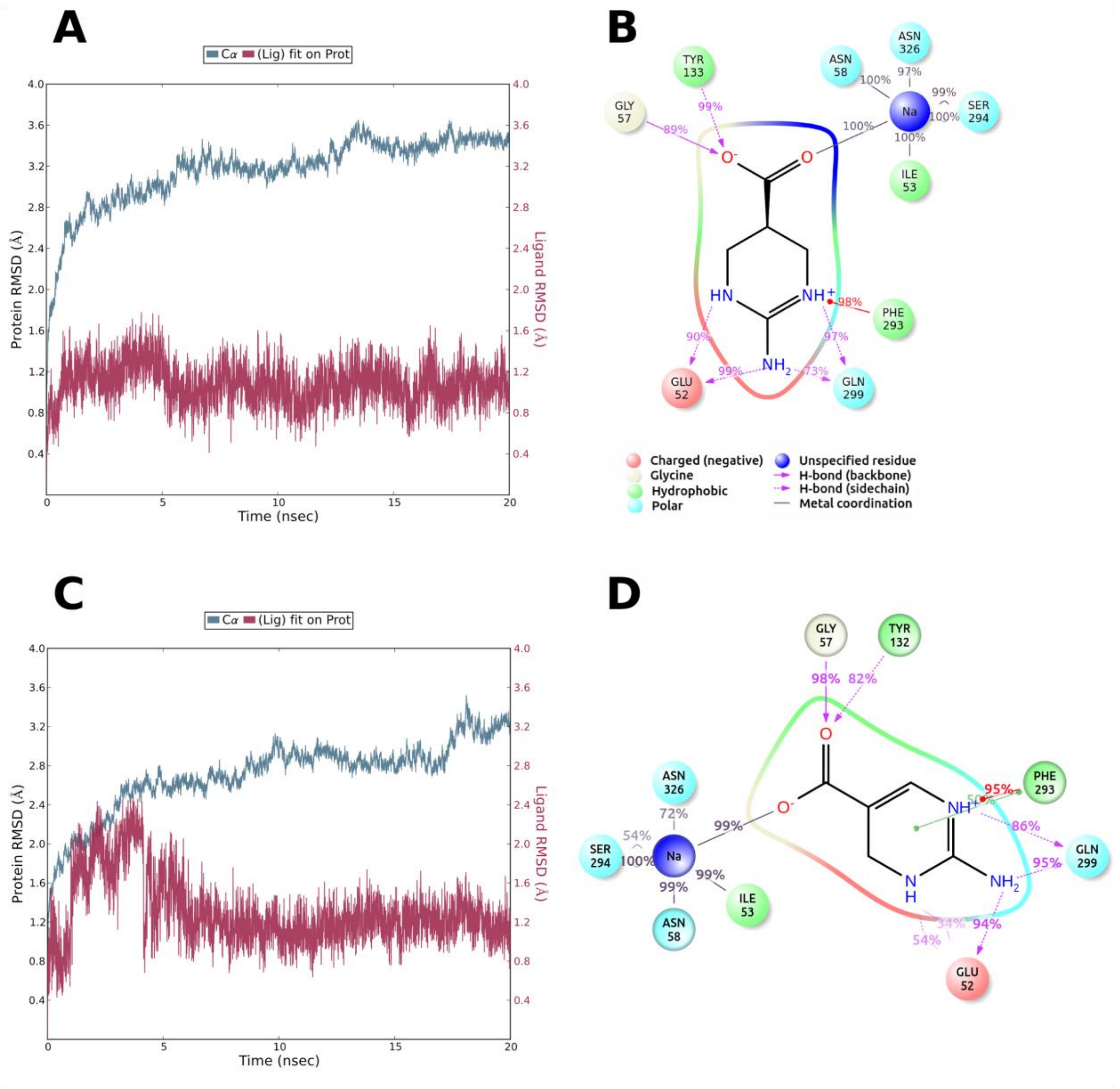

Figure S3. (A) Root-mean-square-deviation (RMSD) of one MD simulation run of the highest-scored ATPCA docking pose in wildtype hBGT1. **(B)** Protein-ligand-interaction schematic overview of the same MD simulation run as in A. Two additional replicas showed similar results. **(C)** RMSD of one MD simulation run of the highest-scored **11** docking pose in wildtype hBGT1. **(D)** Protein-ligand-interaction schematic overview of the same MD simulation run as in C.

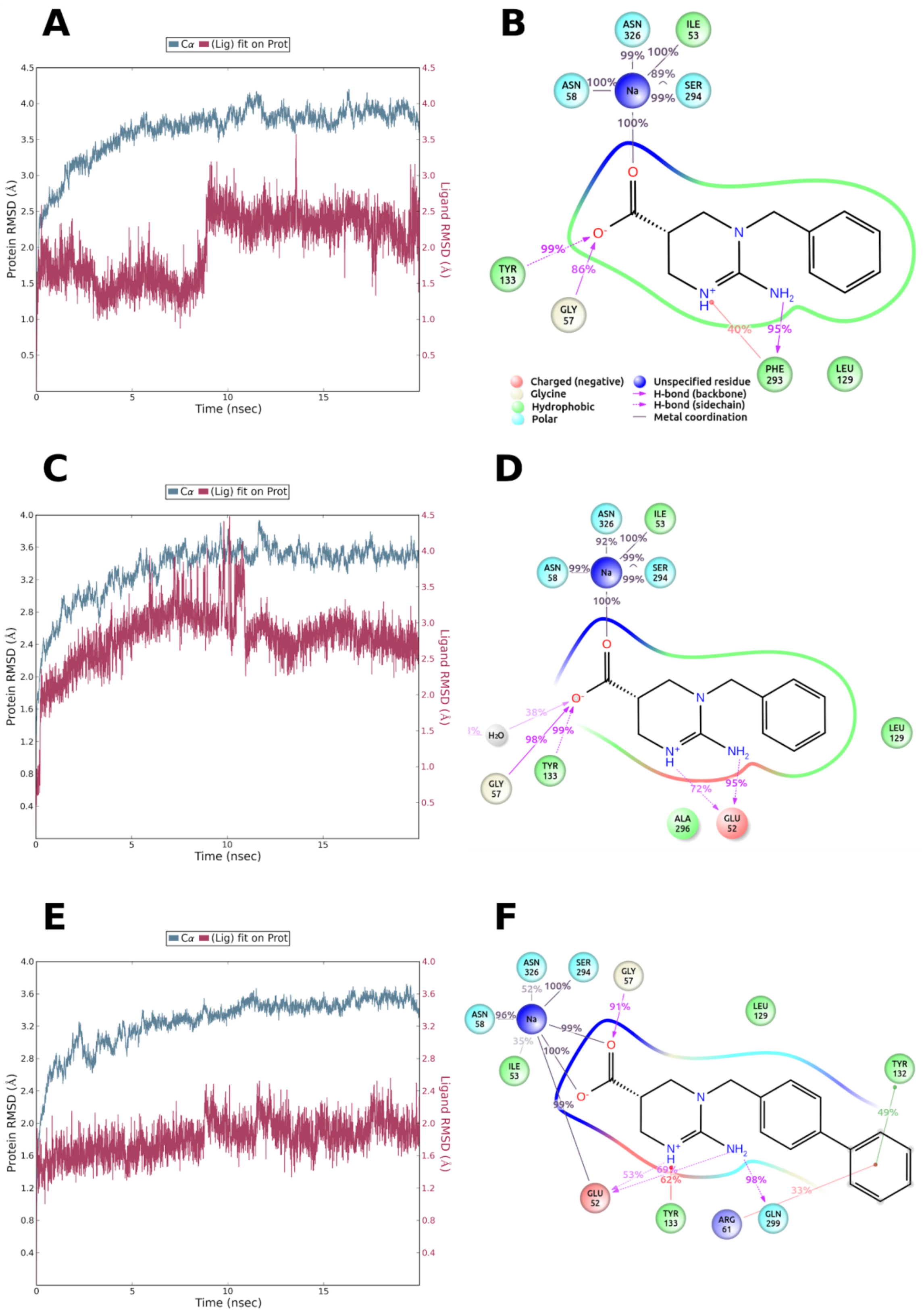

Figure S4. (A) Root-mean-square-deviation (RMSD) of one MD simulation run of the highest-scored **4** docking pose in wildtype hBGT1. **(B)** Protein-ligand-interaction schematic overview of

the same MD run as in A. Two additional replicas showed similar results. **(C)** RMSD of one MD simulation run of **4** in wildtype hBGT1 where the carboxyl group adopted an equatorial conformation. **(D)** Protein-ligand-interaction schematic overview of the same MD run as in C. **(E)** RMSD of one MD simulation run of the highest-scored **5** docking pose in wildtype hBGT1. **(F)** Protein-ligand-interaction schematic overview of the same MD run as in E. Two additional replicas showed similar results.

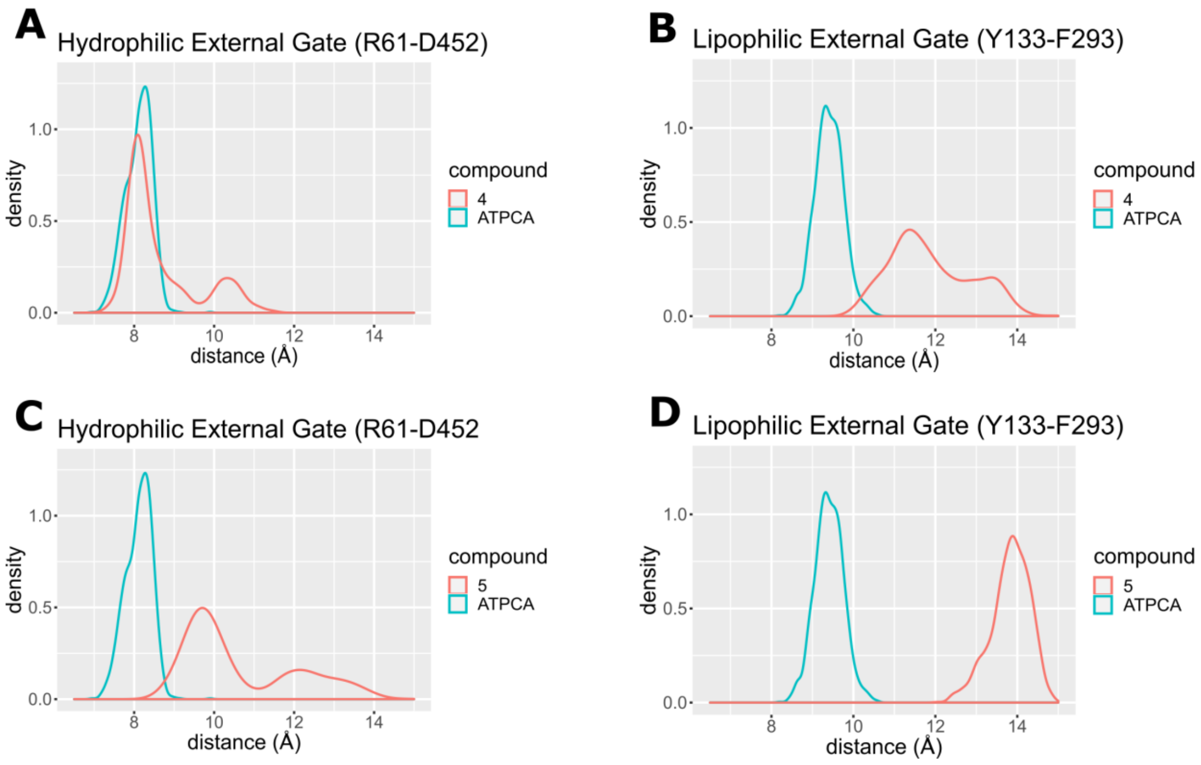

Figure S5. (A-D) Distances between the external gate residues of hBGT1 (hydrophilic gate R61 and D452, lipophilic gate Y133 and F293) in the simulations of ATCPA, **4** and **5** in wt hBGT1. Every tenth snapshot of the trajectories of ATPCA, **4** and **5** including all replicas were extracted (417 snapshots per simulation) and the distances based on the geometric center of each residue were calculated with vmd.[1]

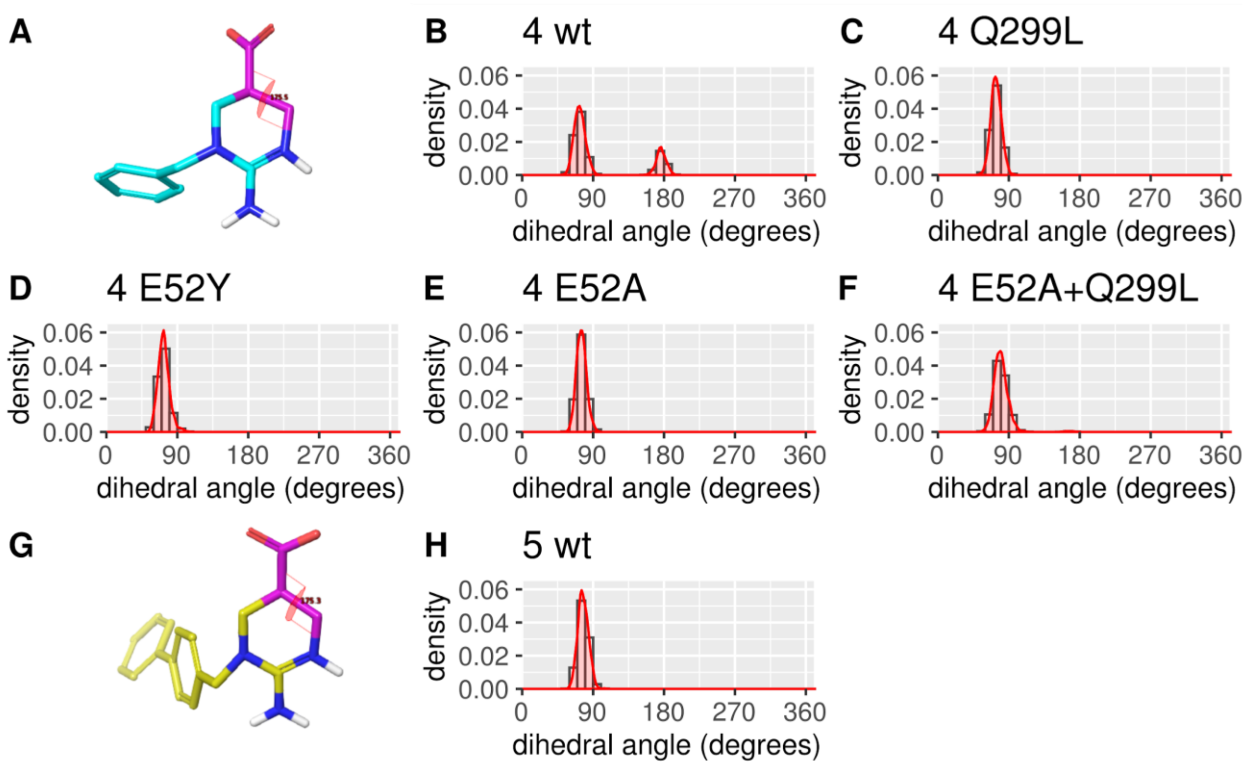

Figure S6. (A) Illustration of the measured dihedral angle between the carboxyl group and the dihydropyrimidine ring of **4**. **(B-F)** Density distributions of dihedral angles between the carboxyl group and the dihydropyrimidine ring of **4** in the simulations of **4** in wt hBGT1 and all mutants (bin width corresponds to ten degrees). Every tenth snapshot of every trajectory including all replicas were extracted (417 snapshots per simulation) and the dihedral angles were calculated with vmd.[1] **(G)** Illustration of the measured dihedral angle between the carboxyl group and the dihydropyrimidine ring of **5**. **(H)** Density distribution of dihedral angles between the carboxyl group and the dihydropyrimidine ring of **5** in the simulations of **5** in wt hBGT1. The dihedrals were calculated according to the same procedure as in B-F.

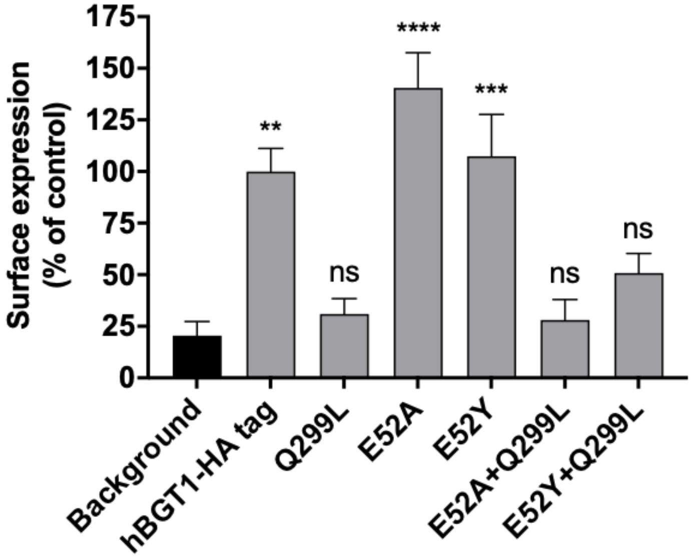

Figure S7. Cell surface expression levels of human influenza hemagglutinin (HA)-tagged mutated hBGT1 constructs. The constructs were transiently expressed in tsA201 cells, and the surface expression level was determined using ELISA with a primary anti-HA antibody and a horseradish peroxidase-conjugated secondary antibody. Untagged hBGT1 (wildtype) and HA-tagged hBGT1 (wildtype) were used as negative (background) and positive controls, respectively. Results are normalized and presented as means ± S.E.M. as percentage of the control from three independent experiments. The surface expression levels of hBGT1 (wildtype) and the mutants were compared to background (One-way ANOVA followed by Dunnett's multiple comparison test, not significant (ns) $P>0.05$, **$P<0.01$, ***$P<0.001$, ****$P<0.0001$). We detected a low surface expression of hBGT1 Q299L, E52A+Q299L, and E52Y+Q299L that was slightly, although not significantly, higher than the background. This suggested that the lack or diminished radioligand uptake seen by these transporters was related to a reduced surface expression. On the other hand, the surface expression levels of E52A and E52Y were comparable to the expression level of wt HA-hBGT1, which suggested that the reduced radioligand uptake seen by hBGT1 E52Y reflected impaired transport properties. Total [^{3}H]GABA uptake by the hGAT3 L314Q construct was lower compared to wt hGAT3 (cf. CPM values in the caption of Table 2). Nevertheless, a sufficiently large uptake window was achieved, and we did not further investigate whether the reduced uptake was related to a lower surface expression or a diminished transporter function.

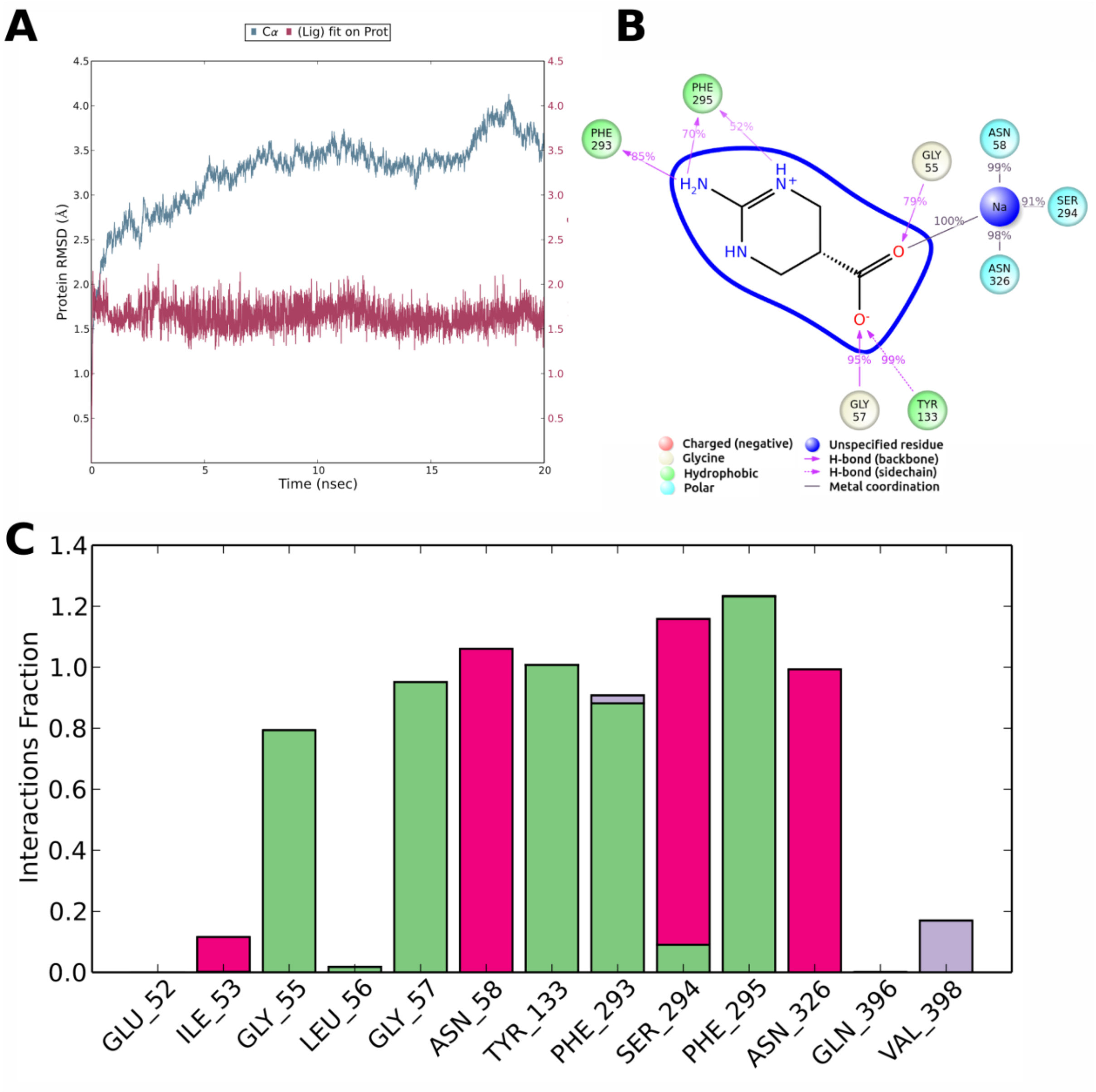

Figure S8. (A) RMSD of one MD simulation run of the highest-scored ATPCA docking pose in the hBGT1 Q299L mutant. **(B)** Protein-ligand-interaction schematic overview of the same MD simulation run as in A. Two MD replicas showed similar results. **(C)** Protein-ligand interaction diagram of the same MD simulation run as in A (pink, ionic interactions; green, hydrogen bonding; purple, hydrophobic contacts). An interaction fraction value of 1.0 indicates that this interaction is maintained 100% of the time during the simulation. Values bigger than 1.0 are possible due to multiple contacts of the ligand with the same residue.

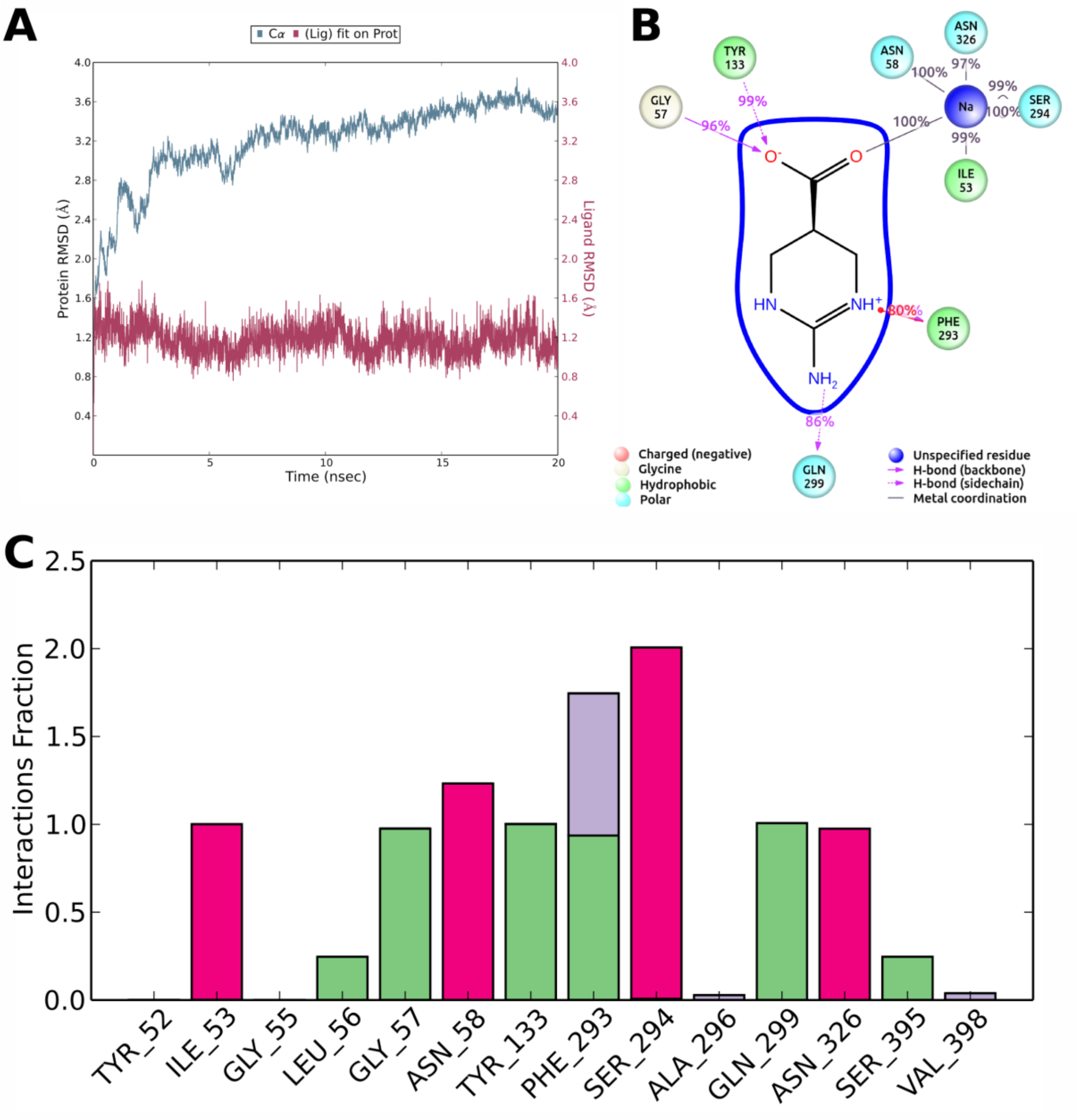

Figure S9. (A) RMSD of one MD simulation run of the highest-scored ATPCA docking pose in the hBGT1 E52Y mutant. **(B)** Protein-ligand-interaction schematic overview of the same MD simulation run as in A. Two MD replicas showed similar results. **(C)** Protein-ligand interaction diagram of the same MD simulation run as in A (pink, ionic interactions; green, hydrogen bonding; purple, hydrophobic contacts). An interaction fraction value of 1.0 indicates that this interaction is maintained 100% of the time during the simulation. Values bigger than 1.0 are possible due to multiple contacts of the ligand with the same residue.

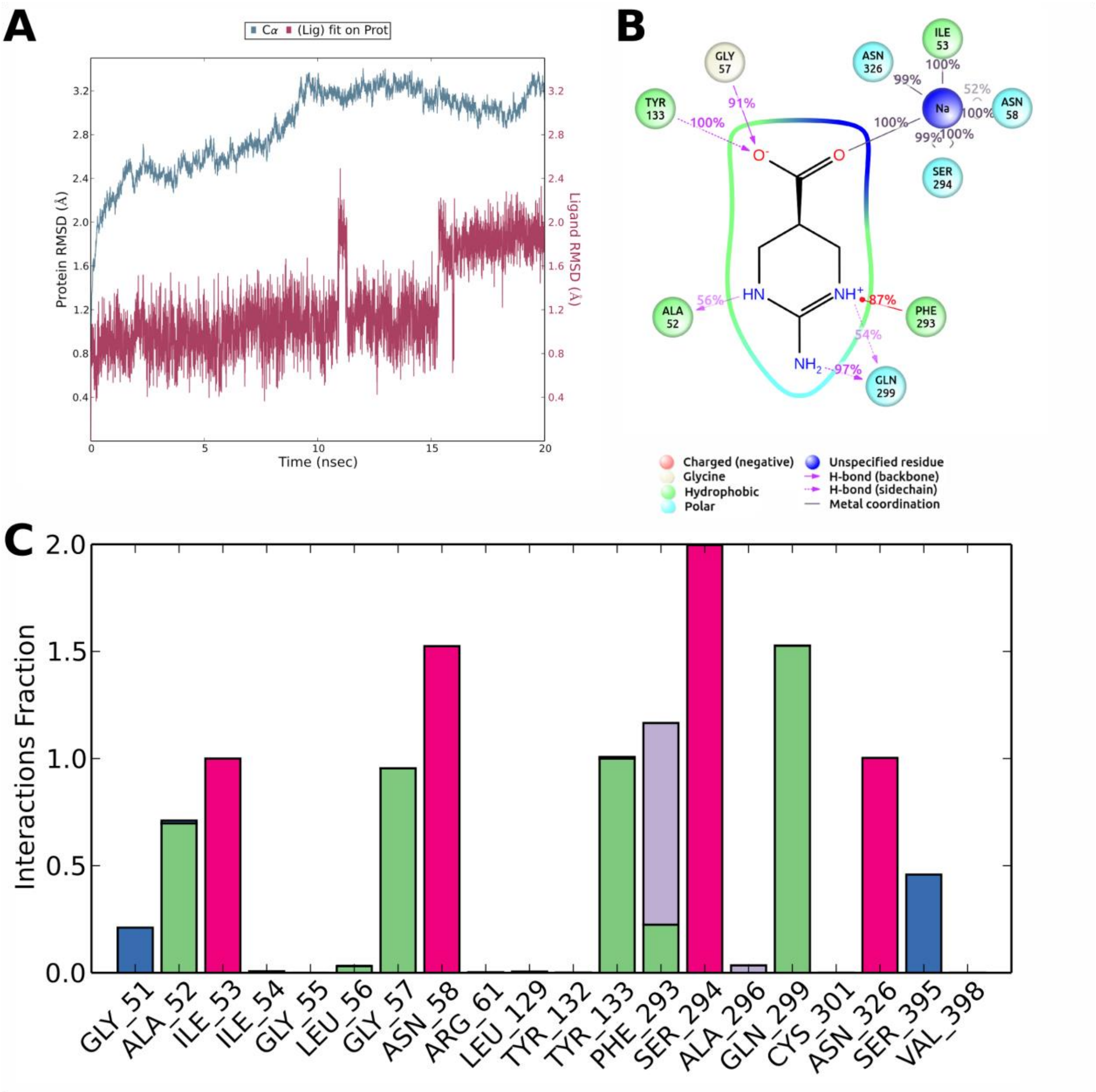

Figure S10. (A) RMSD of one MD simulation run of the highest-scored ATPCA docking pose in the hBGT1 E52A mutant. **(B)** Protein-ligand-interaction schematic overview of the same MD simulation run as in A. Two MD replicas showed similar results. **(C)** Protein-ligand interaction diagram of the same MD simulation run as in A (pink, ionic interactions; green, hydrogen bonding; purple, hydrophobic contacts; blue, water contacts). An interaction fraction value of 1.0 indicates that this interaction is maintained 100% of the time during the simulation. Values bigger than 1.0 are possible due to multiple contacts of the ligand with the same residue.

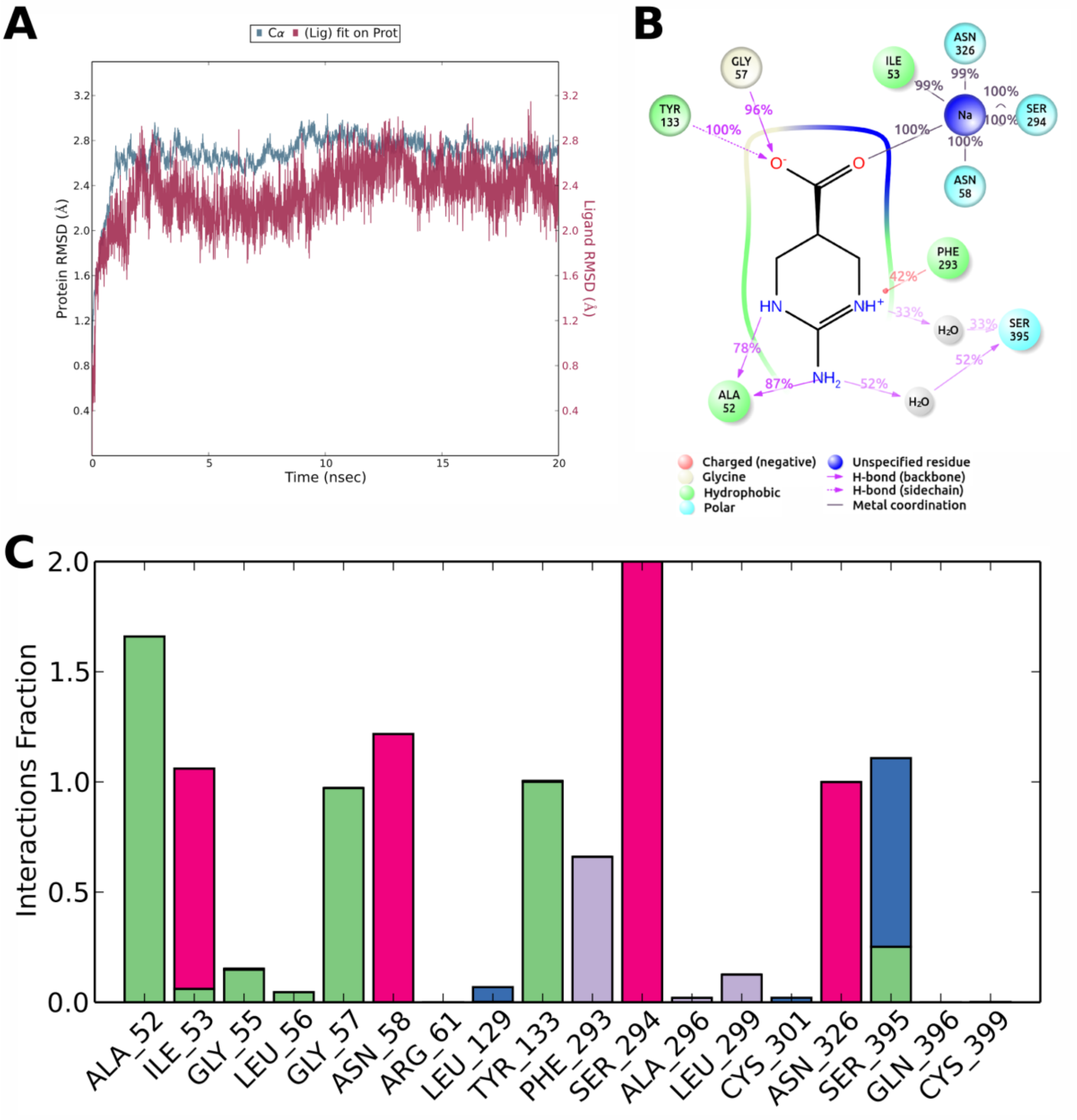

Figure S11. (A) RMSD of one MD simulation run of the highest-scored ATPCA docking pose in the hBGT1 E52A+Q299L mutant. **(B)** Protein-ligand-interaction schematic overview of the same MD simulation run as in A. Two MD replicas showed similar results. **(C)** Protein-ligand interaction diagram of the same MD simulation run as in A (pink, ionic interactions; green, hydrogen bonding; purple, hydrophobic contacts; blue, water contacts). An interaction fraction value of 1.0 indicates that this interaction is maintained 100% of the time during the simulation. Values bigger than 1.0 are possible due to multiple contacts of the ligand with the same residue.

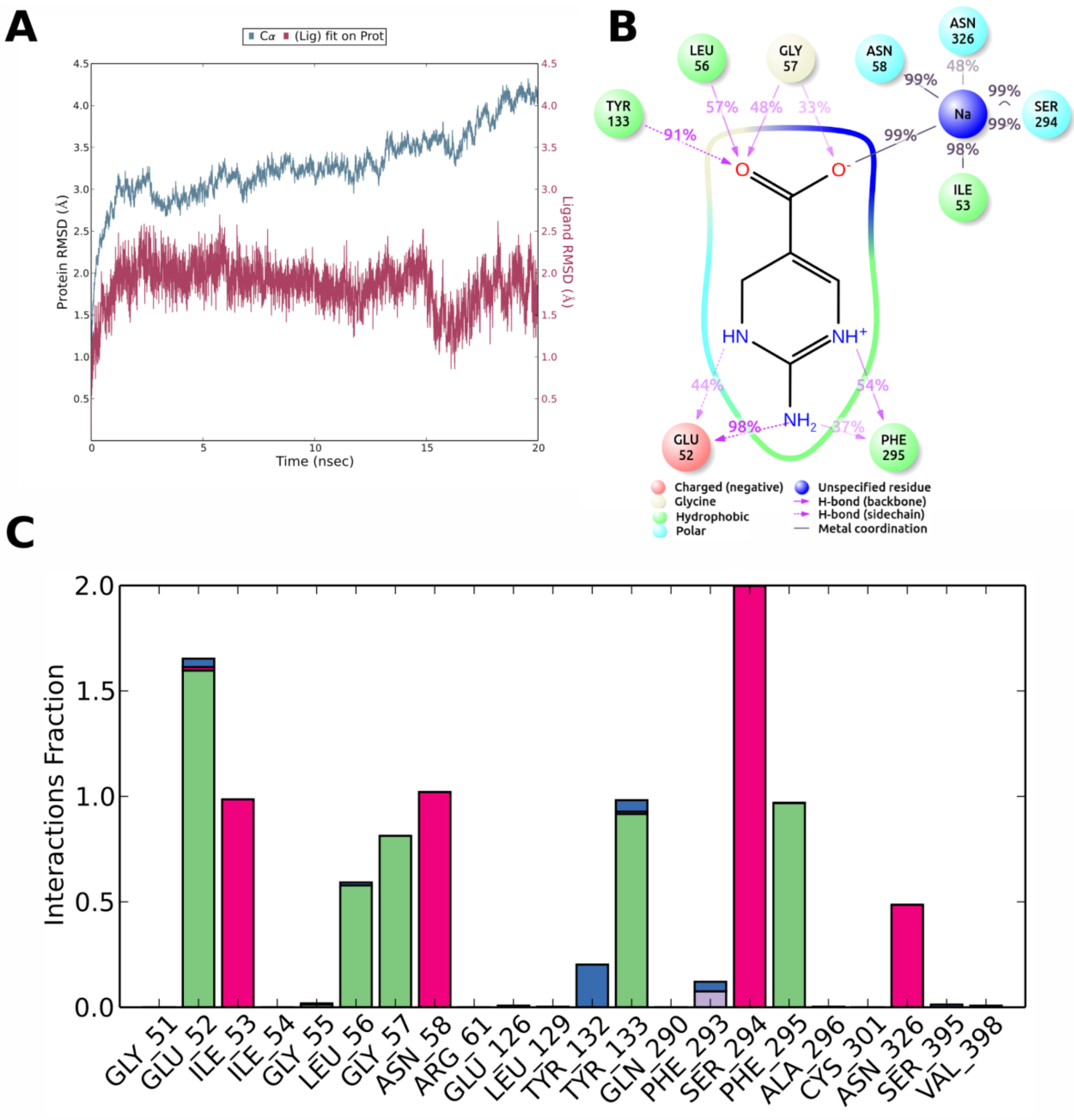

Figure S12. (A) RMSD of one MD simulation run of the highest-scored docking pose of **11** in the hBGT1 Q299L mutant. **(B)** Protein-ligand-interaction schematic overview of the same MD simulation run as in A. Two MD replica showed similar results. **(C)** Protein-ligand interaction diagram of the same MD simulation run as in A (pink, ionic interactions; green, hydrogen bonding; purple, hydrophobic contacts; blue, water contacts). An interaction fraction value of 1.0 indicates that this interaction is maintained 100% of the time during the simulation. Values bigger than 1.0 are possible due to multiple contacts of the ligand with the same residue.

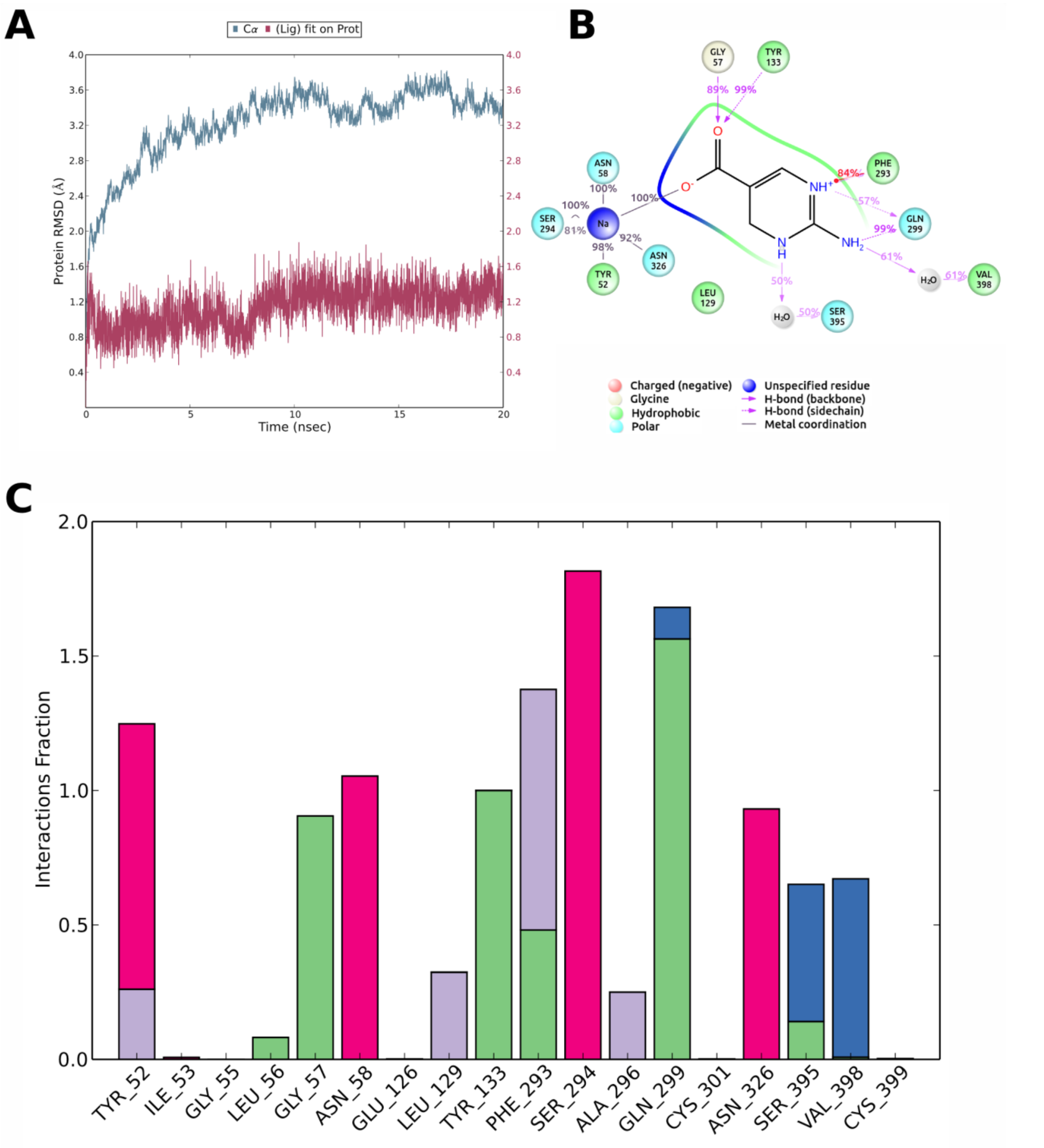

Figure S13. (A) RMSD of one MD simulation run of the highest-scored docking pose of **11** in the hBGT1 E52Y mutant. **(B)** Protein-ligand-interaction schematic overview of the same MD simulation run as in A. Two MD replicas showed similar results. **(C)** Protein-ligand interaction diagram of the same MD simulation as in A (pink, ionic interactions; green, hydrogen bonding; purple, hydrophobic contacts; blue, water contacts). An interaction fraction value of 1.0 indicates that this interaction is maintained 100% of the time during the simulation. Values bigger than 1.0 are possible due to multiple contacts of the ligand with the same residue.

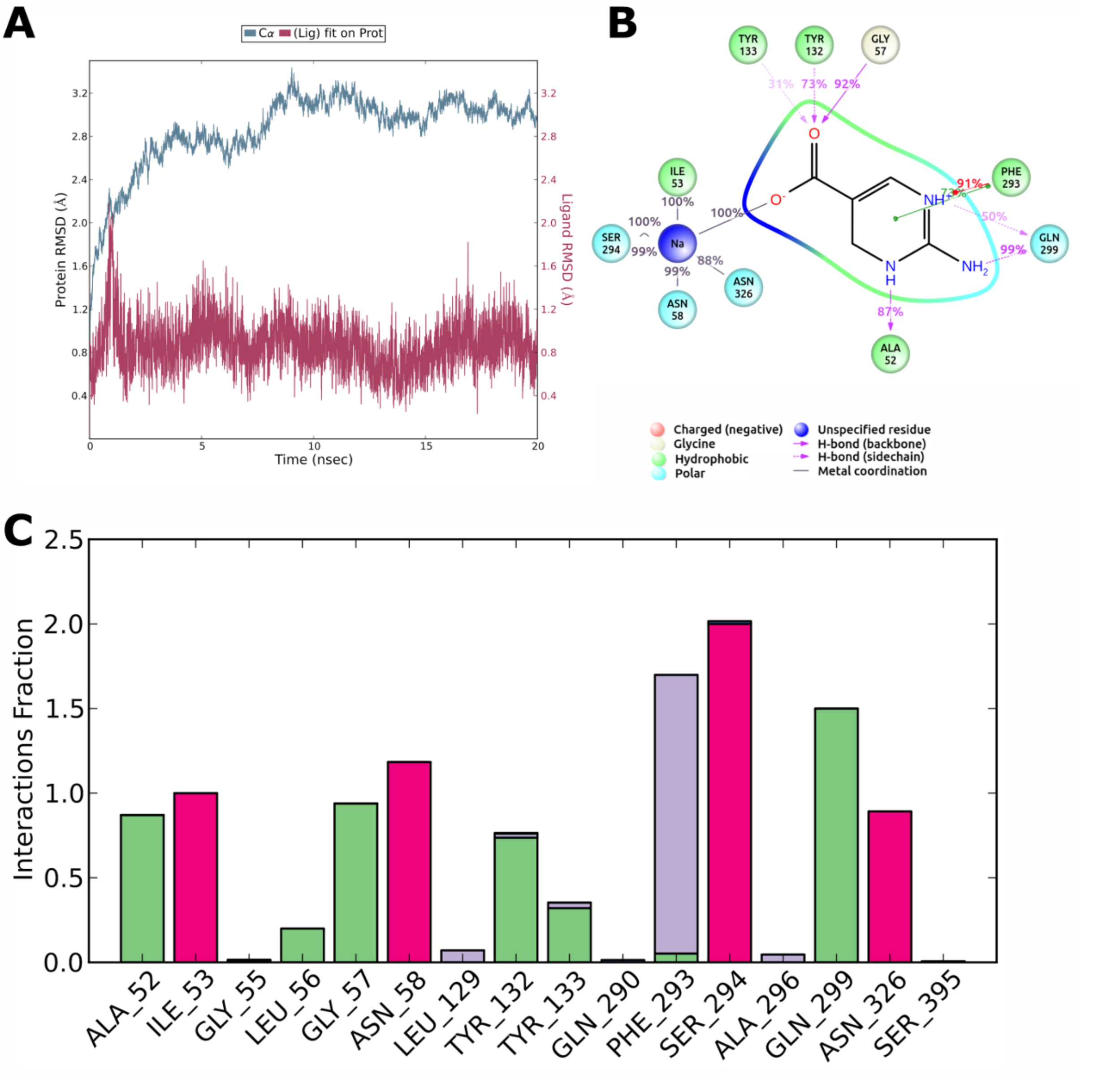

Figure S14. (A) RMSD of one MD simulation run of the highest-scored docking pose of **11** in the hBGT1 E52A mutant. **(B)** Protein-ligand-interaction schematic overview of the same MD simulation as in A. Two MD replicas showed similar results. **(C)** Protein-ligand interaction diagram of the same MD simulation run as in A (pink, ionic interactions; green, hydrogen bonding; purple, hydrophobic contacts; blue, water contacts). An interaction fraction value of 1.0 indicates that this interaction is maintained 100% of the time during the simulation. Values bigger than 1.0 are possible due to multiple contacts of the ligand with the same residue.

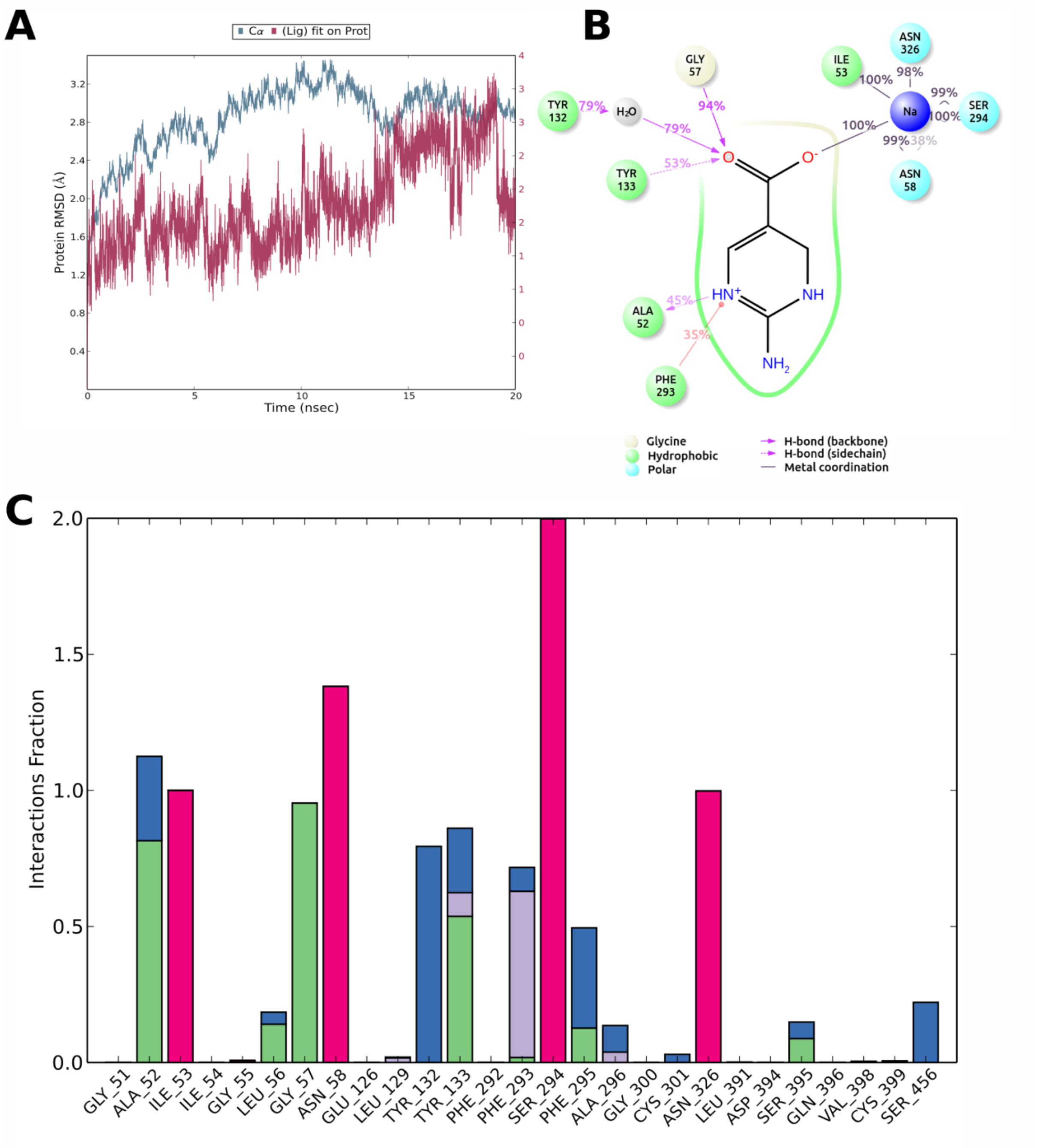

Figure S15. (A) RMSD of one MD simulation run of the highest-scored docking pose of **11** in the hBGT1 E52A+Q299L mutant. **(B)** Protein-ligand-interaction schematic overview of the same MD simulation run as in A. Two MD replicas showed similar results. **(C)** Protein-ligand interaction diagram of the same MD simulation run as in A (pink, ionic interactions; green, hydrogen bonding; purple, hydrophobic contacts; blue, water contacts). An interaction fraction value of 1.0 indicates that this interaction is maintained 100% of the time during the simulation. Values bigger than 1.0 are possible due to multiple contacts of the ligand with the same residue.

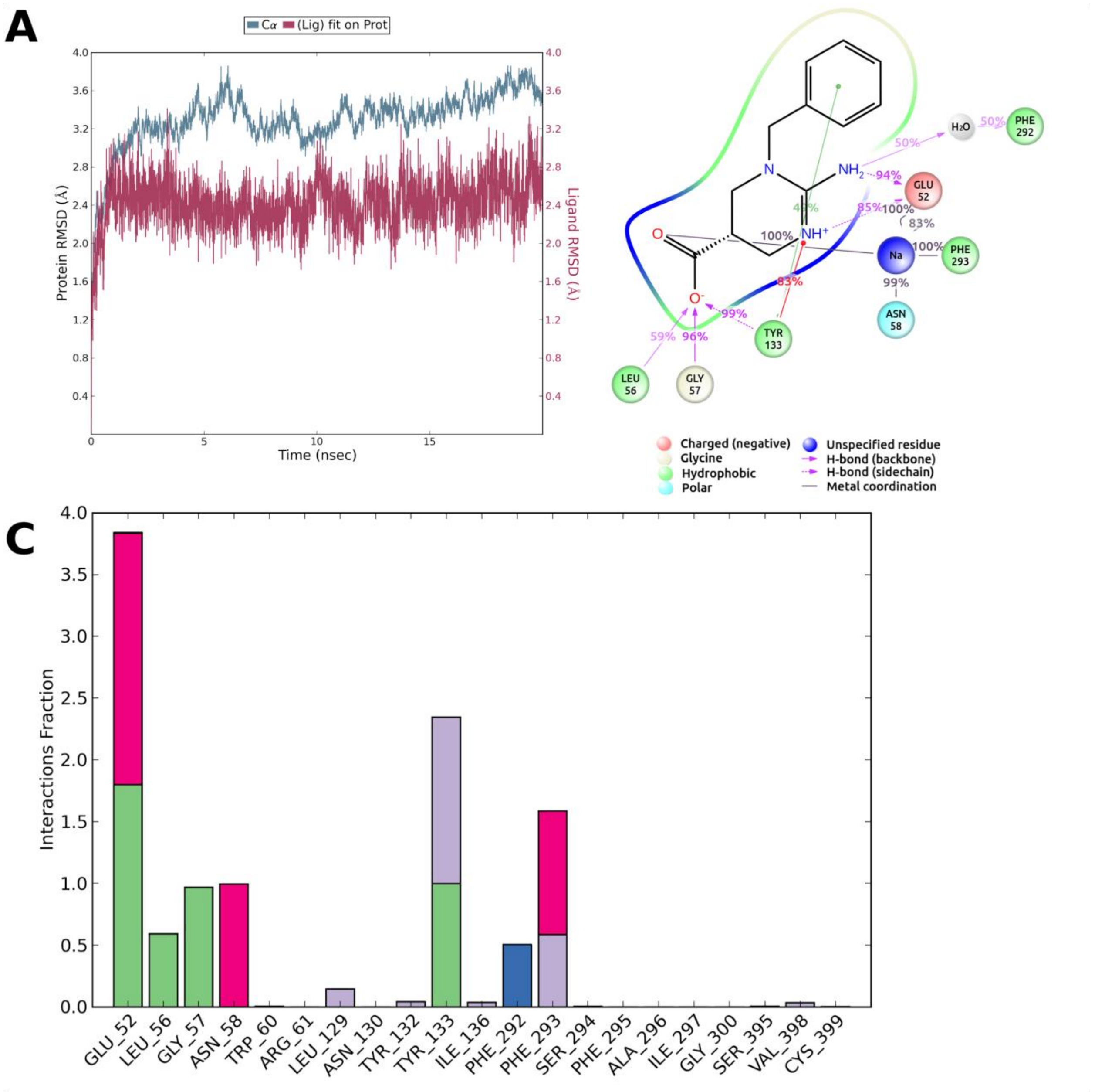

Figure S16. (A) RMSD of one MD simulation run of the highest-scored docking pose of **4** in the hBGT1 Q299L mutant. **(B)** Protein-ligand-interaction schematic overview of the same MD simulation run as in A. Two MD replicas showed similar results. **(C)** Protein-ligand interaction diagram of the same MD simulation run as in A. (pink, ionic interactions; green, hydrogen bonding; purple, hydrophobic contacts; blue, water contacts). An interaction fraction value of 1.0 indicates that this interaction is maintained 100% of the time during the simulation. Values bigger than 1.0 are possible due to multiple contacts of the ligand with the same residue.

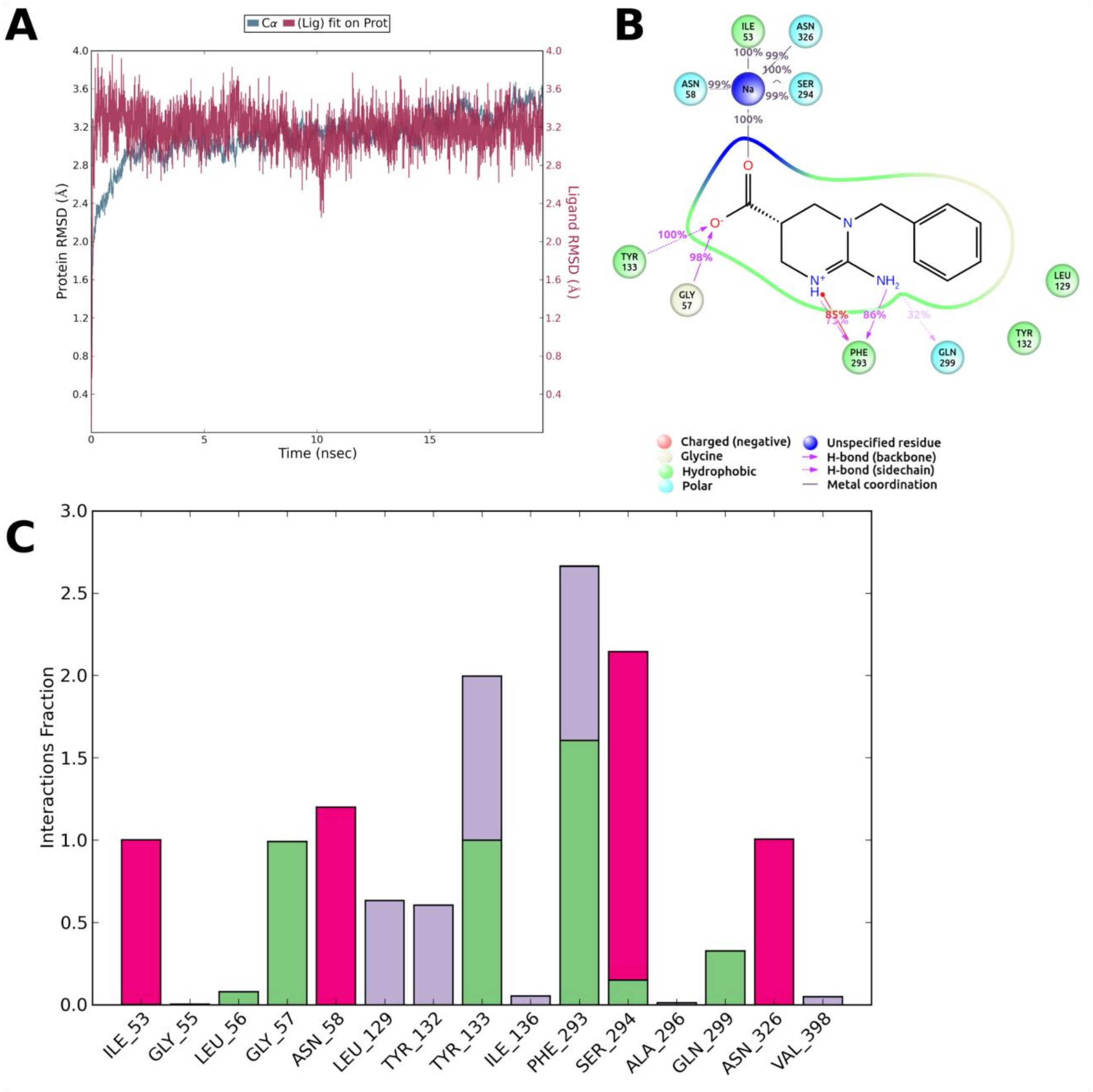

Figure S17. (A) RMSD of one MD simulation run of the highest-scored docking pose of **4** in the hBGT1 E52Y mutant. **(B)** Protein-ligand-interaction schematic overview of the same MD simulation run as in A. Two MD replica showed similar results. **(C)** Protein-ligand interaction diagram of the same MD simulation run as in A. (pink, ionic interactions; green, hydrogen bonding; purple, hydrophobic contacts). An interaction fraction value of 1.0 indicates that this interaction is maintained 100% of the time during the simulation. Values bigger than 1.0 are possible due to multiple contacts of the ligand with the same residue.

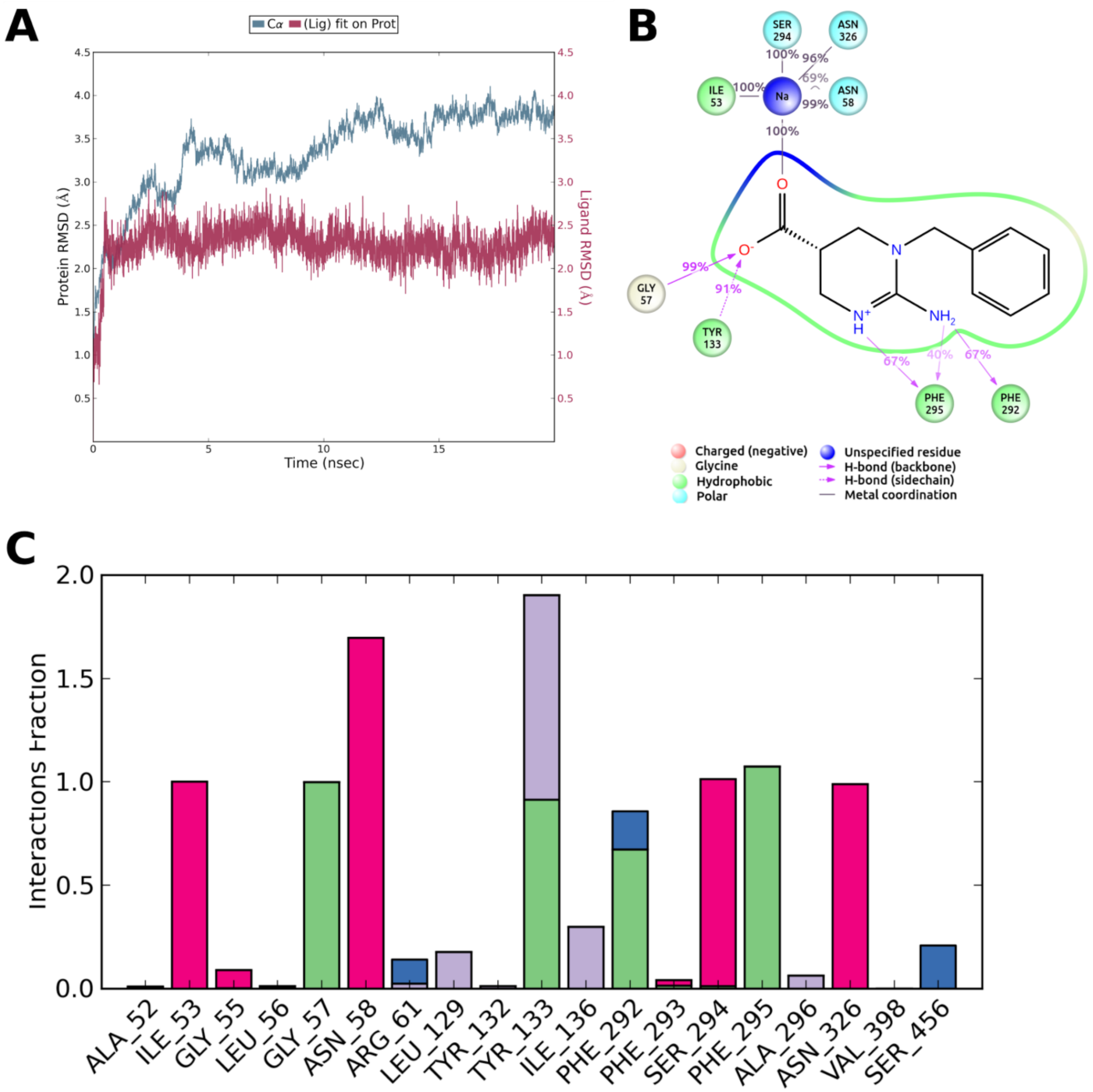

Figure S18. (A) RMSD of one MD simulation run of the highest-scored docking pose of **4** in the hBGT1 E52A mutant. **(B)** Protein-ligand-interaction schematic overview of the same MD simulation run as in A. Two MD replicas showed similar results. **(C)** Protein-ligand interaction diagram of the same MD simulation run as in A. (pink, ionic interactions; green, hydrogen bonding; purple, hydrophobic contacts; blue, water contacts). An interaction fraction value of 1.0 indicates that this interaction is maintained 100% of the time during the simulation. Values bigger than 1.0 are possible due to multiple contacts of the ligand with the same residue.

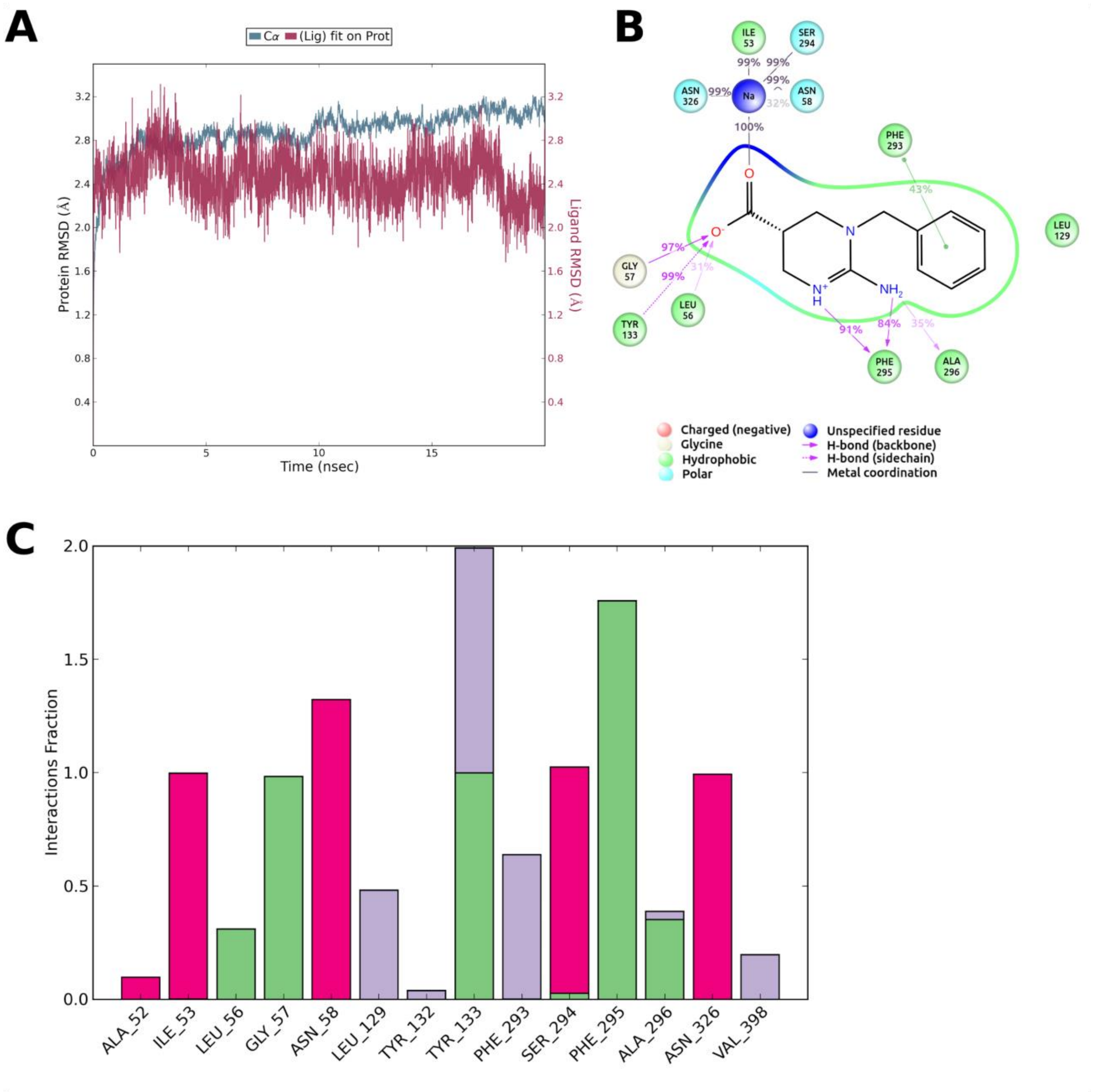

Figure S19. (A) RMSD of one MD simulation run of the highest-scored docking pose of **4** in the hBGT1 E52A+Q299L mutant. **(B)** Protein-ligand-interaction schematic overview of the same MD simulation run as in A. Two MD replica showed similar results. **(C)** Protein-ligand interaction diagram of the same MD simulation run as in A. (pink, ionic interactions; green, hydrogen bonding; purple, hydrophobic contacts). An interaction fraction value of 1.0 indicates that this interaction is maintained 100% of the time during the simulation. Values bigger than 1.0 are possible due to multiple contacts of the ligand with the same residue.

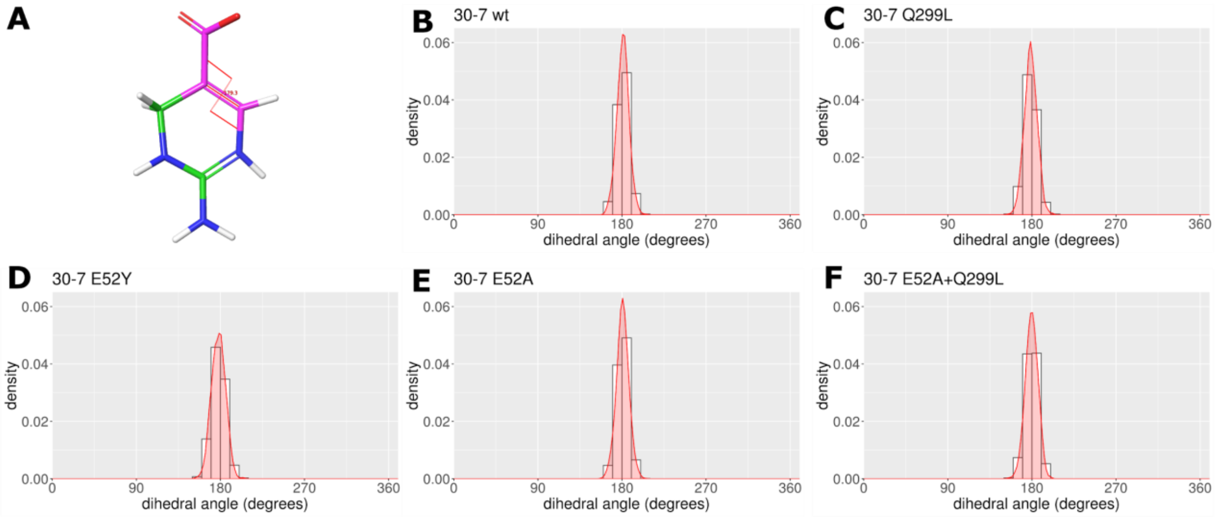

Figure S20. (A) Illustration of the measured dihedral angle between the carboxyl group and the dihydropyrimidine ring of **11**. **(D-F)** Density distributions of dihedral angles between the carboxyl group and the dihydropyrimidine ring of **11** in the simulations of **11** in the wt hBGT1 and all mutants (bin width corresponds to ten degrees). Every tenth snapshot of every trajectory including all replicas were extracted (417 snapshots per simulation) and the dihedral angles were calculated with vmd.[1]

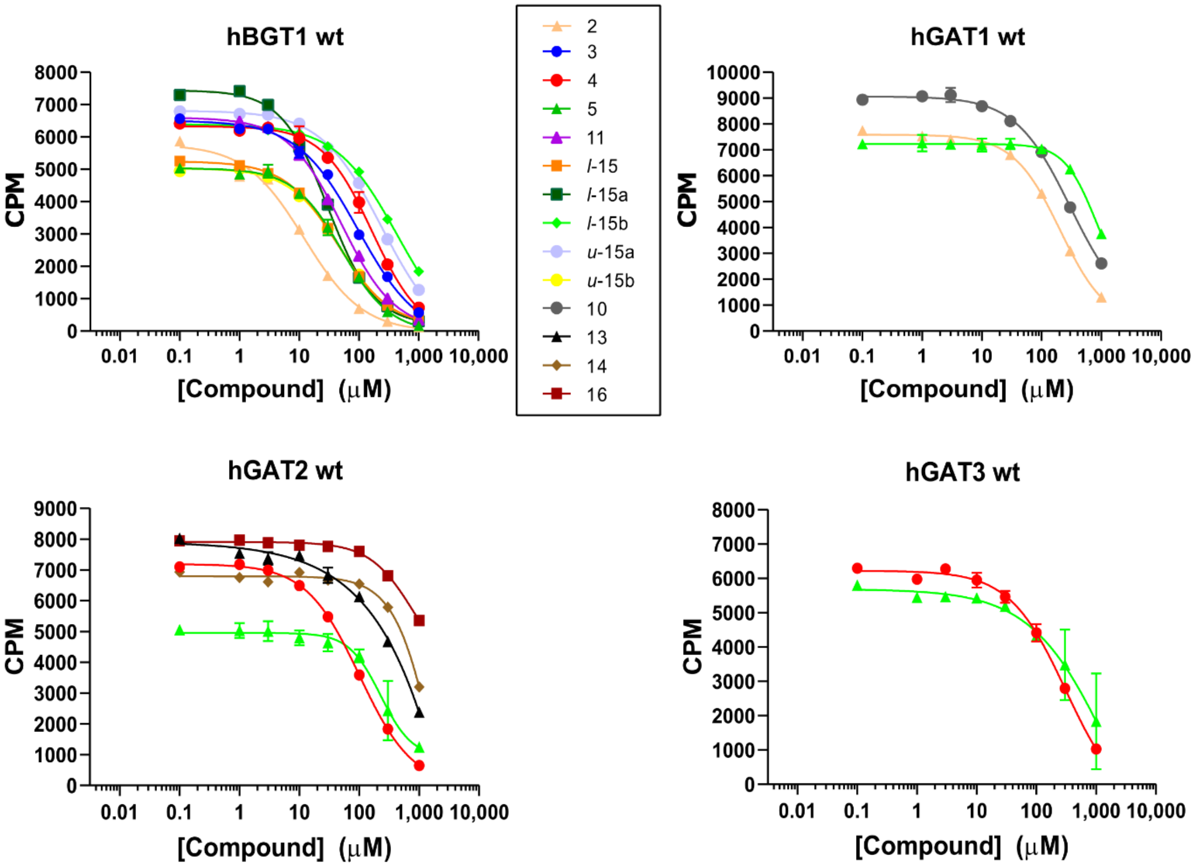

Figure S21. Concentration-response curves of selected compounds of recombinant hGATs stably expressed in CHO Flp-In cells (all compounds from Table 1 where an IC_{50} could be obtained). The compounds were tested for their ability to inhibit the uptake of 30 nM [^{3}H]GABA for 3 min. The curves are given as means ± SD of triplicate measurements of a single representative experiment. At least two additional independent experiments gave similar results, and the IC_{50} (pIC50 ± S.E.M.) values obtained are listed in Table 1. A control for maximal uptake inhibition was determined in the presence of GABA (3 mM). The concentration-response curves of **8** and **9** were tested at hBGT1 transiently expressed in tsA201 cells and are depicted in the supplementary Fig. S22 below.

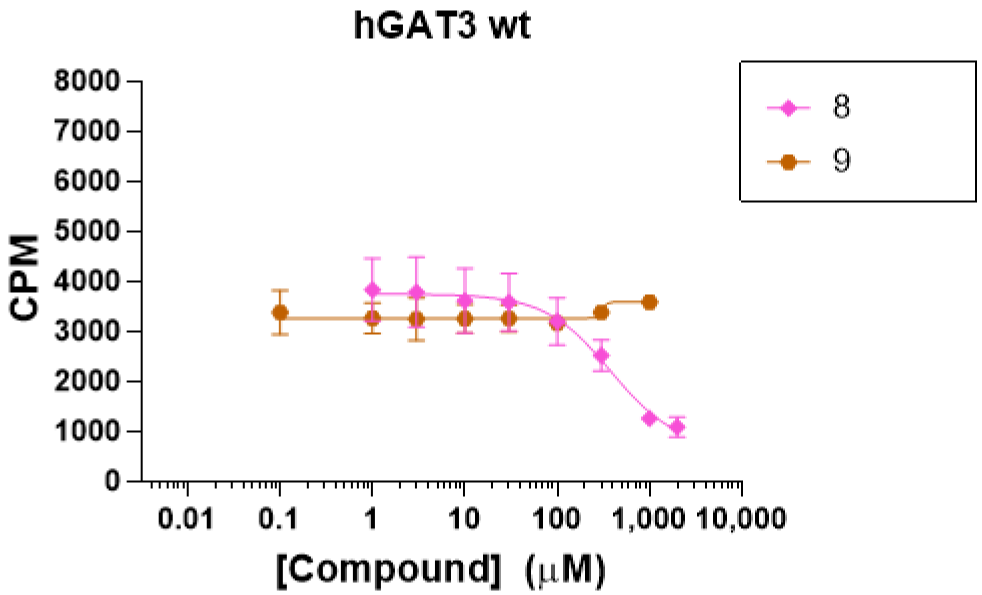

Figure S22. Concentration-response curves of **8** and **9** tested at wildtype (wt) hBGT1 transiently expressed in tsA201 cells. The compounds were tested for their ability to inhibit the uptake of 30 nM [^{3}H]GABA for 3 min. The curves are given as means ± SD of triplicate measurements of a single representative experiment. At least two additional independent experiments gave similar results, and the IC50 (pIC50 ± S.E.M.) values obtained are listed in Table 1.

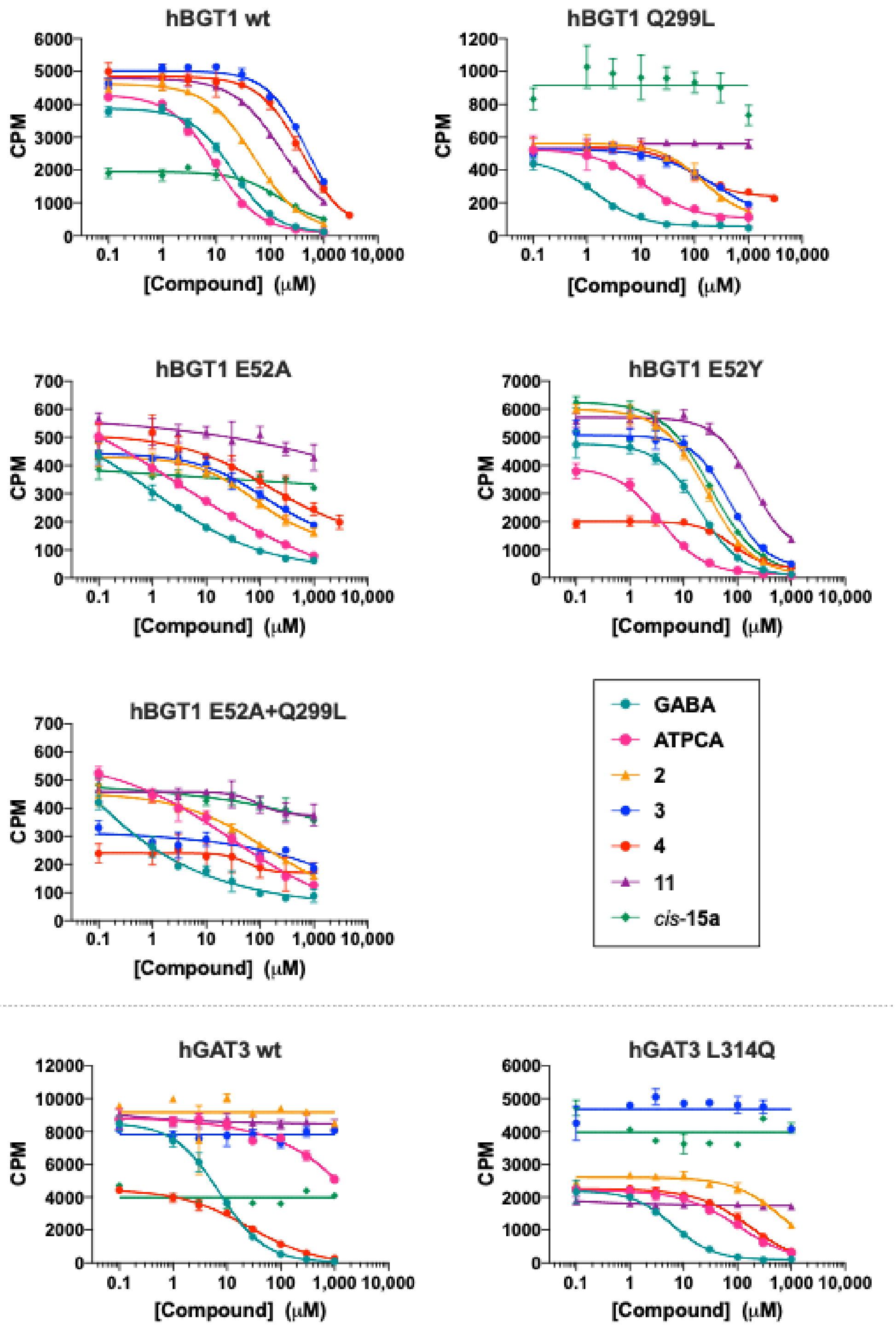

Figure S23. Concentration-response curves of all compounds from Table 2 tested at wildtype (wt) hBGT1, wt hGAT3 and at mutated transporters transiently expressed in tsA201 cells. The compounds were tested for their ability to inhibit the uptake of 30 nM [^{3}H]GABA for 3 min. The curves are given as means ± SD of triplicate measurements of a single representative experiment. At least two additional independent experiments gave similar results, and the IC_{50} (pIC_{50} ± S.E.M.)

values obtained are listed in Table 2. A control for maximal uptake inhibition was determined in the presence of GABA (3 mM).

SYNTHETIC PROCEDURES

General procedures. All reagents and solvents were obtained from commercial suppliers and used without further purification. Air- and/or moisture-sensitive reactions were performed under a nitrogen atmosphere using syringe-septum cap techniques and with the use of flame-dried glassware. Anhydrous solvents were obtained by using a solvent purification system (THF, DMF and DCM) or by storage over 4 Å molecular sieves. Thin-layer chromatography (TLC) was carried out using Merck silica gel 60 F254 plates, and compounds were visualized by UV (254 and 366 nm), anisaldehyde or ninhydrin spray reagent. Flash chromatography (FC) was carried out according to standard procedures using Merck silica gel 60 (0.040–0.063 mm). Melting points were recorded on an SRS OptiMelt apparatus in open capillary tubes and are uncorrected. ^{1}H and ^{13}C NMR data were recorded on a 300 MHz Varian Mercury 300BB spectrometer equipped with a 5mm ^{1}H (BB) probe, a 300 MHz Varian Gemini 2000BB spectrometer equipped with a 5 mm ^{31}P, ^{13}C (^{1}H, ^{19}F) probe, a Bruker Avance 400 MHz spectrometer equipped with a 5mm PABBO BB (^{1}H, ^{19}F) Z-GRD probe, or a Bruker Avance 600 MHz spectrometer equipped with a cryogenically cooled 5 mm CPDCH ^{13}C(^{1}H) Z-GRD probe, at 300 K. Analytical HPLC (Anal. HPLC) was performed on a Merck-Hitachi HPLC system consisting of an L-7100 pump, an L-7200 autosampler, and an L-7400 UV detector (210 or 254 nm), using an X-Terra column (4.6 x 50 mm). A linear gradient elution was performed with eluent A (H_2O/TFA 100:0.1) containing 0% of solvent B (MeCN/H_2O/TFA, 90:10:0.01) rising to100 % of B during 5 min with a flow rate of 4.0 mL/min. Final compounds were analyzed on an analytical HPLC system consisting of an Ultimate 3000 pump (Dionex), an AS3000 autosampler (Thermo Scientific), and an Ultimate Photodiode Array Detector (Dionex) (210 nm) using a Luna 3μM C18(2) column (150 x 4.6 mm) and a mobile phase consisting of A (H_2O/TFA 100:0.1) containing 0% of solvent B (MeCN/H_2O/TFA, 90:10:0.01) for 5 min rising in a linear gradient to 90% of B for 15 min at a flow rate of 1 mL/min. HPLC purity >95% unless otherwise stated. Preparative chiral HPLC was carried out on a Jascu HPLC system consisting of a 880-PU pump (Jascu), a SpectraSeries UV100 detector (210 nm) and a Merck-Hitachi D-2000 Chromato-Integrator using a preparative ChirobioticT column (25 cm x 21.2mm, 5 μm) and a mobile phase consisting of EtOH/H_2O (NH_4OAc 20mM, pH=4) (30:70, v/v), and a flow rate of 5 mL/min. Enantiomeric purity of compounds *l*-**15a,b** (*l*-**15a** – Rt= 8.29 min, *l*-**15b** – Rt= 11.53 min) and *u*-**15a,b** (*u*-**15a** – Rt= 8.80 min, *u*-**15b** – Rt= 10.17 min) was determined to be >95% using a ChirobioticT column (15 cm x 4.6 mm, 5 μm) and a mobile phase consisting of H_2O (NH_4OAc 20 mM, pH=4)/EtOH (30:70, v/v), and a flow rate of 1 mL/min.

Synthetic procedures of ATPCA analogs

Ethyl 2-acetamidopyrimidine-5-carboxylate (**21**). **20** (413 mg, 2.47 mmol, 1 eq) was refluxed in acetic anhydride (20 mL) at 160 °C for 4 days. The solution was reduced in vacuo and purified by FC (8:2 EtOAc/Heptane) yielding the product as a white solid (322 mg, 62%). ^{1}H-NMR (400 MHz; DMSO-d_6): δ 10.99–10.97 (m, 1H), 9.05 (s, 2H), 4.34 (q, J = 7.1 Hz, 2H), 2.23 (s, 3H), 1.35–1.31 (m, 3H). ^{13}C-NMR (101 MHz, DMSO-d_6): δ 169.65, 163.74, 160.21, 159.84, 119.01, 61.53, 25.41, 14.54.

Ethyl 2-acetamido-1,4,5,6-tetrahydropyrimidine-5-carboxylate acetate (**22**). **21** (1.5 g, 7.17 mmol, 1 eq) was suspended in EtOH and AcOH was added until a yellow solution was obtained. The mixture was hydrogenated over Pd/C (wt.10%, 2.3 g, 2.15 mmol 0.3 eq) for 24 hr and then filtered through cotton. The organic solvent was evaporated *in vacuo* and co-evaporated with heptane to afford the product as a white solid (1.95 g, 99%). ^{1}H-NMR (600 MHz; $CDCl_3$): δ 4.21 (q, *J* = 7.1 Hz, 2H), 3.65 (dd, *J* = 13.0, 4.9 Hz, 2H), 3.56 (dd, *J* = 13.1, 8.1 Hz, 2H), 2.94 (tt, *J* = 8.1, 4.8 Hz, 1H), 2.20 (d, *J* = 10.4 Hz, 3H), 2.01 (s, 3H, AcOH), 1.27 (t, *J* = 7.1 Hz, 3H). ^{13}C-NMR (151 MHz, $CDCl_3$): δ 178.07, 175.31 (AcOH), 169.33, 152.62, 61.94, 39.51, 35.10, 24.11, 22.68 (AcOH), 14.03.

General procedure for N-alkylation (**23**–**27**). Unless otherwise stated, a flame-dried flask was charged with **22** (1 eq), Cs_2CO_3 (3.6–4 eq) and KI (0.4 eq) and put under N_2 atmosphere. Dry DMF was added and the suspension was allowed to stir for 10 min – 6 hr following addition of alkylating agent (1.4–2.4 eq). After completion, the mixture was diluted with EtOAc, filtered and evaporated under reduced pressure and purified by FC.

Ethyl 2-acetamido-1-ethyl-1,4,5,6-tetrahydropyrimidine-5-carboxylate (**23**). Synthesized according to general procedure for *N*-alkylation using **22** (289 mg, 1.06 mmol, 1 eq), Cs_2CO_3 (1.23 g, 3.77 mmol, 3.6 eq) and ethyliodide (0.2 mL, 2.50 mmol, 2.4 eq). Stirred 10 min prior to addition of alkylating agent. Reaction mixture stirred at r.t. for 5 days and purified by FC (2:1 heptane/EtOAc to 9:1 DCM/MeOH). Yielding the product as a yellow solid (33 mg, 11%). ^{1}H-NMR (600 MHz; $CDCl_3$): δ 4.25 (q, J = 7.1 Hz, 3H), 3.74–3.66 (m, 5H), 3.04–3.00 (m, 1H), 2.30 (s, 3H), 1.32–1.28 (m, 6H). ^{13}C-NMR (151 MHz, $CDCl_3$): δ 183.44, 170.27, 157.05, 61.54, 45.41, 40.23, 36.84, 30.90, 28.19, 14.10, 12.53.

Ethyl 2-acetamido-1-benzyl-1,4,5,6-tetrahydropyrimidine-5-carboxylate (**24**). Synthesized according to general procedure for *N*-alkylation using **22** (621 mg, 2.27 mmol, 1 eq), Cs_2CO_3 (2.8 g, 9.09 mmol, 4 eq) and benzylbromide (0.5 mL, 4.21 mmol, 1.9 eq). Stirred 10 min prior to addition of alkylating agent. Reaction mixture stirred at r.t. for 3 days and purified by FC (100% EtOAc) yielding the product as a white solid (268 mg, 39%). ^{1}H-NMR (600 MHz; $CDCl_3$): δ 7.38–7.27 (m, 5H), 5.01–4.88 (m, 1H), 4.86–4.67 (m, 1H), 4.12 (tdtd, J = 13.1, 12.2, 7.2, 6.7 Hz, 2H), 3.62–3.55 (m, 1H), 3.54–3.48 (m, 1H), 3.44–3.41 (m, 2H), 2.93–2.87 (m, 1H), 2.20–2.02 (m, 3H). ^{13}C-NMR (151 MHz, $CDCl_3$): δ 183.92, 170.04, 157.60, 157.58, 137.13, 137.10, 128.65, 128.09, 127.65, 61.53, 45.45, 40.29, 36.79, 29.69, 14.05.

Ethyl 1-([1,1'-biphenyl]-4-ylmethyl)-2-acetamido-1,4,5,6-tetrahydropyrimidine-5-carboxylate (**25**). Synthesized according to general procedure for *N*-alkylation using **22** (308 mg, 1.13 mmol,

1 eq), Cs_2CO_3 (1.35 g, 4.14 mmol, 3.7 eq) and 4-(bromomethyl)-1,1'-biphenyl (386 mg, 1.56 mmol, 1.4 eq). Stirred 10 min prior to addition of alkylating agent. Stirred at r.t. for 24 hr and then at 60 °C for 3 hr. Purified by FC (100% EtOAc) yielding the product as a white solid (29 mg, 7%). ^{1}H-NMR (600 MHz; $CDCl_3$): δ 7.60–7.55 (m, 4H), 7.45–7.42 (m, 2H), 7.38–7.33 (m, 3H), 4.96 (d, J = 14.8 Hz, 1H), 4.78 (d, J = 14.8 Hz, 1H), 4.13 (qq, J = 12.4, 7.1 Hz, 2H), 3.60 (dd, J = 12.7, 4.6 Hz, 1H), 3.51 (dd, J = 12.7, 8.5 Hz, 1H), 3.47–3.43 (m, 2H), 2.94–2.89 (m, 1H), 2.12 (s, 3H), 1.23–1.20 (m, 3H). ^{13}C-NMR (151 MHz, $CDCl_3$): δ 183.84, 170.11, 157.57, 140.71, 140.59, 136.11, 128.81, 128.53, 127.36, 127.04, 61.52, 51.56, 45.47, 40.32, 36.85, 28.31, 14.06.

Ethyl 2-acetamido-1-(3,3-diphenylpropyl)-1,4,5,6-tetrahydropyrimidine-5-carboxylate (**26**). Synthesized according to general procedure for *N*-alkylation using **22** (336 mg, 1.23 mmol, 1 eq), Cs_2CO_3 (1.6 g, 4.92 mmol, 4 eq), KI (82 mg, 0.49 mmol, 0.4 eq) and (4-bromobutane-1,1-diyl)dibenzene (508 mg, 1.85 mmol, 1.5 eq). Stirred 6 hr prior to addition of alkylating agent. Reaction mixture stirred at r.t. for 4 days and purified by FC (100% EtOAc) yielding the product as a colourless oil (56 mg, 11%). ^{1}H-NMR (600 MHz; MeOD-d_4): δ 7.33–7.24 (m, 8H), 7.18–7.15 (m, 2H), 4.19–4.11 (m, 2H), 3.96 (t, J = 7.8 Hz, 1H), 3.57–3.40 (m, 6H), 2.36 (q, J = 7.7 Hz, 2H), 1.99–1.93 (m, 3H), 1.26–1.22 (m, 3H). ^{13}C-NMR (151 MHz, MeOD-d_4): δ 181.91, 170.59, 157.48, 144.67, 144.55, 128.13, 127.46, 127.44, 125.90, 61.07, 48.76, 48.01, 45.87, 39.63, 36.13, 32.70, 26.19, 13.04.

Ethyl 2-acetamido-1-(4,4-diphenylbutyl)-1,4,5,6-tetrahydropyrimidine-5-carboxylate (**27**). Synthesized according to general procedure for *N*-alkylation using **22** (252 mg, 0.922 mmol, 1 eq), Cs_2CO_3 (1.24 g, 3.81 mmol, 4 eq), KI (67 mg, 0.404 mmol, 0.4 eq) and (4-bromobutane-1,1-diyl)dibenzene (400 mg, 1.38 mmol, 1.5 eq). Stirred 6 hr prior to addition of alkylating agent. Reaction mixture stirred at r.t. for 24 hr and purified by FC (8:2 EtOAc/heptane + ammonia) yielding the product as a colourless film (51 mg, 13%). ^{1}H-NMR (600 MHz; $CDCl_3$): δ 7.31–7.26 (m, 8H), 7.22–7.18 (m, 2H), 4.21–4.16 (m, 2H), 4.04 (t, J = 7.9 Hz, 1H), 3.56 (dt, J = 12.4, 6.1 Hz, 2H), 3.50–3.37 (m, 3H), 2.91–2.87 (m, 1H), 2.08 (q, J = 7.6 Hz, 4H), 1.58 (quintet, J = 7.5 Hz, 2H), 1.30–1.25 (m, 3H). ^{13}C-NMR (151 MHz, $CDCl_3$): δ 183.59, 170.19, 157.38, 144.80, 128.47, 127.83, 126.19, 61.51, 50.58, 45.81, 40.21, 36.79, 32.36, 28.28, 25.55, 14.11.

Ethyl 2-acetamido-1-(4,4-bis(3-methylthiophen-2-yl)but-3-en-1-yl)-1,4,5,6-tetrahydropyrimidine-5-carboxylate (**28**). A suspension of **22** (100 mg, 0.47 mmol, 1 eq) and Li_2CO_3 (1.1 eq) in AcO*i*Pr (1 ml) was stirred under reflux for 30 minutes. A solution of 1,1-Bis(3-methyl-2-thienyl)but-1-en-4-yl methanesulfonate (239 mg, 0.70 mmol, 1.5 eq) dissolved in AcOiPr (1 ml) was added and the resulting mixture was refluxed for 12 hr while continuously adding Li_2CO_3 in small amounts (4 x 16 mg, 0.42 mmol, 0.9 eq). The suspension was diluted in AcOEt, filtered, concentrated under reduced pressure and purified by FC (1:3 *n*-heptane/EtOAc), providing the product as an orange oil (10 mg, 5%). ^{1}H NMR (400 MHz; $CDCl_3$): δ 10.54 (s, 1H), 7.15 (d, J = 5.0 Hz, 1H), 6.98 (d, J = 5.1 Hz, 1H), 6.78 (d, J = 5.0 Hz, 1H), 6.68 (d, J = 5.1 Hz, 1H), 6.01 (t, J = 7.4 Hz, 1H), 4.10 (q, J = 7.1 Hz, 2H), 3.63 (t, J = 7.0 Hz, 2H), 3.48 (dd, J = 12.6, 5.0 Hz, 1H), 3.39 (dd, J = 12.6, 8.4 Hz, 1H), 3.36 – 3.25 (m, 2H), 2.82 (tt, J = 8.4, 5.0 Hz, 1H), 2.37 (q, J = 7.1 Hz, 2H), 1.99 (s, 3H), 1.96 (s, 3H), 1.89 (s, 3H), 1.18 (t, J = 7.1 Hz, 3H). ^{13}C NMR

(101 MHz, $CDCl_3$) δ 182.72, 169.18, 156.38, 138.22, 134.51, 134.02, 132.84, 130.42, 130.26, 128.60, 128.48, 123.45, 121.96, 60.45, 47.01, 44.94, 39.24, 35.82, 27.30, 27.12, 13.67, 13.39, 13.10.

General procedure for hydrolysis (**3**–**7**). The protected compounds (**23**–**27**) were treated with 4 M HCl and heated at 90 °C using conventional heating for 1 to 4 days followed by evaporation under vacuum.

2-Amino-1-ethyl-1,4,5,6-tetrahydropyrimidine-5-carboxylic acid hydrochloride (**3**). Hydrolysis according to general procedure using **23** (32 mg, 0.133 mmol, 1 eq), heated for 2 days and triturated with Et_2O yielding the product as a light brown solid (22 mg, 81%). ^{1}H-NMR (600 MHz; D_2O): δ 3.71–3.65 (m, 2H), 3.60–3.57 (m, 1H), 3.52 (dd, J = 12.7, 4.6 Hz, 1H), 3.41 (qq, J = 18.6, 7.4 Hz, 2H), 3.24 (quintet, J = 4.9 Hz, 1H), 1.21 (d, J = 14.5 Hz, 3H). ^{13}C-NMR (151 MHz, D_2O): δ 174.57, 152.79, 45.99, 44.56, 39.30, 10.98. mp: (decomp.) > 150 °C. Purity by anal. HPLC (210 nm): 99%.

2-Amino-1-benzyl-1,4,5,6-tetrahydropyrimidine-5-carboxylic acid hydrochloride (**4**). Hydrolysis according to the general procedure using **24** (40 mg, 0.132 mmol, 1 eq), heated for 2 days and triturated with Et_2O yielding the product as a white solid (34 mg, 95%). ^{1}H-NMR (600 MHz; D_2O): δ 7.40–7.32 (m, 3H), 7.25 (d, J = 7.4 Hz, 2H), 4.57 (d, J = 16.8 Hz, 1H), 4.48 (d, J = 16.8 Hz, 1H), 3.56–3.45 (m, 4H), 3.12 (q, J = 5.0 Hz, 1H). ^{13}C-NMR (151 MHz, D_2O): δ 174.19, 153.68, 134.28, 129.03, 128.24, 127.11, 52.58, 46.62, 39.25, 36.27. mp: (decomp.) > 166 °C. Purity by anal. HPLC (210 nm): 99%.

1-([1,1'-Biphenyl]-4-ylmethyl)-2-amino-1,4,5,6-tetrahydropyrimidine-5-carboxylic acid hydrochloride (**5**). Hydrolysis according to the general procedure using **25** (28 mg, 0.0738 mmol, 1 eq), heated for 3 days and triturated with Et_2O and filtered, yielding the product as a white solid (17 mg, 66%). ^{1}H-NMR (400 MHz; MeOD-d_4): δ 7.74–7.59 (m, 4H), 7.51–7.31 (m, 5H), 4.79–4.65 (m, 2H), 3.69–3.54 (m, 4H), 3.21–3.13 (m, 1H). ^{13}C-NMR (151 MHz, MeOD-d_4): δ 171.78, 153.99, 141.07, 140.30, 133.32, 128.53, 127.51, 127.19, 126.53, 52.28, 46.74, 39.59, 36.41. mp: (decomp.) > 156 °C. Purity by anal. HPLC (254 nm): 97%.

2-Amino-1-(3,3-diphenylpropyl)-1,4,5,6-tetrahydropyrimidine-5-carboxylic acid hydrochloride (**6**). Hydrolysis according to the general procedure using **26** (56 mg, 0.137 mmol, 1 eq), heated for 3 days and triturated with Et_2O, yielding the product as a white solid (53 mg, 96%). ^{1}H-NMR (600 MHz; MeOD-d_4): δ 7.34–7.23 (m, 8H), 7.20–7.14 (m, 2H), 4.07 (t, J = 7.8 Hz, 1H), 3.49–3.42 (m, 3H), 3.36 (ddd, J = 19.3, 10.5, 5.4 Hz, 2H), 3.30–3.24 (m, 1H), 2.98 (t, J = 4.6 Hz, 1H), 2.49–2.38 (m, 2H). ^{13}C-NMR (151 MHz, MeOD-d_4): δ 171.76, 153.27, 144.10, 143.96, 128.39, 128.34, 127.38, 127.31, 126.24, 126.19, 48.65, 48.43, 46.86, 39.40, 36.10, 31.90. mp: (decomp.) > 200 °C. Purity by anal. HPLC (254 nm): 98%.

2-Amino-1-(4,4-diphenylbutyl)-1,4,5,6-tetrahydropyrimidine-5-carboxylic acid hydrochloride (**7**). Hydrolysis according to the general procedure using **27** (51 mg, 0.12 mmol, 1 eq), heated for 4 days and triturated with Et_2O, yielding the product as a white solid (33 mg, 71%). ^{1}H-NMR (600 MHz; MeOD-d_4): δ 7.30–7.25 (m, 8H), 7.17–7.14 (m, 2H), 3.96 (t, J = 7.9 Hz, 1H), 3.56–3.44 (m, 4H), 3.40–3.35 (m, 2H), 3.05 (quintet, J = 5.1 Hz, 1H), 2.13–2.09 (m, 2H), 1.63–1.58 (m, 2H).

^{13}C-NMR (151 MHz, MeOD-d_4): δ 171.75, 153.28, 144.76, 144.72, 128.11, 127.49, 125.88, 51.00, 49.34, 46.58, 39.47, 36.19, 31.81, 25.17. mp: (decomp.) > 200 °C. Purity by anal. HPLC (210 nm): 99%.

Sodium 2-amino-1-(4,4-bis(3-methylthiophen-2-yl)but-3-en-1-yl)-1,4,5,6-tetrahydropyrimidine-5-carboxylate (**8**). The protected compound **28** (7 mg, 0.015 mmol, 1 eq) was suspended in 1 ml NaOH 0.5 M and heated to 90 °C for 6 hours. The suspension was centrifuged and the solid pellet was washed with water two times. Lyophilization of the pellet provided the desired compound as an off-white solid (3 mg, 48%). ^{1}H NMR (600 MHz, DMSO-d_6) δ 8.43 (s, 1H), 7.49 (d, J = 5.1 Hz, 1H), 7.32 (bs, 1H), 7.30 (d, J = 5.1 Hz, 1H), 6.94 (d, J = 5.1 Hz, 1H), 6.83 (d, J = 5.1 Hz, 1H), 6.07 (t, J = 7.3 Hz, 1H), 3.42 (m, 2H), 3.27 – 3.13 (m, 3H), 2.53 – 2.51 (m, 2H), 2.33 (m, 2H), 1.99 (s, 3H), 1.94 (s, 3H). ^{13}C NMR (151 MHz, DMSO) δ 171.25, 152.96, 138.58, 135.23, 134.18, 133.45, 131.39, 131.07, 129.80, 128.84, 125.36, 123.67, 48.94, 48.22, 41.18, 38.85, 27.57, 14.47, 14.10. Purity by anal. HPLC (200 nm): 95%.

Ethyl 2-acetamido-1,6-dihydropyrimidine-5-carboxylate TFA (**29**). In a flame dried flask **21** (209 mg, 1 mmol, 1 eq) was suspended in TFA (3 ml) and put under N_2 atmosphere. TFA (3 ml) and Et_3SiH (1.2 ml, 10 mmol, 10 eq) were added dropwise and the reaction was stirred for 24 hr. The solvent was evaporated and the resulting solid was washed with Et_2O and dried, affording the product as a white solid TFA salt (181 mg, 86%). ^{1}H NMR (600 MHz, DMSO-d_6): δ 11.26 (bs, 3H), 7.22 (s, 1H), 4.20 (s, 2H), 4.16 (q, J = 7.1 Hz, 2H), 2.18 (s, 3H), 1.22 (t, J = 7.1 Hz, 3H). ^{13}C NMR (151 MHz, DMSO) δ 173.45, 163.92, 159.79 (q, J = 32.7 Hz), 150.34, 133.07, 117.22 (q, J = 296.9 Hz), 105.22, 60.90, 39.63, 24.81, 14.54.

Ethyl 2-acetamido-1-benzyl-1,6-dihydropyrimidine-5-carboxylate (**30**). A flame-dried flask was charged with **29** (200 mg, 0.95 mmol, 1 eq) and Cs_2CO_3 (1.23 g, 3.78 mmol, 4 eq) and put under N_2 atmosphere. Dry DMF (4 ml) was added and the suspension was allowed to stir for 3 min following addition of benzyl bromide (0.21 ml, 1.8 mmol, 1.9 eq). After 1 h, the mixture was diluted with EtOAc and filtered. The filtrate was washed with 3M $CaCl_2$ solution and the organic layer was dried over anhydrous sodium sulphate, filtered and evaporated under reduced pressure. The crude was then purified by FC (1:1 *n*-heptane/EtOAc), yielding the product as a white solid (80 mg, 28%). ^{1}H NMR (400 MHz, DMSO-d_6): δ 10.20 (s, 1H), 7.42 – 7.26 (m, 6H), 4.94 (s, 2H), 4.16 – 4.08 (m, 4H), 1.98 (s, 3H), 1.19 (t, J = 7.1 Hz, 3H). ^{13}C NMR (101 MHz, DMSO) δ 183.49, 164.52, 154.67, 139.39, 138.22, 129.03, 127.91, 127.88, 102.51, 60.39, 56.00, 50.56, 28.69, 14.70.

2-amino-1-benzyl-1,4-dihydropyrimidine-5-carboxylic acid (**9**). The protected compound **30** (10 mg, 0.033 mmol, 1 eq) was treated with 2 ml of 1M NaOH and the resulting solution was stirred at 90 °C for 5 hours. Upon cooling, the water phase was washed with EtOAc and then evaporated, providing a residue that was dissolved in 4 ml of water. The pH of the solution was adjusted to 7 with 1M HCl and was extracted with EtOAc three times. The organic layer was dried over anhydrous sodium sulfate, filtered and evaporated under vacuum. The resulting crude was triturated with water, yielding the desired compound as a white solid (4 mg, 52%). ^{1}H NMR (400 MHz, DMSO-d_6): δ 12.10 (bs, 1H), 7.39 – 7.25 (m, 7H), 7.18 (s, 1H), 4.66 (s, 2H), 3.95 (s, 2H).

^{13}C NMR (151 MHz, DMSO): δ 166.69, 152.78, 140.20, 138.86, 129.01, 127.74, 127.72, 101.11, 49.49, 40.79. Purity by anal. HPLC (210 nm): 94%.

Ethyl 2-(phenethylamino)pyrimidine-5-carboxylate (**32**). **31** (500 mg, 2.68 mmol, 1 eq) was suspended in 2-propanol (3 mL) and TEA (0.95 mL, 5.36 mmol, 2 eq) and 2-phenylethan-1-amine (0.4 mL, 3.22 mmol, 1.2 eq) was added. The mixture was heated in a closed vial at 100 °C for 3 hr. Upon cooling the reaction mixture solidified. It was stirred with H_2O (20 mL) and the solid was filtered and dried to yield the product as a white solid (641 mg, 88%). ^{1}H-NMR (600 MHz; $CDCl_3$): δ 8.82 (dd, J = 83.3, 0.4 Hz, 2H), 7.32–7.29 (m, 2H), 7.24–7.21 (m, 3H), 5.73 (s, 1H), 4.35 (q, J = 7.1 Hz, 2H), 3.77 (q, J = 6.5 Hz, 2H), 2.93 (t, J = 7.0 Hz, 2H), 1.37 (d, J = 14.3 Hz, 3H). ^{13}C-NMR (151 MHz, $CDCl_3$): δ 164.72, 163.33, 160.27, 160.03, 138.69, 128.80, 128.66, 126.57, 113.87, 60.67, 42.75, 35.53, 14.35.

Ethyl 2-(phenethylamino)-1,4,5,6-tetrahydropyrimidine-5-carboxylate (**33**). **32** (50 mg, 0.184 mmol, 1 eq) was dissolved in MeOH (15 mL) and AcOH (0.1 mL) and Pd/C (10 wt.%, 110 mg, 0.10 mmol, 0.5 eq) was added. The reaction was hydrogenated overnight, filtered and evaporated to give a colourless film (51 mg, 98%). ^{1}H-NMR (600 MHz; MeOD-d_4): δ 7.34–7.31 (m, 2H), 7.26–7.23 (m, 3H), 4.22 (q, J = 7.1 Hz, 2H), 3.57 (dd, J = 12.6, 6.1 Hz, 2H), 3.51 (dd, J = 12.6, 4.7 Hz, 2H), 3.41–3.36 (m, 2H), 3.07 (td, J = 5.4, 1.3 Hz, 1H), 2.86 (t, J = 7.1 Hz, 2H), 1.29 (t, J = 7.1 Hz, 3H). ^{13}C-NMR (151 MHz, MeOD-d_4): δ 170.26, 152.93, 138.04, 128.50, 128.24, 126.35, 61.15, 41.88, 39.57, 35.77, 34.68, 13.01.

2-(Phenethylamino)-1,4,5,6-tetrahydropyrimidine-5-carboxylic acid hydrochloride (**10**). **33** (51 mg, 0.185 mmol, 1 eq) was heated at 80 °C in HCl (aq., 4M) overnight and the solvent was removed under vacuum to afford a colourless film. The resulting film was triturated with Et_2O to give the product as an off-white solid (50 mg, 95%).^{1}H-NMR (600 MHz; MeOD-d_4): δ 7.32 (t, J = 7.5 Hz, 2H), 7.31–7.22 (m, 3H), 3.59–3.50 (m, 4H), 3.42 (t, J = 7.0 Hz, 2H), 3.03 (t, J = 4.8 Hz, 1H), 2.87 (t, J = 7.0 Hz, 2H). ^{13}C-NMR (151 MHz, MeOD-d_4): δ 171.90, 152.87, 138.03, 128.58, 128.26, 126.35, 41.97, 39.74, 35.60, 34.72. mp: (decomp.) > 167 °C. Purity by anal. HPLC (254 nm): 98%.

General procedure for dihydropyrimidine synthesis (**11**–**14**). Unless otherwise stated, in a flame dried flask the substituted or unsubstituted 2-aminopyrimidine-5-carboxylic acid (**34**–**37**) (1 eq) was suspended in dry DCM and put under N_2 atmosphere. TFA (15–20 eq) and Et_3SiH (7.5–10 eq) were added via syringe and the reaction was allowed to stir for 1–24 hr. The product was obtained by either adding Et_2O to the reaction mixture followed by filtration and drying, or by evaporating the solvent and washing the resulting solid with Et_2O followed by drying, to afford the product as a TFA salt.

2-Amino-1,6-dihydropyrimidine-5-carboxylic acid TFA (**11**). Synthesized according to general procedure for dihydropyrimidine synthesis using **34** (200 mg, 1.44 mmol, 1 eq), TFA (1.6 mL, 21.5 mmol, 15 eq) and Et_3SiH (1.6 mL, 10.8 mmol, 7.5 eq), stirred for 24 hr followed by evaporation of organic solvent, yielding the product as a white solid (323 mg, 88%). ^{1}H-NMR (400 MHz; DMSO-d_6): δ 12.60–12.54 (m, 1H), 9.89–9.82 (m, 1H), 8.56–8.47 (m, 1H), 7.98–7.89 (m,

2H), 7.18 (s, 1H), 4.00–3.98 (m, 2H). ^{13}C-NMR (101 MHz, DMSO-d_6): δ 165.97, 152.28, 133.05, 103.89, 38.84. mp: 164–167 °C. Purity by anal. HPLC (210 nm): 99%.

2-Amino-4-methyl-1,6-dihydropyrimidine-5-carboxylic acid TFA (**12**). Synthesized according to general procedure for dihydropyrimidine synthesis using **35** (200 mg, 1.05 mmol, 1 eq), TFA (1.6 mL, 21.5 mmol, 20 eq) and Et_3SiH (1.6 mL, 10.8 mmol, 10 eq), stirred for 5 hr followed by evaporation of organic solvent, yielding the product as a white solid (261 mg, 92%). ^{1}H-NMR (600 MHz; MeOD-d_4): δ 4.11 (d, J = 1.3 Hz, 2H), 2.34 (t, J = 1.2 Hz, 3H). ^{13}C-NMR (151 MHz, MeOD-d_4): δ 166.50, 152.48, 143.68, 117.70, 115.76, 99.78, 39.26, 16.00. mp: 151–155 °C. Purity by anal. HPLC (210 nm): 99%.

2-Amino-4-propyl-1,6-dihydropyrimidine-5-carboxylic acid TFA (**13**). Synthesized according to general procedure for dihydropyrimidine synthesis using **36** (100 mg, 0.459 mmol, 1 eq), TFA (0.5 mL, 6.89 mmol, 15 eq) and Et_3SiH (0.5 mL, 3.44 mmol, 7.5 eq), stirred for 1 hr and precipitated by addition of Et_2O, yielding the product as a white solid (137 mg, 99%). ^{1}H-NMR (600 MHz; MeOD-d_4): δ 4.12 (s, 2H), 2.75 (dd, J = 8.6, 6.9 Hz, 2H), 1.66–1.62 (m, 2H), 1.01 (t, J = 7.4 Hz, 3H). ^{13}C-NMR (151 MHz, MeOD-d_4): δ 166.20, 152.72, 147.55, 117.77, 115.83, 99.85, 39.27, 31.85, 20.99, 12.52. mp: 118–123°C. Purity by anal. HPLC (210 nm): 99%.

2-Amino-4-isopropyl-1,6-dihydropyrimidine-5-carboxylic acid TFA (**14**). Synthesized according to general procedure for dihydropyrimidine synthesis using **37** (100 mg, 0.459 mmol, 1 eq), TFA (0.5 mL, 6.89 mmol, 15 eq) and Et_3SiH (0.5 mL, 3.44 mmol, 7.5 eq), stirred for 1 hr and precipitated by addition of Et_2O, yielding the product as a white solid (115 mg, 84%). ^{1}H-NMR (600 MHz; MeOD-d_4): δ 4.28 (dt, J = 14.1, 7.1 Hz, 1H), 4.12 (s, 2H), 1.19 (d, J = 8.5 Hz, 6H). ^{13}C-NMR (151 MHz, MeOD-d_4): δ 166.18, 153.21, 151.21, 117.74, 115.80, 98.97, 39.34, 26.89, 18.02. mp: 137–138 °C. Purity by anal. HPLC (210 nm): 99 %.

2-Amino-4-methyl-1,4,5,6-tetrahydropyrimidine-5-carboxylic acid hydrochloride (**15**). **38** (600 mg, 3.31 mmol, 1 eq) was dissolved in MeOH (150 mL) and AcOH (1 mL) and Pd/C (10 wt.%, 1.05 g, 0.993 mmol, 0.3 eq) was added. The reaction was hydrogenated for 18 hours after which it was filtered and evaporated. The residue was taken up in HCl (aq., 4M) and heated at 80°C for 3 days. The solvent was evaporated to give a off-white solid (546 mg, 85% in two steps). ^{1}H-NMR (600 MHz; D_2O): δ 3.85–3.81 (m, 1H), 3.36 (d, J = 7.8 Hz, 2H), 2.78 (dt, J = 7.7, 4.8 Hz, 1H), 1.92 (s, 6H) (AcOH), 1.09 (d, J = 6.7 Hz, 3H). ^{13}C-NMR (151 MHz, D_2O): δ 179.38 (AcOH), 177.21, 153.19, 45.81, 42.05, 37.02, 22.01 (AcOH), 16.81. mp: (decomp.) > 202 °C.

2-Amino-4-propyl-1,4,5,6-tetrahydropyrimidine-5-carboxylic acid hydrochloride (**16**). **39** (60 mg, 0.287 mmol, 1 eq) was dissolved in MeOH (15 mL) and AcOH (0.1 mL) and Pd/C (10 wt.%, 100 mg, 0.094 mmol, 0.3 eq) was added. The reaction was hydrogenated for 3 days after which it was filtered and evaporated. The residue was taken up in HCl (aq., 4M) and heated at 80°C overnight. The solvent was evaporated to give a colourless film which was triturated with Et_2O to give the product as a white solid (60 mg, 94% in two steps). ^{1}H-NMR (600 MHz; MeOD-d_4): δ 3.76 (dt, J = 8.6, 4.4 Hz, 1H), 3.51 (d, J = 6.7 Hz, 2H), 3.07 (q, J = 5.5 Hz, 1H), 1.60–1.49 (m, 3H), 1.49–1.40 (m, 1H), 0.99 (t, J = 10.7 Hz, 3H). ^{13}C-NMR (151 MHz, MeOD-d_4): δ 171.31,

153.70, 49.72, 39.07, 37.85, 33.46, 18.64, 12.66. mp: 169–165 °C. Purity by anal. HPLC (210 nm): 96%.

2-Amino-4-isopropyl-1,4,5,6-tetrahydropyrimidine-5-carboxylic acid hydrochloride (**17**). **40** (60 mg, 0.287 mmol, 1 eq) was dissolved in MeOH (15 mL) and AcOH (0.1 mL) and Pd/C (10 wt.%, 100 mg, 0.094 mmol, 0.3 eq) was added. The reaction was hydrogenated for 3 days after which it was filtered and evaporated. The residue was taken up in HCl (aq., 4M) and heated at 90°C overnight. The solvent was evaporated to yield the product as a white solid (63 mg, 99% in two steps). ^{1}H-NMR (600 MHz; MeOD-d_4): δ 3.63 (dd, J = 13.1, 4.2 Hz, 1H), 3.51 (dd, J = 13.1, 5.0 Hz, 1H), 3.34 (dd, J = 6.8, 2.6 Hz, 1H), 3.15 (d, J = 3.9 Hz, 1H), 2.00–1.94 (m, 1H), 1.07 (d, J = 9.5 Hz, 6H). ^{13}C-NMR (151 MHz, MeOD-d_4): δ 171.69, 154.48, 57.00, 40.33, 37.49, 29.83, 18.46, 18.42. mp: 199–201°C. Purity by anal. HPLC (210 nm): 99%.

l-**15a**,**b** and *u*-**15a**,**b**. **35** (517 mg, 2.73 mmol, 1 eq) was suspended in EtOH (100 mL). Pd/C (10 wt.%, 330 mg, 0.310 mmol, 0.1 eq) was added and the reaction was hydrogenated for 28 hr, after which it was filtered and evaporated *in vacuo* to give a colourless film. Purification by chiral preparative HPLC (isocratic 30:70 EtOH/H_2O (NH_4OAc (20 mM, pH=4, aq.)) over 20 min) afforded the four enantiomers as white solids. *l*-**15a**,**b** (17 mg, 4%, 59 mg, 14%). ^{1}H-NMR (600 MHz; D_2O): δ 3.85–3.81 (m, 1H), 3.36 (d, *J* = 7.8 Hz, 2H), 2.78 (dt, *J* = 7.7, 4.8 Hz, 1H), 1.92 (s, 6H) (AcOH), 1.09 (d, *J* = 6.7 Hz, 3H). ^{13}C-NMR (151 MHz, D_2O): δ 179.38 (AcOH), 177.21, 153.19, 45.81, 42.05, 37.02, 22.01 (AcOH), 16.81. mp: (decomp.) > 202 °C. *u*-**15a**,**b** (7.3 mg, 1.7%, 12 mg, 3%). ^{1}H-NMR (600 MHz; D_2O): δ 3.61 (dd, *J* = 7.9, 6.5 Hz, 1H), 3.44 (dd, *J* = 12.6, 4.7 Hz, 1H), 3.31 (dd, *J* = 12.6, 8.9 Hz, 1H), 2.35 (ddd, *J* = 8.9, 8.0, 4.7 Hz, 1H), 1.92 (d, *J* = 10.2 Hz, 11H) (AcOH), 1.19 (d, *J* = 6.5 Hz, 3H). ^{13}C-NMR (151 MHz, D_2O): δ 177.48 (AcOH), 176.18, 151.19, 45.53, 44.10, 37.88, 20.03 (AcOH), 17.15. mp: (decomp.) > 186 °C.

Ethyl (E)-2-amino-4-styrylpyrimidine-5-carboxylate (**41**). **38** (700 mg, 3.86 mmol, 1 eq) was dissolved in glacial acetic acid (4 mL) and benzaldehyde (820 mg, 7.73 mmol, 2 eq) was added. The mixture was heated in a closed vial at 130 °C for 48 hr. After cooling to r.t. the product was precipitated by addition of methanol. The residue was reprecipitated from EtOAc/heptane (344 mg, 46%). ^{1}H-NMR (400 MHz; $CDCl_3$): δ 8.90 (s, 1H), 8.22 (d, J = 15.7 Hz, 1H), 8.02 (d, J = 15.7 Hz, 1H), 7.65–7.62 (m, 2H), 7.41–7.32 (m, 3H), 5.47–5.40 (m, 2H), 4.41–4.34 (m, 2H), 1.43–1.37 (m, 3H). ^{13}C-NMR (151 MHz, $CDCl_3$): δ 165.21, 164.38, 163.03, 162.45, 139.07, 136.16, 129.39, 128.75, 128.10, 123.72, 112.27, 60.86, 14.36.

Ethyl (E)-4-(2-([1,1'-biphenyl]-4-yl)vinyl)-2-aminopyrimidine-5-carboxylate (**42**). **38** (700 mg, 3.86 mmol, 1 eq) was dissolved in glacial acetic acid (4 mL) and [1,1'-biphenyl]-4-carbaldehyde (1.4 g, 7.73 mmol, 2 eq) was added. The mixture was heated in a closed vial at 130 °C for 48 hr. After cooling to r.t. the product was precipitated by addition of methanol. The residue was reprecipitated from EtOAc/heptane (502 mg, 38%). ^{1}H-NMR (400 MHz; $CDCl_3$): δ 8.90 (s, 1H), 8.29 (d, J = 15.7 Hz, 1H), 8.08 (d, J = 15.7 Hz, 1H), 7.74 (d, J = 8.2 Hz, 2H), 7.65 (d, J = 8.1 Hz, 4H), 7.48 (t, J = 7.5 Hz, 2H), 7.39 (t, J = 7.3 Hz, 1H), 5.83–5.79 (m, 2H), 4.41 (q, J = 7.1 Hz, 2H), 2.13 (s, 2H), 1.44 (t, J = 7.1 Hz, 3H). ^{13}C-NMR (151 MHz, $CDCl_3$): δ 176.03, 164.96, 164.91,

162.76, 161.43, 142.24, 140.36, 139.17, 135.06, 128.85, 128.66, 127.66, 127.42, 127.01, 123.40, 111.81, 60.93, 21.04, 14.36.

2-Amino-4-phenethyl-1,4,5,6-tetrahydropyrimidine-5-carboxylic acid hydrochloride (*l*-**18**). **41** (60 mg, 0.223 mmol, 1 eq) was dissolved in MeOH (15 mL) and AcOH (0.1 mL). Pd/C (10 wt.%, 110 mg, 0.103 mmol, 0.5 eq) was added and the reaction was hydrogenated for 2 days after which it was filtered and evaporated to give a colourless film. The residue was taken up in HCl (aq., 4M) and heated at 80°C overnight. The solvent was evaporated to afford the product as *l*-18 an off-white solid (49 mg, 89% over 2 steps). ^{1}H-NMR (600 MHz; MeOD-d_4): δ 7.33–7.19 (m, 5H), 3.81–3.73 (m, 1H), 3.59–3.50 (m, 2H), 3.14–3.09 (m, 1H), 2.87–2.80 (m, 1H), 2.77–2.71 (m, 1H), 1.93–1.85 (m, 2H). ^{13}C-NMR (151 MHz, MeOD-d_4): δ 171.18, 153.81, 140.64, 128.20, 127.94, 125.87, 49.54, 38.87, 38.10, 33.35, 31.41. mp: (decomp.) > 200 °C. Purity by anal. HPLC (210 nm): 97%.

4-(2-([1,1'-Biphenyl]-4-yl)ethyl)-2-amino-1,4,5,6-tetrahydropyrimidine-5-carboxylic acid hydrochloride (*l*-**19**). **42** (60 mg, 0.174 mmol, 1 eq) was dissolved in MeOH (15 mL) and AcOH (0.1 mL). Pd/C (10 wt.%, 110 mg, 0.103 mmol, 0.6 eq) was added and the reaction was hydrogenated for 2 days after which 0.1 eq Pd/C was added and hydrogenation was continued another day. At completion, the reaction mixture was filtered and evaporated to give a white solid. The residue was taken up in HCl (aq., 4M) and heated at 80°C overnight. The solvent was evaporated and the resulting solid was filtered and washed with cold MeOH affording the product as a white solid (16 mg, 60% over two steps). ^{1}H-NMR (600 MHz; MeOD-d_4): δ 7.61–7.60 (m, 2H), 7.58–7.57 (m, 2H), 7.44–7.40 (m, 2H), 7.38–7.29 (m, 3H), 3.85–3.77 (m, 1H), 3.61–3.51 (m, 2H), 3.16–3.10 (m, 1H), 2.93–2.85 (m, 1H), 2.84–2.76 (m, 1H), 1.98–1.90 (m, 2H). ^{13}C-NMR (151 MHz, MeOD-d_4): δ 171.26, 154.90, 153.83, 140.78, 139.75, 139.16, 128.45, 128.41, 126.77, 126.40, 49.56, 38.94, 38.16, 33.24, 31.01. mp: (decomp.) > 215 °C. Purity by anal. HPLC (254 nm): 97%.

REFERENCES

1. Humphrey, W., Dalke, A. & Schulten, K. VMD: Visual molecular dynamics. *J. Mol. Graph.* **14**, 33–38 (1996).
